# Triterpenoid–PEG Ribbons Targeting Selectivity in Pharmacological Effects

**DOI:** 10.3390/biomedicines9080951

**Published:** 2021-08-03

**Authors:** Zulal Özdemir, Uladzimir Bildziukevich, Martina Čapková, Petra Lovecká, Lucie Rárová, David Šaman, Michala Zgarbová, Barbora Lapuníková, Jan Weber, Oxana Kazakova, Zdeněk Wimmer

**Affiliations:** 1 Department of Chemistry of Natural Compounds, University of Chemistry and Technology in Prague, Technická 5, 16628 Prague 6, Czech Republic; zulalozdemr@gmail.com (Z.Ö.); vmagius@gmail.com (U.B.); m.capkova.bigy@seznam.cz (M.Č.); 2 Isotope Laboratory, Institute of Experimental Botany of the Czech Academy of Sciences, Vídeňská 1083, 14220 Prague 4, Czech Republic; 3 Department of Biochemistry and Microbiology, University of Chemistry and Technology in Prague, Technická 5, 16628 Prague 6, Czech Republic; loveckap@vscht.cz; 4 Department of Experimental Biology, Faculty of Science, Palacký University, Šlechtitelů 27, 78371 Olomouc, Czech Republic; lucie.rarova@upol.cz; 5 Institute of Organic Chemistry and Biochemistry of the Czech Academy of Sciences, Flemingovo náměstí 2, 16610 Prague 6, Czech Republic; nmrsaman@gmail.com (D.Š.); michala.zgarbova@uochb.cas.cz (M.Z.); barbora.lapunikova@uochb.cas.cz (B.L.); jan.weber@uochb.cas.cz (J.W.); 6 Ufa Institute of Chemistry of the Ufa Federal Research Centre of the Russian Academy of Sciences, 71, pr. Oktyabrya, 450054 Ufa, Russia; obf@anrb.ru

**Keywords:** triterpenoid, molecular ribbon, Huisgen 1,3-dipolar cycloaddition, amide bond, multifunctional PEG3 derivative, antimicrobial activity, anti-HIV activity, cytotoxicity, supramolecular self-assembly

## Abstract

(1) Background: To compare the effect of selected triterpenoids with their structurally resembling derivatives, designing of the molecular ribbons was targeted to develop compounds with selectivity in their pharmacological effects. (2) Methods: In the synthetic procedures, Huisgen 1,3-dipolar cycloaddition was applied as a key synthetic step for introducing a 1,2,3-triazole ring as a part of a junction unit in the molecular ribbons. (3) Results: The antimicrobial activity, antiviral activity, and cytotoxicity of the prepared compounds were studied. Most of the molecular ribbons showed antimicrobial activity, especially on *Staphylococcus aureus*, *Pseudomonas aeruginosa,* and *Enterococcus faecalis*, with a 50–90% inhibition effect (*c* = 25 µg·mL^−1^). No target compound was effective against HSV-1, but **8a** displayed activity against HIV-1 (EC_50_ = 50.6 ± 7.8 µM). Cytotoxicity was tested on several cancer cell lines, and **6d** showed cytotoxicity in the malignant melanoma cancer cell line (G-361; IC_50_ = 20.0 ± 0.6 µM). Physicochemical characteristics of the prepared compounds were investigated, namely a formation of supramolecular gels and a self-assembly potential in general, with positive results achieved with several target compounds. (4) Conclusions: Several compounds of a series of triterpenoid molecular ribbons showed better pharmacological profiles than the parent compounds and displayed certain selectivity in their effects.

## 1. Introduction

**Figure 1:**
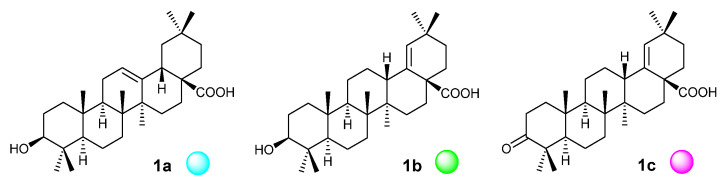
Structures of oleanolic acid (**1a**), morolic acid (**1b**), and moronic acid (**1c**).

Triterpenoids represent plant secondary metabolites with increasing importance due to the spectra of their pharmacological activity and ability to self-assemble into the supramolecular systems, often forming gels in different solvents, including aqueous media [1]. Oleanolic acid (**1a**), morolic acid (**1b**), and moronic acid (**1c**) are triterpenoids of the oleanane series, bearing a 6-6-6-6-6 pentacyclic skeleton (Figure 1). While oleanolic acid has been one of the most frequently investigated triterpenoids, morolic and moronic acid have been so far much less studied.

Oleanolic acid (**1a**) has been isolated from several hundred of plant species, and it is frequently used in the pharmaceutical and food industry, as reviewed recently [2]. The most important source of **1a** is plants of the genus *Oleaceae* [2]. Due to its lipophilic nature, it appears mainly in olive oil, but it is also present in the olive leaves [3]. Oleanolic acid (**1a**) has been proven to display inhibition activity against HIV and HSV [4,5,6]. It shows important antimicrobial activity, namely against G+ bacteria (*Staphylococcus aureus*, *S. epidermidis*, *Enterococcus faecalis*, *E. faecium*, *Streptococcus pneumoniae,* or *Bacillus subtilis*) [7,8,9]. It is also active against *Mycobacterium tuberculosis*, a Gram staining-type of pathogenic bacterium [10]. Its activity against G– bacteria is low or nil [11]. Oleanolic acid (**1a**) also displays an antidiabetic effect [12]. One of the possible mechanisms consists in the inhibition of glycogen phosphorylase, which is an enzyme capable of liberating glucose from glycogen, and the glucose level in blood is affected [13]. The other possible mechanism is connected with a possible inhibition of proteintyrosine phosphatase (PTP-1B), which is a part of a de-phosphorylation process of the insulin receptor [14]. The last but not least possible mechanism consists of a direct support of insulin secretion [15]. Oleanolic acid (**1a**) has been proven to display an anti-inflammatory effect, either due to the inhibition of transaminases or due to the inhibition of histamine secretion from mastocytes [16], and it also shows antitumor activity, as proven in several tumor cell lines [17]. It is also active against hepatitis of the C type through depressing of the gene types HCV-1b and 2a-JFH1 virus replication [18,19,20], and it inhibits HCV proteases [18]. However, the mechanism has not yet been clarified [19].

Morolic acid (**1b**) was first isolated from the wood of *Mora excelsa* in 1950 [21], and moronic acid (**1c**) was found in the roots of *Ozoroa mucronata* in 1979 [22]. Later on, both triterpenoids were found in other plants as well, often together [23]. Morolic acid (**1b**) and moronic acid (**1c**) are active against HIV and HSV [24]. Their antimicrobial activity is different: **1c** is more potential than **1b** in the number of affected microorganisms [25]. They also display anti-hypertensive and hypoglycemic effects [26]. Antidiabetic effect is explained either by a mechanism of inhibition of tyrosin phosphatase (PTP-1B; cf. above) [27] or by inhibiting 11β-hydroxysteroid dehydrogenase 1 (11β-HSD 1) that is a mediator of intracellular production of cortisol [28]. Both triterpenoids also display anti-inflammatory effect. Moronic acid (**1c**) was proven to show inhibition of tumor cells, in contrast to **1b** [29]. Moronic acid (**1c**) can also be synthesized in several steps from betulin, which is a lupane-type triterpenoid [30,31].

Pentacyclic triterpenoids have been used to control biofilm-related infections caused by *S. aureus* and other pathogens [32]. Patients, which were intubated due to the SARS-CoV-2 infection nowadays, often suffer from infections caused by microorganisms forming bacterial films that occur in the intubation equipment [33,34]. Therefore, an investigation of novel antimicrobial agents potentially capable of controlling bacterial films may contribute to augmenting the health of the patients seriously infected with SARS-CoV-2. These findings make this investigation even more important, because the designed molecular ribbons are based solely on natural products from sustainable sources, and their pharmacological effect is targeted to enhance the effect of their parent triterpenoids.

Since plant triterpenoids can also be found in vegetables, medicinal plants, spices, etc., they become natural constituents of the human diet. The literature data show that the average daily intake varies between 250 and 400 mg per day in humans, depending on the specific part of the world [35]. This is an important item of information in consequence with the different types of pharmacological activity resulting from this investigation.

The objectives of the presented investigation consisted of several partial tasks. (a) The first is to design procedures for the synthesis of selected molecular ribbons based on the selected triterpenoids connected through the multifunctional PEG3 junction units, and by the newly formed amide bonds and the 1,4-disubstituted 1,2,3-triazole rings. Generally, PEG-mediated motifs have been used in pharmacologically active compounds, such as steroids [36], alkaloids [37], or triterpenoids [38,39]. A modification of biomolecules by the PEG motifs resulted in compounds having a longer blood-circulation time than the corresponding parent biomolecules [40,41]. A formation of a covalent bond between the PEG spacer and the biologically active compounds is one of the promising techniques to design novel drug molecules with improving therapeutic effect [40,41]. Due to the ester-based protecting groups to be employed during the synthetic procedures described here, using any other ester group in the target molecules is excluded, while a combination of amide bonds and 1,4-disubstituted 1,2,3-triazole rings does not interfere with introducing and removing any protecting group. (b) The second objective is to target the investigation on antimicrobial and antiviral activity, and cytotoxicity screening tests of the triterpenoid molecular ribbons to show their advantages and/or disadvantages in comparison with their parent single triterpenoid molecules. (c) The third objective is to investigate supramolecular characteristics of the target molecular ribbons and to evaluate the importance of the formed supramolecular systems for potential pharmacological applications in comparison with their parent triterpenoids.

## 2. Materials and Methods

### 2.1. General

The NMR measurements (Bruker, Berlin, Germany) were performed on a Bruker AVANCE II 600 MHz spectrometer equipped with a 5 mm TCI cryoprobe in a 5 mm tube in different solvents. The ^1^H NMR and the ^13^C NMR spectra were recorded at 600.13 MHz and 150.90 MHz in CDCl_3_ or CD_3_OD using tetramethylsilane (δ = 0.0–CDCl_3_) or signal of solvent (δ = 3.31 or 49.50 for ^1^H/^13^C–CD_3_OD) as internal references. ^1^H NMR data are presented in the following order: chemical shift (δ) expressed in ppm, multiplicity (s, singlet; d, doublet; t, triplet; q, quartet; m, multiplet), number of protons, coupling constants in Hertz. For unambiguous assignment of both ^1^H and ^13^C signals, 2D NMR, ^1^H,^13^C gHSQC, and gHMBC spectra were measured using standard parameters sets and pulse programs delivered by the producer of the spectrometer. Infrared spectra were measured with a Nicolet iS-5 FT-IR spectrometer (Thermofisher Scientific, Waltham, MA, USA). UV-VIS spectra were measured on a Specord 210 spectrometer (Jena Analytical, Germany). TLC was carried out on silica gel plates (Merck 60F_254_), and the visualization was performed by both the UV detection and spraying with the methanolic solution of phosphomolybdic acid (5%), followed by heating. For column chromatography, silica gel 60 (0.063–0.200 mm) from Merck was used. Analytical HPLC was carried out on a TSP (Thermoseparation Products, Boston, MA, USA) instrument equipped with a ConstaMetric 4100 Bio pump and a SpectroMonitor 5000 UV DAD. The analyses of the products were performed on a reverse phase Nucleosil 120-5 C18 column (250 × 4 mm; Watrex, Prague, Czech Republic) using a methanol/water mixture (9:1, *v*/*v*) as the mobile phase at 0.5 to 1.0 mL.min^−1^. The eluate was monitored at 220, 254, and 275 nm, and the UV spectra were run from 200 to 300 nm. All compounds were purified until their purity was >99%. Their analytical data are summarized in the Supplementary material, part 1, Synthetic procedures and analytical data. All chemicals and solvents were purchased from regular commercial sources in analytical grade, and the solvents were purified by general methods before use. Triterpenoids were purchased from Dr. Jan Šarek–Betulinines (www.betulinines.com, (accessed on 14 June 2021).

### 2.2. Synthetic Procedures

#### 2.2.1. Procedure A

Benzyl bromide (2.5 eq) and potassium carbonate (3 eq) were added to a solution of the starting compound (1 eq) in DMF (15 mL), and the reaction mixture was stirred at r.t. for 24 h. The mixture was washed with brine, the organic layer was extracted with benzene, and then, it was dried over sodium sulfate. The solid phase was filtered off, and the solvent was evaporated under reduced pressure. The crude residue was purified by column chromatography on silica gel, using a chloroform/ethanol mixture (200:1) as a mobile phase.

#### 2.2.2. Procedure B

Sodium hydride (8 eq) was added to a solution of the starting compound (1 eq) in THF (20 mL), and the reaction mixture was stirred at r.t. for 1 h. Then, an additional amount of sodium hydride (8 eq) was added, and stirring continued for an additional 2 h. Finally, propargyl bromide (3 eq) was added, and the reaction mixture was stirred at r.t. for 7 days. The solvent was evaporated, and the residue was washed with brine and extracted with chloroform. After drying over sodium sulfate, the solvent was evaporated, and the crude residue was purified by column chromatography on silica gel, using chloroform as a mobile phase.

#### 2.2.3. Procedure C

Acetic anhydride (1.44 eq), EDIPA (1.8 eq), and DMAP (0.13 eq) were added to a solution of the starting compound (1 eq) in THF (20 mL). The reaction mixture was stirred at 100 °C for 3 h. Then, an additional amount of acetic anhydride (0.77 eq) and EDIPA (0.9 eq) were added, and heating continued for an additional 1 h. The reaction was stopped by adding water. The organic layer was extracted with chloroform, and the extract was washed with saturated solution of sodium bicarbonate and dried over sodium sulfate. After filtering off the solid phase, solvents were evaporated, and the crude residue was purified by column chromatography on silica gel, using a chloroform/ethanol mixture (100:1) as a mobile phase.

#### 2.2.4. Procedure D

Propargyl bromide (1.5 eq) and potassium carbonate (3 eq) were added to a solution of the starting compound in DMF (15 mL). The reaction mixture was stirred at r.t. for 24 h. It was quenched with brine and extracted with benzene. After drying the extract over sodium sulfate, the solid phase was filtered off, and the solvent was evaporated. The crude residue was purified by column chromatography on silica gel, using a chloroform/petroleum ether mixture (1:3) as a mobile phase.

#### 2.2.5. Procedure E

Oxalyl chloride (7 eq) was added to a solution of the starting compound (1 eq) in DCM (30 mL), and the reaction mixture was stirred at r.t. for 3 h. The solvent was evaporated under reduced pressure, and the residue was re-dissolved in freshly distilled DCM (30 mL). Azido-PEG3-amide (1.1 eq) and EDIPA (2.5 eq) were added, and the reaction mixture was stirred at r.t. for 24 h. The reaction mixture was quenched with brine, extracted with chloroform, and the extract was dried over sodium sulfate. The solid phase was filtered off, and the solvents were evaporated under reduced pressure. The residue was purified by column chromatography on silica gel using a chloroform/ethanol mixture (200:1) as a mobile phase.

#### 2.2.6. Procedure F

The starting compounds bearing either a propargyl group (1 eq) or azide group (1.05 eq) were dissolved in DCM (30 mL). Then, a mixture of aqueous solution of copper sulfate pentahydrate and TBTA (0.05 M) was added, which was followed by the addition of sodium ascorbate (1 eq). The reaction mixture was stirred at r.t. for 24 h, and then, it was quenched with water. The organic layer was extracted with chloroform, and the extract was dried over sodium sulfate. The solid phase was filtered off, and the solvent was evaporated under reduced pressure. The crude residue was purified by column chromatography on silica gel, using a chloroform/ethanol mixture (100:1) as a mobile phase.

#### 2.2.7. Procedure G

Catalyst (10% Pd/C, 0.67 eq) was added to a solution of the starting compound (1 eq) in a mixture of ethanol/THF (1:1, 30 mL) in a bottle equipped with a septum. Air was removed from the bottle, it was filled with hydrogen, and the reaction mixture was stirred at r.t. for 24 h. To stop the hydrogenation reaction, hydrogen was removed, and the catalyst was filtered off. Solvents were evaporated under reduced pressure, and the crude residue was purified by column chromatography on silica gel, using a chloroform/ethanol mixture (100:1) as a mobile phase.

#### 2.2.8. Procedure H

Lithium hydroxide monohydrate (5 eq) was added to a solution of the starting compound (1 eq) in methanol (30 mL). The reaction mixture was heated to 80 °C for 5 h. Then, the solvent was evaporated under reduced pressure, and the crude residue was purified by column chromatography on silica gel, using a chloroform/ethanol mixture (100:1) as a mobile phase.

### 2.3. Antimicrobial Screening Tests

The antimicrobial activity of **1a**–**1c**, **8a**, **12a**, **8b**, **12b**, **4c**, **6c**, **6d**, and **8d** was studied in vitro, using *Bacillus cereus*, *Escherichia coli*, *Pseudomonas aeruginosa*, *Staphylococcus aureus*, and *Enterococcus faecalis*. The resazurine test was used to evaluate this activity.

The cell inoculum was made in Mueller–Hinton broth (10 mL). Colonies of cells were inoculated from the solid medium that was cultivated for 24 h under optimum conditions. A part of the inoculum was diluted to get suspension with an optical density 0.6 to 0.9 M_c_F. An aliquot amount (1 mL) was taken and diluted with additional volume of the medium (19 mL). The stored solutions of the tested compounds were made at a concentration *c* = 200 µg.mL^−1^. The stored suspensions of the cells, MHB medium, solutions of the antibiotics specific for the given bacteria (vancomycin for *S. aureus* and *E. faecalis*, kanamycin for *E. coli* and *P. aeruginosa*, and tetracycline for *B. cereus*), and the solutions of the compounds were pipetted into the microtitration plates. Subsequently, a concentration sequence of the tested compounds was made (*c* = 100, 50, and 25 µg.mL^−1^). The content of the microtitration plates was cultivated for 24 h under optimum conditions for the given bacteria. A solution of resazurine was made, based on a dilution of its stored solution (*c* = 0.15 mg.mL^−1^; 2 mL) into the MHB medium (18 mL). Then, it was pipetted into the microtitration plates that were cultivated for an additional 1 h. Finally, fluorescence measurement (excitation at 560 nm, emission at 590 nm) was made, and the inhibition of the bacterium was determined.

### 2.4. Cytotoxicity Screening Tests and Cell Cultures Used

Description of the experimental procedure used in the cytotoxicity screening test was already published [42,43]. The following cancer cell lines were used in the investigation: T-lymphoblastic leukemia, CEM; breast carcinoma, MCF7; cervical carcinoma, HeLa; and malignant melanoma, G-361. Human foreskin fibroblasts, BJ, were used as reference cells. All of the above cell lines were obtained from the American Type Culture Collection (Manassas, VA, USA). African green monkey kidney Vero cells were obtained from the European Collection of Cell Cultures (Salisbury, UK) and human T-cell leukemia MT-4 cells were obtained through the NIH AIDS Reagent Program, Division of AIDS, NIAID, NIH, from Dr. Douglas Richman. All cells, but MT-4 cells, were cultured in DMEM (Dulbecco’s Modified Eagle Medium, Sigma, St. Louis, MO, USA). MT-4 cells were maintained in RPMI1640 medium. Media used were supplemented with 10% fetal bovine serum, 2 mM l-glutamine, and 1% penicillin–streptomycin (all Sigma, St. Louis, MO, USA). The cell lines were maintained under standard cell culture conditions at 37 °C and 5% CO_2_ in a humid environment. Cells were subcultured twice or three times a week using the standard trypsinization procedure.

### 2.5. Antiviral Tests

The anti-HIV-1 and anti-HSV-1 activity and cytotoxicity in MT-4 cells and Vero cells, respectively, were determined as previously described [44,45]. Three-fold dilution of the compounds were tested in triplicate, using HIV-1 (laboratory strain NL4-3) and HSV-1 (strain HF), respectively. Tenofovir alafenamide and acyclovir were used as controls. Compounds were not toxic up to 50 µM against MT-4 and Vero cells.

### 2.6. Supramolecular Self-Assembly Studies

Ability of the target compounds **8a**, **12a**, **8b**, **12b**, **4c**, **6c**, **6d,** and **8d** was investigated by (a) measuring UV-VIS absorption in methanol/water mixtures with a constant concentration of the studied compound, and (b) investigating their ability to form gel in different solvents.

#### 2.6.1. Ability to Form Supramolecular Gel

The solutions of the studied compounds **8a**, **12a**, **8b**, **12b**, **4c**, **6c**, **6d**, and **8d** (1%, *w*/*v*) in the appropriate solvent were mixed with ultrasound for 30 min, then heated to the boiling points of the used solvents, and then allowed to cool down to the r.t. for 24 h. An example of the resulting gels that were produced from **8a**, **4c**, **6c,** and **6d** is shown in the Supplementary Material, Figure S1.

#### 2.6.2. UV-VIS Spectrometry

The self-assembly of the selected compounds **8a**, **12a**, **8b**, **12b**, **4c**, **6c**, **6d**, and **8d** was measured using UV absorption in methanol/water mixtures with a constant concentration of the studied compound for 7 days in one-hour intervals on the first day, and in 24 h intervals all following days. The stock solutions of the studied compounds were prepared at a concentration of 1 mg·mL^−1^ in water. Then, a series of methanol/water mixtures were prepared starting from a methanol/water ratio 100/0 down to 0/100 in 20% steps. The stock solutions of the studied compounds (0.15 mL) were added separately to each vial containing methanol/water mixtures (3 mL), and the UV spectra were recorded in the wavelength range of 200–400 nm. The most remarkable changes in these spectra were observed for methanol/water ratios 40/60 and 60/40 in their mixtures. For the compounds **8a**, **12a**, **12b** and **6d**, the details are presented in the Supplementary Material; see Figure S2.

## 3. Results and Discussion

### 3.1. Synthetic Procedures

#### 3.1.1. Procedure A

**Scheme 1:**
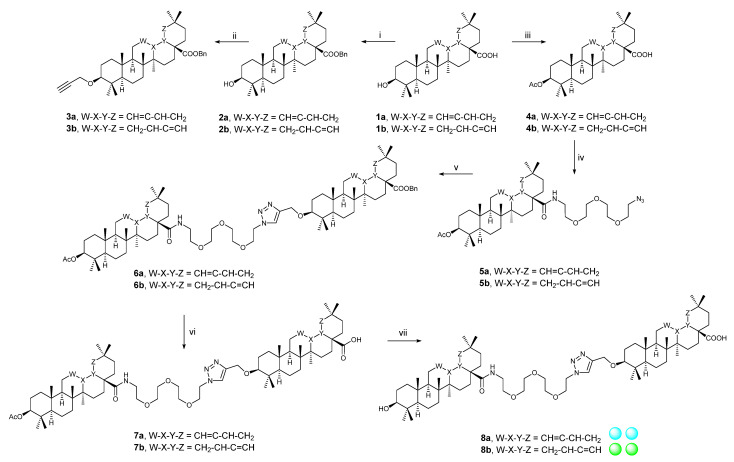
Synthetic procedure, part 1. Reagents and conditions. (i) BnBr, K_2_CO_3_, DMF, r.t., 24 h; (ii) NaH, propargyl bromide, THF, r.t., 7 days; (iii) Ac_2_O, EDIPA, DMAP, THF, 100 °C, 4 h; (iv) (a) oxalyl chloride, DCM, r.t., 3 h, (b) N_3_-PEG3-NH_2_, EDIPA, r.t., 24 h; (v) **3a**/**3b**, CuSO_4_·5H_2_O, TBTA, sodium ascorbate, DCM/H_2_O, r.t., 24 h; (vi) 10% Pd/C, H_2_, ethanol/THF (1:1), r.t., 24 h; (vii) LiOH·H_2_O, methanol, 80 °C, 5 h.

Esterification of the carboxyl group of the starting triterpenoid (**1a** or **1b**) was made by a substitution reaction using benzyl bromide in DMF under the presence of potassium carbonate [46]. Compounds **2a**/**2b** were prepared using the procedure A (Scheme 1).

#### 3.1.2. Procedure B

Synthesis of propargyl ether of the starting triterpenoid (**1a** or **1b**) consisted in a formation of the corresponding alcoholate using sodium hydride in THF, followed by its reaction with propargylic bromide [46]. Compounds **3a**/**3b** were prepared using the procedure B (Scheme 1).

#### 3.1.3. Procedure C

Esterification of the free hydroxyl group of the starting triterpenoid (**1a** or **1b**) was made with acetic anhydride in THF, using EDIPA as a base and DMAP as a reaction enhancer [43]. Compounds **4a**/**4b** were prepared using the procedure C (Scheme 1).

#### 3.1.4. Procedure D

**Scheme 2:**
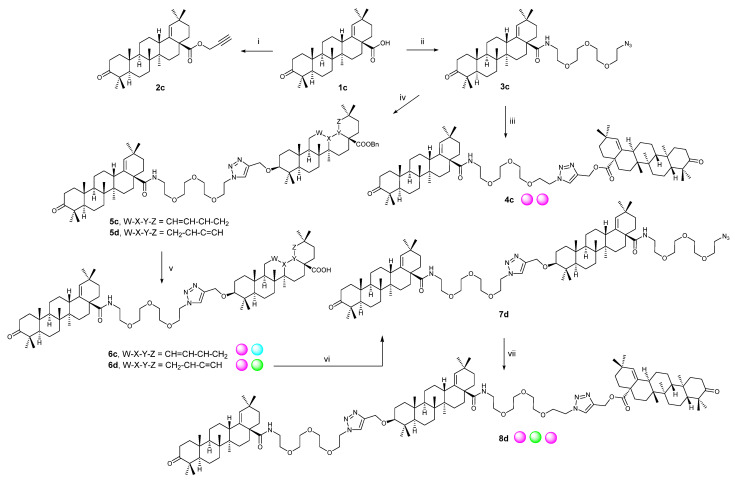
Synthetic procedure, part 2. Reagents and conditions. (i) propargyl bromide, K_2_CO_3_, DMF, r.t., 24 h; (ii) (a) oxalyl chloride, DCM, r.t., 3 h, (b) N_3_-PEG3-NH_2_, EDIPA, r.t., 24 h; (iii) **2c**, CuSO_4_·5H_2_O, TBTA, sodium ascorbate, DCM/H_2_O, r.t., 24 h; (iv) **3a**/**3b**, CuSO_4_·5H_2_O, TBTA, sodium ascorbate, DCM/H_2_O, r.t., 24 h; (v) 10% Pd/C, H_2_, ethanol/THF (1:1), r.t., 24 h; (vi) (a) oxalyl chloride, DCM, r.t., 3 h, (b) N_3_-PEG3-NH_2_, EDIPA, r.t., 24 h; (vii) **2c**, CuSO_4_·5H_2_O, TBTA, sodium ascorbate, DCM/H_2_O, r.t., 24 h.

Synthesis of propargyl ester of the starting triterpenoid (**1c**) was made by a substitution reaction of the carboxyl group of **1c** with propargylic bromide in DMF in the presence of potassium carbonate [47]. Compound **2c** was prepared using the procedure D (Scheme 2).

#### 3.1.5. Procedure E

**Scheme 3:**
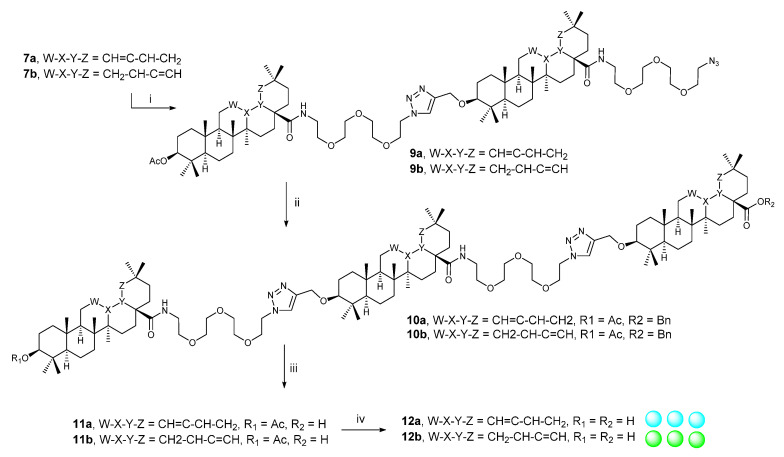
Synthetic procedure, part 3. Reagents and conditions. (i) (a) oxalyl chloride, DCM, r.t., 3 h, (b) N_3_-PEG3-NH_2_, EDIPA, r.t., 24 h; (ii) **3a**/**3b**, CuSO_4_·5H_2_O, TBTA, sodium ascorbate, DCM/H_2_O, r.t., 24 h; (iii) 10% Pd/C, H_2_, ethanol/THF (1:1), r.t., 24 h; (iv) LiOH·H_2_O, methanol, 80 °C, 5 h.

An amide bond formation was made by converting free carboxyl group of the starting triterpenoid (**1a**–**1c**) into the corresponding acyl chloride using oxalyl chloride in DCM, followed by its reaction with the corresponding amine in DCM using EDIPA as a base [43]. Compounds **5a**/**5b**, **9a**/**9b**, **3c**, and **7d** were prepared using the procedure E (Scheme 1, Scheme 2 and Scheme 3).

#### 3.1.6. Procedure F

Cu(I)-Catalyzed Huisgen 1,3-dipolar cycloaddition was made using components bearing either a propargylic group (**3a**/**3b**, **2c**) or azide group (**5a**/**5b**, **9a**/**9b**, **3c**, and **7d**). Cu(I) ions were generated in situ by reducing copper(II) sulfate by sodium ascorbate. TBTA was used as reaction initiator [46]. Compounds **6a**/**6b**, **10a**/**10b**, **4c**, **5c**/**5d**, and **8d** were prepared using the procedure F (Scheme 1, Scheme 2 and Scheme 3).

#### 3.1.7. Procedure G

Removing of the protecting benzyl group was made by palladium-catalyzed hydrogenation of the selected starting compound (**6a**/**6b**, **10a**/**10b**, and **5c**/**5d**) [46]. Compounds **7a**/**7b**, **11a**/**11b**, and **6c**/**6d** were prepared using the procedure G (Scheme 1, Scheme 2 and Scheme 3).

#### 3.1.8. Procedure H

Removing of the protecting acetyl group was made by alkaline hydrolysis of the esters (**7a**/**7b** and **11a**/**11b**) by lithium hydroxide in methanol [43]. Compounds **8a**/**8b** and **12a**/**12b** were prepared using the procedure H (Scheme 1 and Scheme 3).

### 3.2. Cytotoxicity

Cytotoxicity in the malignant melanoma cancer cell line (G-361) was only found for **6d** (IC_50_ = 20.0 ± 0.6 µM), even if all target compounds were subjected to the cytotoxicity screening tests. The compound **6d**, a hybrid molecular ribbon based on **1b** and **1c**, was inactive in CEM, MCF7, and HeLa cancer cell lines (IC_50_ > 50 µM), and was not toxic to the human fibroblasts (BJ; IC_50_ > 50 µM). Therefore, **6d** is selectively cytotoxic in the G-361 cancer cell line.

### 3.3. Antimicrobial Activity

Antimicrobial activity of the target compounds was tested on *Staphylococcus aureus*, *Escherichia coli*, *Bacillus cereus*, *Pseudomonas aeruginosa,* and *Enterococcus faecalis* using three different concentrations (*c* = 25, 50, 100 µg·mL^−1^). The results achieved with the lowest concentration of the tested compound (*c* = 25 µg·mL^−1^) are presented in Table 1. When higher concentrations of the tested compounds were used, inhibition of microorganism growth increased as well, even though for some compounds, certain variations were observed (cf. Supplementary Material, Table S1). The irregularity in the antimicrobial activity values in a dependence on the concentrations of the tested compounds may be connected with the supramolecular characteristics of the molecular ribbons (cf. Supplementary Material, Table S1). We had already observed such behavior in the investigation of cytotoxicity of another series of triterpenoid-based compounds [43,47]. The highest inhibition activity for *S. aureus* was observed when it is treated with **8a**, which is a dimer-like molecular ribbon based on **1a**. While **1a** was not active, dimer-like molecular ribbon **8a** showed increasing inhibition activity. However, trimer-like molecular ribbon **12a**, also based on **1a**, showed lower activity than **8a**, which can be caused either by a decrease in solubility or by size of the compound for possible penetration into the microorganism or by the effect of supramolecular self-assembly (cf. Supplementary Material). The same trend of inhibition activity was observed when *E. coli* was treated with **1a** or its ribbons. Interestingly, in the tests with *S. aureus*, when **1a** was replaced by **1b**, which only differs from **1a** by the position of the double bond in the skeleton, the formed trimer-like molecular ribbon **12b** exhibited the highest activity in comparison with **1b** and **8b**, presenting the importance of small structural variations on the inhibition activity. While **1c** was not active on *S. aureus,* its dimer-like molecular ribbon **4c** was slightly active. Hybrid dimer-like molecular ribbons **6c** and **6d** were synthesized as well, by combining **1c** with **1a** or **1b**. While the hybrid **6c** was less active than **8a**, hybrid **6d** was more active than **8d**. It can be assumed that the hybridization of **1c** (moronic acid) with **1b** (morolic acid) was more favorable than its hybridization with **1a** (oleanolic acid) when the inhibition activity results were taken into consideration.

In turn, for *B. cereus*, the highest inhibition activity was achieved when it is treated with **1a** or **1b** rather than by the trimer-like or dimer-like molecular ribbons, although **1c** (moronic acid) did not show any activity. Similar inhibition activities were observed with **1a** (oleanolic acid) and **1b** (morolic acid), showing the importance of the presence of the (C3)-OH group of triterpenoid for possible activity on *B. cereus*. This group is replaced with a ketone group in case of **1c** (moronic acid). In contrast to these results, dimer-like molecular ribbon **4c** showed the highest activity against *P. aeruginosa*, which is the only G– bacterial strain used in this investigation, while the other dimer-like molecular ribbons exhibited lower inhibition activity values than **4c**. Parent triterpenoids **1a**–**1c** were not active at all, which is in accordance with the literature data [11], demonstrating no or nil antimicrobial activity of this types of triterpenoids on the G– bacteria. The dimer-like molecular ribbon **4c** displayed much higher antimicrobial activity on *E. faecalis* than **1c** that showed no inhibition of the tested microorganisms. However, the highest activity was still observed when *E. faecalis* was treated with **1a**.

The target molecular ribbons are the new compounds, and no data on their pharmacological activity have been so far available in the literature. Therefore, the found results cannot be discussed in the context with the literature data, with the only exception presented above. The data have proven that most of the target molecular ribbons display higher inhibition of the studied microorganisms than their parent triterpenoids **1a**–**1c**. Table S1 (cf. Supplementary material) shows data on the dose–response dependence of antimicrobial activity of the target molecular ribbons **8a**, **12a**, **8b**, **12b**, **4c**, **6c**, **6d**, and **8d** versus their parent compounds **1a**–**1c**. All doses mentioned there at the maximal concentration used in the screening tests (*c* = 100 µg·mL^−1^) are much lower doses than humans may consume with plant-based food (vegetables, medicinal plants, spices, etc.) through which they may receive 250–400 mg per day in average [35].

### 3.4. Antiviral Activity

The only compound of this series (**8a**) showed low activity against HIV-1 in MT-4 (EC_50_ ± SE = 50.6 ± 7.8 µM). However, the found EC_50_ value is several orders of magnitude higher than those for reference compound, tenofovir alafenamide (EC_50_ ± SE = 0.0097 ± 0.0023 µM). No target compound displayed activity against HSV-1.

### 3.5. Gel formation and Supramolecular Self-Assembly

The target compounds were tested to show their ability to form supramolecular gels. This investigation was made because we have found a relationship between cytotoxicity and the ability of compounds to form supramolecular aggregates capable of affecting cytotoxicity [43,47], and similar results have recently been published by other authors [48,49]. Among the studied series of compounds, the only **4c**, **6c**, and **6d** produced a gel in glycerol; **8a** produced a gel in ethylene glycol (Supplementary material, Figure S1). In turn, self-assembly can be achieved with other target compounds based on spectral measurements. UV spectra measured in a mixture of water/methanol with changing ratio of both solvents in 20% steps, but containing a constant concentration of the studied compound, show irregularities in the absorbance maxima sequence or even in wavelengths of the signal maximum, if self-assembly takes place. This effect of irregularity in the absorbance maxima sequence was observed with several target compounds **8a** and **12a**. For more details, see the Supplementary Material (Supplementary material, Figure S2).

## 4. Conclusions

During this investigation, we have designed several molecular ribbons based on the combinations of oleanolic, morolic, and moronic acid (**1a**–**1c**) in the ribbons. The multifunctional PEG3 unit was used as a junction chain, together with the 1,2,3-triazole ring and the amide bond, between the triterpenoid molecules to form molecular ribbons. The synthetic procedures recently developed in the team were used, and some of them were optimized. The target structural ribbons were subjected to the antimicrobial and antiviral activity as well as cytotoxicity screening tests. The only target compound **6d** displayed mild and selective cytotoxicity against melanoma cells G-361 (IC_50_ = 20.0 ± 0.6 µM). The only compound **8a** showed low but again selective activity against HIV-1 in MT-4 cells (EC_50_ ± SE = 50.6 ± 7.8 µM). However, more target compounds displayed antimicrobial activity. Compounds were tested in three concentrations (*c* = 100, 50, and 25 µg·mL^−1^), and the complete results are presented in the Supplementary Material, Table S1, while selected results are shown in Table 1. Among the highest observed inhibition, **8a** inhibited *S. aureus* and *E. coli* in > 50% (*c* = 25 µg·mL^−1^), **12b** inhibited *S. aureus* in > 40% (*c* = 25 µg·mL^−1^), and **4c** inhibited *E. faecalis* in > 62% (*c* = 25 µg·mL^−1^). In turn, **1a** and **1b** inhibited *B. cereus* in > 77% (*c* = 25 µg·mL^−1^), and **1a** inhibited *E. faecalis* in > 87% (*c* = 25 µg·mL^−1^).

Since the inhibition of several types of microorganisms, including *S. aureus*, has been found, the molecular ribbons, namely **8a** and **12b**, displaying higher antimicrobial activity on *S. aureus* than their parent triterpenoids **1a** and **1b**, can also be considered for use in controlling bacterial films of *S. aureus* in the intubation equipment of patients suffering from complicated SARS-CoV-2 infections, as reported recently [33,34], to prevent additional microbial infection from the intubation equipment, using molecular ribbons based on the natural products (cf. Table 1 and Table S1 in the Supplementary Material).

Four target compounds (**4c**, **6c**, **6d**, and **8a**) formed gels either in glycerol or in ethylene glycol. Data from the UV spectrometry achieved under the described conditions gave proof that compounds **8a** and **12a** formed self-aggregated systems detected by the instrument in the methanol/water mixtures.

Several of the synthesized triterpenoid–PEG molecular ribbons gave evidence that the investigation of more complex structures based on plant triterpenoids can be important, because several of those structures either displayed enhancing pharmacological effects different from those shown by their parent monomeric triterpenoids or displayed existing supramolecular characteristics, including the ability to form supramolecular gels in contrast to their parent triterpenoids. However, a clear relation between the antimicrobial activity and supramolecular characteristics cannot be presented because we do not have enough indisputable data at present, and, therefore, this topic has been currently under more detailed investigation.

## Figures and Tables

**Table 1:** Selected data of the antimicrobial activity ^a,b^.

Microorganism	Compound	Inhibition [%] (*c* = 25 µg·mL^−1^) ^c^
*Staphylococcus aureus* (+)	**1a ●**	–
*Staphylococcus aureus*	**8a ●●**	**52 ± 7.8** ^e^
*Staphylococcus aureus*	**6c ●●**	13 ± 2.0
*Staphylococcus aureus*	**12a ●●●**	28 ± 4.2
*Staphylococcus aureus*	**1b ●**	21 ± 3.2
*Staphylococcus aureus*	**8b ●●**	–
*Staphylococcus aureus*	**6d ●●**	25 ± 3.8
*Staphylococcus aureus*	**12b ●●●**	**40 ± 6.0** ^e^
*Staphylococcus aureus*	**1c ●**	–
*Staphylococcus aureus*	**4c ●●**	11 ± 1.7
*Escherichia coli* (+)	**1a ●**	–
*Escherichia coli*	**8a ●●**	**51 ± 7.7** ^e^
*Escherichia coli*	**12a ●●●**	–
*Pseudomonas aeruginosa* (−)	**1c ●**	–
*Pseudomonas aeruginosa*	**4c ●●**	**50 ± 7.5** ^e^
*Enterococcus faecalis* (+)	**1a** ^d^**●**	**87 ± 13.0**
*Enterococcus faecalis*	**1b ●**	38 ± 5.7
*Enterococcus faecalis*	**1c ●**	–
*Enterococcus faecalis*	**4c ●●**	**62 ± 9.3** ^e^
*Enterococcus faecalis*	**8d ●●●**	38 ± 5.7
*Bacillus cereus* (+)	**1a** ^d^ **●**	**76 ± 11.4**
*Bacillus cereus*	**1b** ^d^ **●**	**78 ± 11.7**
*Bacillus cereus*	**1c ●**	–

^a^ A comparison of the antimicrobial activity of a target compound is always followed by the antimicrobial activity of its parent triterpenoid; ^b^ antibiotics (*c* = 25 µg·mL^−1^, causing 99% inhibition) were used as positive control: vancomycin for *S. aureus* and *E. faecalis*, kanamycin for *E. coli* and *P. aeruginosa*, and tetracycline for *B. cereus*); ^c^ hyphen represents no inhibition; ^d^ the highest inhibition of a microorganism found for **1a** and **1b** at the given concentration; ^e^ These molecular ribbons displayed higher inhibition of the microorganism than its parent triterpenoid(s).

## Data Availability

The data presented in this study are available on request from the corresponding author.

## Supplementary Materials

The following material is available online at https://www.mdpi.com/article/10.3390/biomedicines9080951/s1: Details on the synthetic procedures and analytical data of the prepared compounds, including the scanned NMR spectra of the target molecular ribbons, additional details on antimicrobial screening tests, and details on gel formation and other supramolecular characteristics.
