# Supplementary material for: Triterpenoid–PEG Ribbons Targeting Selectivity in Pharmacological Effects
Source: Biomedicines. 2021 Aug 3;9(8):951. doi: 10.3390/biomedicines9080951 (PMC8391127; doi:10.3390/biomedicines9080951)
Supplement: Supplementary file 1 [file biomedicines-09-00951-s001.zip › biomedicines-1284639-supplementary.pdf]

Supplementary material

Triterpenoid–PEG ribbons targeting selectivity in pharmacological effects

Zulal Özdemir,[1a,2,†] Uladzimir Bildziukevich,[1a,2,†] Martina Čapková,[1a,†] Petra Lovecká,[1b] Lucie Rárová,[3#] David Šaman,[4] Michala Zgarbová,[4,#] Barbora Lapuníková,[4,#] Jan Weber,[4] Oxana Kazakova [5] and Zdeněk Wimmer *,[1a,2]

[1] University of Chemistry and Technology in Prague, [1a] Department of Chemistry of Natural Compounds, [1b] Department of Biochemistry and Microbiology, Technická 5, 16628 Prague 6, Czech Republic;

[2] Institute of Experimental Botany of the Czech Academy of Sciences, Isotope Laboratory, Vídeňská 1083, 14220 Prague 4, Czech Republic;

[3] Department of Experimental Biology, Faculty of Science, Palacký University, Šlechtitelů 27, CZ-78371 Olomouc, Czech Republic;

[4] Institute of Organic Chemistry and Biochemistry of the Czech Academy of Sciences, Flemingovo náměstí 2, 16610 Prague 6, Czech Republic;

[5] Ufa Institute of Chemistry of the Russian Academy of Sciences, 71, pr. Oktyabrya, 450054 Ufa, Russian Federation.

Content

1. Synthetic procedures and analytical data 2
2. Antimicrobial screening tests 48
3. Gel formation and supramolecular self-assembly 49

1. Synthetic procedures and analytical data

1.1. Benzyl (3β)-3-hydroxyolean-12-en-28-oate (**2a**)

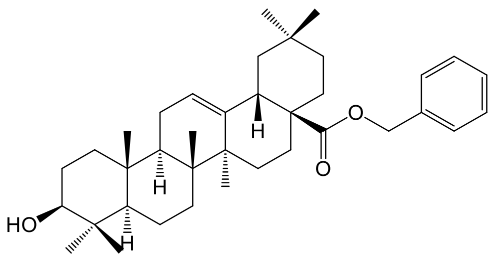

Procedure A was applied, using **1a** as the starting compound, yielding **2a** in a 96 % yield.

^{1}H NMR: δ 0.60 (3H, d, *J*=0.5 Hz, H26), 0.71 (1H, dd, J_1=2.0 Hz, J_2=11.6 Hz, H5), 0.77 (3H, s, H25), 0.87 (3H, d, *J*=0.8 Hz, H24), 0.89 (3H, s, H29), 0.91 (3H, s, H30), 0.97 (3H, s, H23), 1.04 (2H, ddd, J_1=2.7 Hz, J_2=3.9 Hz, J_3=13.8 Hz, H16), 1.12 (3H, d, *J*=0.8 Hz, H27), 1.23 (2H, dt, J_1=3.0 Hz, J_2=3.0 Hz, J_3=12.5 Hz, H7), 1.62-1.67 (2H, m, H11), 1.71 (2H, dt, J_1=4.4 Hz, J_2=13.9 Hz, J_3=13.9 Hz, H22), 1.83-1.87 (2H, m, H2), 1.97 (2H, dt, J_1=4.0 Hz, J_2=13.5 Hz, J_3=13.5 Hz, H11), 2.90 (1H, bdd, J_1=4.7 Hz, J_2=13.8 Hz, H18), 3.20 (1H, dd, J_1=4.4 Hz, J_2=11.5 Hz, H3), 5.04 (2H, d, *J*=12.5 Hz, H1'), 5.09 (2H, d, *J*=12.5 Hz, H1'), 5.28 (1H, t, *J*=3.7 Hz, H12), 7.28-7.37 (1H, m, H4'), 7.28-7.37 (1H, m, H3'), 7.28-7.37 (1H, m, H5').

^{13}C NMR: δ 15.28 (q, C24), 15.56 (q, C25), 16.87 (q, C26), 18.30 (t, C6), 23.04 (t, C11), 23.63 (q, C30), 25.87 (q, C27), 27.18 (t, C15), 27.61 (t, C16), 28.09 (q, C23), 30.69 (s, C20), 32.36 (t, C22), 32.69 (t, C7), 33.09 (q, C29), 33.85 (t, C21), 36.99 (s, C10), 38.42 (s, C4), 38.73 (t, C1), 39.27 (s, C8), 41.36 (d, C18), 41.67 (s, C14), 43.38 (t, C2), 45.86 (t, C19), 46.73 (s, C17), 47.59 (d, C9), 55.19 (d, C5), 65.91 (t, C1'), 79.00 (d, C3), 122.48 (d, C12), 127.89 (d, C5'), 127.96 (d, C3'), 128.39 (d, C4'), 136.42 (s, C2'), 143.68 (s, C13), 177.45 (s, C28).

MS (ES): *m/z* = 547.3 $[M+H]^+$.

1.2. Benzyl (3β)-3-(prop-2-yn-1-yloxy)olean-12-en-28-oate (**3a**)

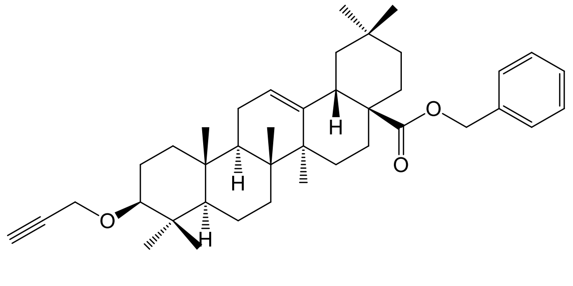

Procedure B was applied, using **2a** as the starting compound, yielding **3a** in an 86 % yield.

^{1}H NMR: δ 0.59 (3H, s, H26), 0.75 (3H, s, H25), 0.86 (3H, s, H24), 0.87 (3H, s, H29), 0.90 (3H, s, H30), 0.97 (3H, s, H23), 1.02 (2H, ddd, J_1=2.7 Hz, J_2=4.2 Hz, J_3=13.8 Hz, H15), 1.10 (3H, d, J=0.8 Hz, H27), 1.53 (2H, ddd, J_1=2.9 Hz, J_2=4.2 Hz, J_3=13.7 Hz, H22), 1.59 (2H, m, J_1=0.8 Hz, J_2=0.8 Hz, J_3=0.8 Hz, J_4=4.3 Hz, J_5=13.8 Hz, J_6=13.8 Hz, H15), 1.71 (1H, dd, J_1=1.9 Hz, J_2=11.8 Hz, H5), 1.79-1.88 (2H, m, H2), 1.96 (2H, dt, J_1=4.0 Hz, J_2=13.6 Hz, J_3=13.6 Hz, H11), 2.34 (1H, t, J=2.4 Hz, H8'), 2.88 (1H, bdd, J_1=4.6 Hz, J_2=13.9 Hz, H18), 3.00 (1H, dd, J_1=4.4 Hz, J_2=11.6 Hz, H3), 4.13 (2H, dd, J_1=2.4 Hz, J_2=16.0 Hz, H6'), 4.20 (2H, dd, J_1=2.4 Hz, J_2=16.0 Hz, H6'), 5.03 (2H, d, J=12.5 Hz, H1'), 5.07 (2H, d, J=12.5 Hz, H1'), 5.27 (1H, t, J=3.7 Hz, H12), 7.26-7.35 (1H, m, H4'), 7.26-7.35 (1H, m, H3'), 7.26-7.35 (1H, m, H5').

^{13}C NMR: δ 15.28 (q, C24), 16.39 (q, C25), 16.88 (q, C26), 18.24 (t, C6), 22.36 (t, C16), 23.04 (t, C11), 23.42 (t, C2), 23.64 (q, C30), 25.86 (q, C27), 27.60 (t, C15), 28.09 (q, C23), 30.69 (s, C20), 32.36 (t, C22), 32.70 (t, C7), 33.10 (q, C29), 33.85 (t, C21), 36.93 (s, C10), 38.28 (t, C1), 38.47 (s, C4), 39.32 (s, C8), 41.37 (d, C18), 41.68 (s, C14), 45.86 (t, C19), 46.73 (s, C17), 47.57 (d, C9), 55.76 (d, C5), 56.42 (t, C6'), 65.91 (t, C1'), 73.41 (d, C8'), 80.96 (s, C7'), 85.88 (d, C3), 122.50 (d, C12), 127.89 (d, C5'), 127.97 (d, C4'), 128.40 (d, C3'), 136.43 (s, C2'), 143.71 (s, C13), 177.43 (s, C28).

MS (ES): m/z = 585.1 $[M+H]^+$; 529,3 $[M-H]^-$

1.3. 3-*O*-Acetyloleanolic acid (**4a**)

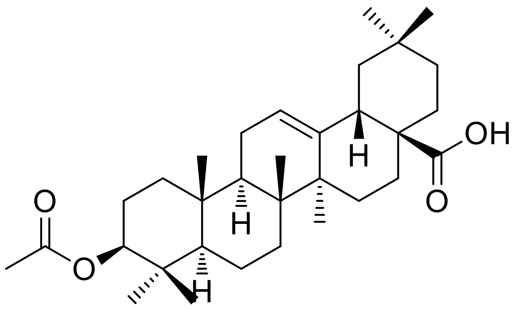

Procedure C was applied, using **1a** as the starting compound, yielding **4a** in a 99 % yield.

^{1}H NMR: δ 0.68 (3H, s, H26), 0.77 (1H, dd, J_1=1.9 Hz, J_2=11.8 Hz, H5), 0.78 (3H, s, H24), 0.80 (3H, s, H23), 0.84 (3H, s, H29), 0.86 (3H, s, H30), 0.87 (3H, s, H25), 1.00 (2H, ddd, J_1=2.7 Hz, J_2=4.0 Hz, J_3=14.1 Hz, H15), 1.06 (3H, d, H27), 1.09 (2H, ddd, J_1=2.3 Hz, J_2=4.7 Hz,

J_3=13.7 Hz, H19), 1.15 (2H, ddt, J_1=2.6 Hz, J_2=2.6 Hz, J_3=4.9 Hz, J_4=13.5 Hz, H21), 1.22 (2H, dt, J_1=3.2 Hz, J_2=3.2 Hz, J_3=12.1 Hz, H7), 1.27 (2H, dt, J_1=4.0 Hz, J_2=14.7 Hz, J_3=13.7 Hz, H21), 1.38 (2H, dt, J_1=3.8 Hz, J_2=12.4 Hz, J_3=12.4 Hz, H7), 1.64 (2H, dt, J_1=4.2 Hz, J_2=14.2 Hz, J_3=14.2 Hz, H15), 1.70 (2H, dt, J_1=4.4 Hz, J_2=13.8 Hz, J_3=13.8 Hz, H22), 1.79 (2H, ddd, J_1=3.5 Hz, J_2=7.1 Hz, J_3=18.6 Hz, H2), 1.84 (2H, ddd, J_1=3.5 Hz, J_2=10.7 Hz, J_3=18.6 Hz, H2), 1.91 (2H, dt, J_1=4.2 Hz, J_2=14.2 Hz, J_3=14.2 Hz, H16), 1.98 (3H, s, H2'), 2.75 (1H, dd, J_1=4.5 Hz, J_2=13.5 Hz, H18), 4.41 (1H, dd, J_1=4.5 Hz, J_2=10.8 Hz, H3), 5.21 (1H, t, J=3.7 Hz, H12).

^{13}C NMR: δ 15.36 (q, C25), 16.64 (q, C24), 17.15 (q, C26), 18.15 (t, C6), 21.30 (q, C2'), 22.85 (t, C16), 23.37 (t, C2), 23.50 (t, C11), 23.56 (q, C30), 25.89 (q, C27), 27.64 (t, C15), 28.02 (q, C23), 30.65 (s, C20), 32.41 (t, C22), 32.49 (t, C7), 33.04 (q, C29), 33.76 (t, C21), 36.96 (s, C4), 37.67 (s, C10), 38.03 (t, C1), 39.25 (s, C8), 40.89 (d, C18), 41.52 (s, C14), 45.80 (t, C19), 46.52 (s, C17), 47.52 (d, C9), 55.26 (d, C5), 80.91 (d, C3), 122.53 (d, C12), 143.58 (s, C13), 171.04 (s, C1'), 183.92 (s, C28).

MS (ES): *m/z* = 516.1 $[M+H]^+$; 497.1 $[M-H]^-$

1.4. (3β)-28-[(2-{2-[2-(2-Azidoethoxy)ethoxy]ethoxy}ethyl)amino]-28-oxoolean-12-en-3-yl acetate (**5a**)

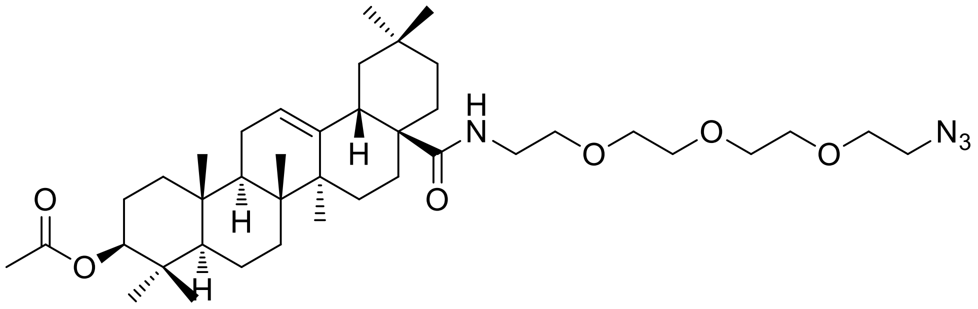

Procedure E was applied, using **4a** as the starting compound, yielding **5a** in a 68 % yield.

^{1}H NMR: δ 0.77 (3H, s, H26), 0.83 (1H, dd, J_1=2.0 Hz, J_2=11.6 Hz, H5), 0.85 (3H, s, H25), 0.86 (3H, s, H23), 0.90 (3H, s, H30), 0.90 (3H, s, H29), 0.93 (3H, s, H24), 1.15 (3H, d, J=0.5 Hz, H27), 1.68 (2H, ddd, J_1=2.9 Hz, J_2=4.1 Hz, J_3=14.1 Hz, H7), 1.75 (2H, t, J=13.3 Hz, H19), 1.86-1.92 (2H, m, H2), 2.04 (2H, s, H10'), 2.54 (1H, bdd, J_1=4.4 Hz, J_2=13.5 Hz, H18), 2.97 (2H, dt, J_1=4.0 Hz, J_2=13.8 Hz, J_3=13.8 Hz, H11), 3.27-3.32 (2H, m, H1'), 3.27-3.40 (2H, m, H8'), 3.49-3.54 (2H, m, H1'), 3.59-3.70 (2H, m, H6'), 3.59-3.70 (2H, m, H7'), 3.59-3.70 (2H,

m, H5'), 3.59-3.70 (2H, m, H3'), 3.59-3.70 (2H, m, H4'), 3.59-3.70 (2H, m, H2'), 4.49 (1H, dd, J_1=5.2 Hz, J_2=10.9 Hz, H3), 5.36 (1H, t, J=3.7 Hz, H12), 6.33 (2H, bt, J=5.1 Hz, H1'-NH).

^{13}C NMR: δ 15.43 (q, C24), 16.64 (q, C25), 16.86 (q, C26), 23.48 (t, C16), 23.53 (t, C11), 23.59 (t, C2), 23.67 (q, C30), 25.68 (q, C27), 27.28 (t, C15), 27.99 (q, C23), 28.15 (t, C6), 30.71 (s, C20), 32.32 (t, C22), 32.55 (t, C7), 32.99 (q, C29), 34.11 (t, C21), 36.82 (s, C10), 37.66 (s, C4), 38.14 (t, C1), 39.04 (t, C1'), 39.38 (s, C8), 41.97 (s, C14), 42.14 (d, C18), 46.34 (s, C17), 46.66 (t, C19), 47.45 (d, C9), 50.65 (t, C8'), 55.16 (d, C5), 69.68 (t, C7'), 70.09 (t, C6'), 70.28 (t, C5'), 70.62 (t, C4'), 70.65 (t, C3'), 70.72 (t, C2'), 80.81 (d, C3), 122.65 (d, C12), 144.62 (s, C13), 178.14 (s, C28).

MS (ES): *m/z* = 699.4 $[M+H]^+$; 697.3 $[M-H]^-$

1.5. Benzyl (3β)-3-{[1-(2-{2-[2-(2-{[(3β)-3-(acetyloxy)-28-oxoolean-12-en-28-yl]amino}-ethoxy)ethoxy]ethoxy}ethyl)-1*H*-1,2,3-triazol-4-yl]methoxy}olean-12-en-28-oate (**6a**)

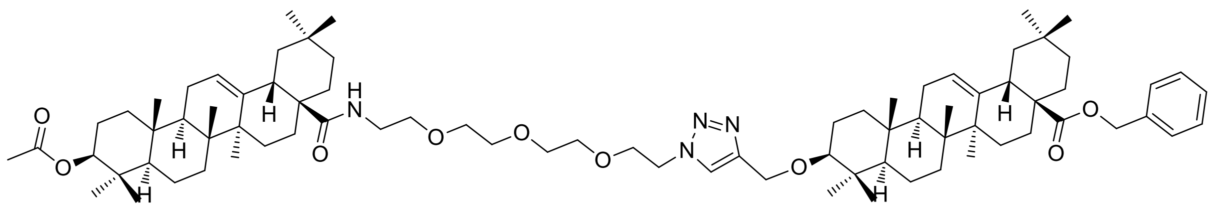

Procedure F was applied, using **4a** and **5a** as the starting compounds, yielding **6a** in a 99 % yield.

^{1}H NMR: δ 0.61 (3H, s, H30), 0.62 (3H, d, J=0.4 Hz, H26), 0.62 (3H, d, J=0.4 Hz, H26''), 0.71 (1H, dd, J_1=2.0 Hz, J_2=11.6 Hz, H5''), 0.78 (3H, s, H25''), 0.85 (1H, dd, J_1=1.9 Hz, J_2=11.5 Hz, H5), 0.86 (3H, s, H25), 0.87 (3H, s, H23), 0.89 (3H, s, H24''), 0.90 (3H, s, H29), 0.91 (3H, s, H23''), 0.91 (3H, s, H29''), 0.92 (3H, s, H30''), 0.94 (3H, d, J=0.7 Hz, H24), 1.12 (3H, d, J=0.6 Hz, H27''), 1.16 (3H, d, J=0.6 Hz, H27), 1.77 (2H, t, J=13.3 Hz, H19), 1.98 (2H, dt, J_1=3.8 Hz, J_2=13.6 Hz, J_3=13.6 Hz, H11), 2.04 (3H, s, OAc), 2.55 (1H, bdd, J_1=4.0 Hz, J_2=13.1 Hz, H18), 2.91 (1H, bdd, J_1=4.5 Hz, J_2=13.6 Hz, H18''), 2.98 (1H, dd, J_1=4.4 Hz, J_2=11.7 Hz, H3''), 3.25-3.31 (2H, m, H1'), 3.50-3.54 (2H, m, H1'), 3.56-3.62 (2H, m, H6'), 3.56-3.62 (2H, m, H5'), 3.56-3.62 (2H, m, H4'), 3.56-3.62 (2H, m, H2'), 3.56-3.62 (2H, m, H3'), 3.86-3.89 (2H, m, H7'), 4.50-4.54 (2H, m, H8'), 4.56 (1H, dd, J_1=5.4 Hz, J_2=11.2 Hz, H3), 4.56 (2H, dd, J_1=0.6 Hz, J_2=12.4

Hz, H11'), 4.76 (2H, dd, J_1=0.7 Hz, J_2=12.4 Hz, H11'), 5.05 (2H, d, *J*=12.5 Hz, H1'''), 5.09 (2H, d, *J*=12.5 Hz, H1'''), 5.28 (1H, t, *J*=3.9 Hz, H12"), 5.35 (1H, t, *J*=3.6 Hz, H12), 7.28-7.36 (2H, m, H5'''), 7.28-7.36 (1H, m, H3'''), 7.28-7.36 (1H, m, H4'''), 7.65 (1H, s, H9').

^{13}C NMR: δ 15.34 (q, C24"), 15.48 (q, C24), 16.52 (q, C25"), 16.98 (q, C26), 16.99 (q, C26"), 18.25 (t, C6), 18.34 (t, C6"), 21.22 (q, OAc), 22.80 (t, C16"), 23.16 (t, C16), 23.49 (t, C11), 23.58 (t, C11"), 23.62 (t, C2), 23.67 (q, C30), 23.67 (t, C2"), 23.81 (q, C30"), 24.74 (q, C27), 25.90 (q, C27"), 27.41 (t, C15), 27.71 (t, C15"), 28.07 (q, C23), 28.22 (q, C23"), 30.71 (s, C20), 30.74 (s, C20"), 32.46 (t, C22), 32.49 (t, C22"), 32.75 (t, C7), 32.84 (t, C7"), 33.01 (q, C29), 33.10 (q, C29"), 33.96 (t, C21"), 34.23 (t, C21), 36.96 (s, C10), 37.05 (s, C10"), 37.75 (s, C4), 38.30 (t, C1), 38.41 (t, C1"), 38.78 (s, C4"), 39.15 (t, C1'), 39.46 (s, C8), 39.52 (s, C8"), 41.50 (d, C18"), 41.78 (s, C14"), 42.11 (s, C14), 42.30 (d, C18), 45.98 (s, C17"), 46.44 (s, C17), 46.69 (q, C25), 46.78 (t, C19), 46.83 (t, C19"), 47.58 (d, C9), 47.68 (d, C9"), 50.38 (t, C8'), 55.32 (d, C5), 55.73 (d, C5"), 63.17 (t, C11'), 65.93 (t, C1'''), 69.61 (t, C7'), 69.82 (t, C6'), 70.31 (t, C5'), 70.56 (t, C4'), 70.67 (t, C3'), 70.69 (t, C2'), 80.86 (d, C3), 86.63 (d, C3"), 122.59 (d, C12"), 122.66 (d, C12), 123.37 (d, C9'), 123.54 (s, C2'''), 127.89 (d, C5'''), 128.00 (d, C3'''), 128.40 (d, C4'''), 143.74 (s, C13"), 144.79 (s, C13), 146.31 (s, C10'), 170.87 (s, OAc), 177.40 (s, C28"), 178.05 (s, C28).

MS (ES): *m/z* = 1283.9 $[M+H]^+$; 1282.4 $[M-H]^-$

1.6. (3β)-3-{[1-(2-{2-[2-(2-{[(3β)-3-(Acetyloxy)-28-oxoolean-12-en-28-yl]amino}ethoxy)-ethoxy]ethoxy}ethyl)-1*H*-1,2,3-triazol-4-yl]methoxy}olean-12-en-28-oic acid (**7a**)

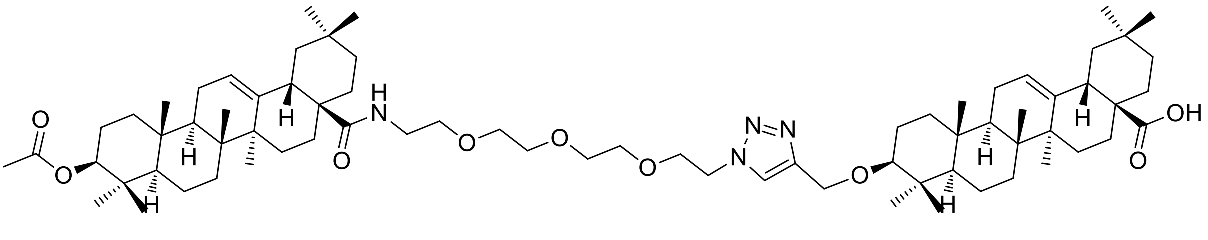

Procedure G was applied, using **6a** as the starting compound, yielding **7a** in a 91 % yield.

^{1}H NMR: δ 0.72 (1H, dd, J_1=1.8 Hz, J_2=11.4 Hz, H5"), 0.74 (3H, s, H26"), 0.76 (3H, s, H26), 0.77 (3H, s, H25), 0.83 (1H, dd, J_1=1.8 Hz, J_2=11.6 Hz, H5), 0.86 (3H, s, H25"), 0.86 (3H, s,

H23"), 0.89 (3H, s, H30), 0.89 (3H, s, H23), 0.90 (3H, s, H29"), 0.90 (3H, s, H29), 0.90 (3H, s, H30"), 0.91 (3H, s, H24"), 0.92 (3H, dd, *J*=0.5 Hz, H24), 1.12 (3H, s, H27"), 1.15 (3H, s, H27), 1.97 (2H, tt, J_1=3.8 Hz, J_2=3.8 Hz, J_3=11.8 Hz, J_4=11.8 Hz, H11), 2.05 (3H, s, OAc), 2.53 (1H, bdd, J_1=4.1 Hz, J_2=13.0 Hz, H18), 2.82 (1H, bdd, J_1=4.7 Hz, J_2=13.8 Hz, H18"), 2.98 (1H, dd, J_1=4.7 Hz, J_2=13.8 Hz, H3"), 3.25-3.31 (2H, m, H1'), 3.49-3.54 (2H, m, H1'), 3.57-3.62 (2H, m, H6'), 3.57-3.62 (2H, m, H3'), 3.57-3.62 (2H, m, H4'), 3.57-3.62 (2H, m, H2'), 3.57-3.62 (2H, m, H5'), 3.86-3.89 (2H, m, H7'), 4.49 (1H, dd, J_1=5.1 Hz, J_2=11.0 Hz, H3), 4.52-4.55 (2H, m, H8'), 4.57 (2H, dd, J_1=0.5 Hz, J_2=12.5 Hz, H11'), 4.77 (2H, dd, J_1=0.5 Hz, J_2=12.5 Hz, H11'), 5.28 (1H, tt, *J*=3.8 Hz, H12"), 5.35 (1H, tt, *J*=3.7 Hz, H12), 7.69 (1H, s, H9').

^{13}C NMR: δ 12.61 (q, C30), 15.33 (q, C24"), 15.44 (q, C24), 16.48 (q, C25"), 16.66 (q, C25), 17.06 (q, C26"), 18.15 (d, C6), 18.23 (t, C6"), 22.67 (d, C16), 22.67 (q, C26), 22.92 (t, C16"), 23.40 (t, C11"), 23.47 (s, C11), 23.54 (t, C2"), 23.56 (q, C30"), 23.69 (t, C2), 25.69 (q, C27), 25.90 (q, C27"), 27.28 (t, C15"), 27.64 (t, C15), 27.99 (q, C23"), 28.12 (q, C23), 30.66 (s, C20"), 30.71 (s, C20), 32.31 (t, C7), 32.41 (t, C7"), 32.57 (t, C22"), 32.60 (t, C22), 32.99 (q, C29), 33.05 (q, C29"), 33.78 (t, C21"), 34.10 (t, C21), 36.82 (s, C10"), 36.99 (d, C10), 37.65 (s, C4), 38.16 (t, C1"), 38.19 (t, C1), 38.68 (s, C4"), 39.02 (t, C1'), 39.27 (s, C8"), 39.38 (t, C8), 41.01 (d, C18"), 41.59 (s, C14"), 41.98 (s, C14), 42.16 (d, C18), 45.85 (t, C19), 46.34 (s, C17"), 46.45 (t, C19"), 46.65 (s, C17), 47.44 (d, C9"), 48.55 (s, C9), 50.43 (t, C8'), 55.15 (d, C5), 55.56 (d, C5"), 62.90 (t, C11'), 69.48 (t, C7'), 69.71 (t, C6'), 70.22 (t, C5'), 70.46 (t, C4'), 70.57 (t, C2'), 70.57 (t, C3'), 80.83 (d, C3), 86.50 (d, C3"), 122.56 (d, C12"), 122.57 (t, C12), 123.65 (d, C9'), 143.59 (s, C13"), 144.69 (d, C13), 145.99 (s, C10'), 178.14 (s, C28), 182.29 (s, C28").

MS (ES): *m/z* = 1215.6 [M+H]$^+$; 1192.0 [M-H]$^-$

1.7. (3β)-3-{[1-(2-{2-[2-(2-{[(3β)-3-Hydroxy-28-oxoolean-12-en-28-yl]amino}ethoxy)-ethoxy]ethoxy}ethyl)-1*H*-1,2,3-triazol-4-yl]methoxy}olean-12-en-28-oic acid (**8a**)

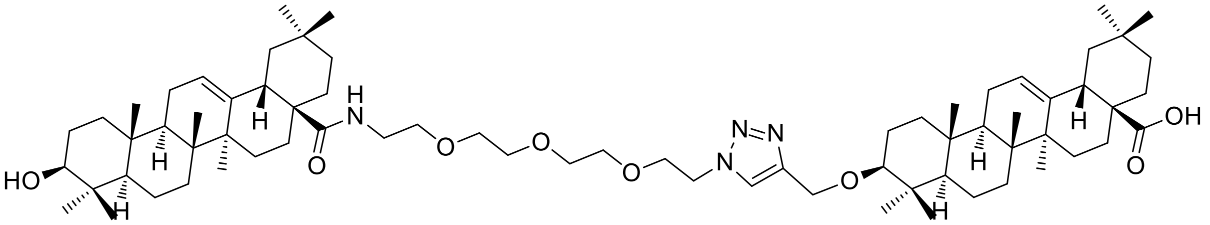

Procedure H was applied, using **7a** as the starting compound, yielding **8a** in a 95 % yield.

^{1}H NMR: δ 0.73 (1H, dd, J_1=1.8 Hz, J_2=11.7 Hz, H5"), 0.73 (1H, dd, J_1=1.8 Hz, J_2=11.7 Hz, H5), 0.74 (3H, s, H26"), 0.76 (3H, s, H25"), 0.77 (3H, s, H25), 0.77 (3H, s, H26), 0.89 (3H, s, H30), 0.89 (3H, s, H29"), 0.89 (3H, s, H30"), 0.90 (3H, s, H29), 0.91 (3H, s, H24"), 0.92 (3H, s, *J*=0.5 Hz, H24), 0.98 (3H, s, H23), 0.98 (3H, s, H23"), 1.12 (3H, s, H27"), 1.15 (3H, s, H27), 1.97 (2H, dt, J_1=3.8 Hz, J_2=13.6 Hz, J_3=13.6 Hz, H11"), 1.97 (2H, dt, J_1=3.8 Hz, J_2=23.6 Hz, J_3=13.6 Hz, J_4=11.8 Hz, H11), 2.53 (1H, bdd, J_1=4.7 Hz, J_2=13.6 Hz, H18), 2.82 (1H, bdd, J_1=4.7 Hz, J_2=12.2 Hz, H18"), 2.98 (1H, dd, J_1=4.2 Hz, J_2=11.7 Hz, H3"), 3.22 (1H, dd, J_1=4.6 Hz, J_2=11.5 Hz, H3), 3.28-3.32 (2H, m, H1'), 3.48-3.51 (2H, m, H1'), 3.48-3.62 (2H, m, H5'), 3.48-3.62 (2H, m, H2'), 3.48-3.62 (2H, m, H6'), 3.48-3.62 (2H, m, H4'), 3.48-3.62 (2H, m, H3'), 3.87 (2H, t, *J*=5.3 Hz, H7'), 4.54 (2H, t, *J*=5.3 Hz, H8'), 4.57 (2H, d, J_1=12.5 Hz, J_2=12.5 Hz, H11'), 4.77 (2H, d, J_1=12.5 Hz, J_2=12.5 Hz, H11'), 5.28 (1H, t, J_1=3.8 Hz, J_2=3.8 Hz, H12"), 5.35 (1H, t, *J*=3.8 Hz, H12), 7.69 (1H, s, H9').

^{13}C NMR: δ 14.45 (q, C25"), 15.34 (q, C24"), 15.39 (q, C24), 15.59 (q, C26), 16.87 (q, C25), 17.11 (q, C26"), 18.23 (t, C6), 18.27 (t, C6"), 22.68 (t, C16), 22.93 (t, C16"), 23.40 (t, C11"), 23.54 (t, C11), 23.57 (t, C2"), 23.62 (q, C30"), 23.69 (q, C30), 25.74 (q, C27), 25.90 (q, C27"), 27.08 (t, C2), 27.30 (t, C15"), 27.65 (t, C15), 28.07 (q, C23"), 28.14 (q, C23), 30.66 (s, C20"), 30.72 (s, C20), 32.37 (t, C7), 32.41 (t, C7"), 32.57 (t, C22"), 32.66 (t, C22), 33.00 (q, C29), 33.06 (q, C29"), 33.78 (t, C21"), 34.11 (t, C21), 36.93 (s, C10"), 36.99 (s, C10), 38.18 (s, C4), 38.48 (t, C1), 38.69 (t, C1"), 38.73 (s, C4"), 39.03 (t, C1'), 39.28 (s, C8"), 39.37 (s, C8), 41.04 (d, C18"), 41.60 (s, C14"), 42.00 (s, C14), 42.16 (d, C18), 45.84 (t, C19), 46.35 (s, C17"), 46.44 (t, C19"), 46.71 (s, C17), 47.50 (d, C9"), 47.54 (d, C9), 50.55 (t, C8'), 55.06 (d, C5), 55.55 (d, C5"), 62.82 (t, C11'), 69.43 (t, C7'), 69.72 (t, C6'), 70.21 (t, C5'), 70.45 (t, C4'), 70.57 (t, C2'), 70.57 (t, C3'), 78.95 (d, C3), 86.59 (d, C3"), 122.58 (d, C12), 122.68 (d, C12"), 123.82 (d, C9'), 143.59 (s, C13"), 144.70 (s, C13), 145.89 (s, C10'), 178.17 (s, C28), 182.02 (s, C28").

MS (ES): *m/z* = 1151.6 $[M+H]^+$; 1149.9 $[M-H]^-$

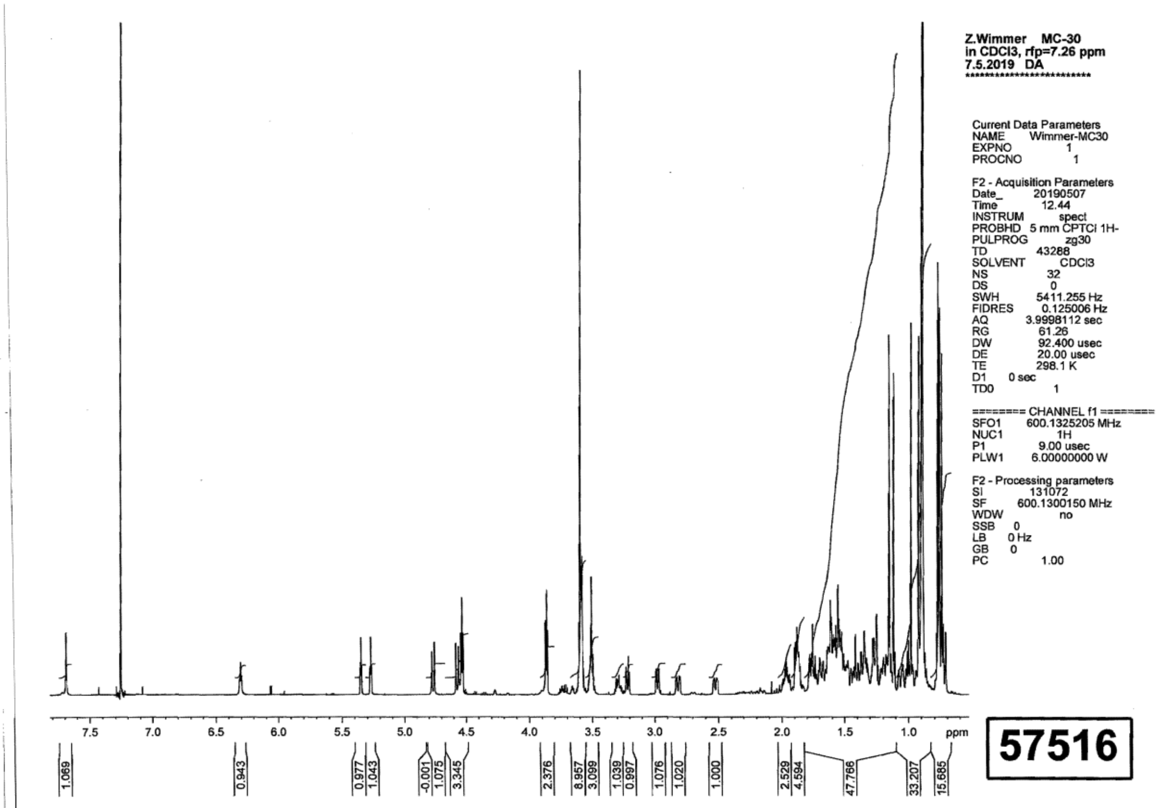
Z.Wimmer MC-30
in CDCl3, rfp=7.26 ppm
7.5.2019 DA
Current Data Parameters
NAME Wimmer-MC30
EXPNO 1
PROCNO 1
F2 - Acquisition Parameters
Date_ 20190507
Time 12.44
INSTRUM spect
PROBHD 5 mm CPTCI 1H-
PULPROG zg30
TD 43288
SOLVENT CDCl3
NS 32
DS 0
SWH 5411.255 Hz
FIDRES 0.125006 Hz
AQ 3.9998112 sec
RG 61.26
DW 92.400 usec
DE 20.00 usec
TE 298.1 K
D1 0 sec
TD0 1
======== CHANNEL f1 ========
SFO1 600.1325205 MHz
NUC1 1H
P1 9.00 usec
PLW1 6.00000000 W
F2 - Processing parameters
SI 131072
SF 600.1300150 MHz
WDW no
SSB 0
LB 0 Hz
GB 0
PC 1.00

57516

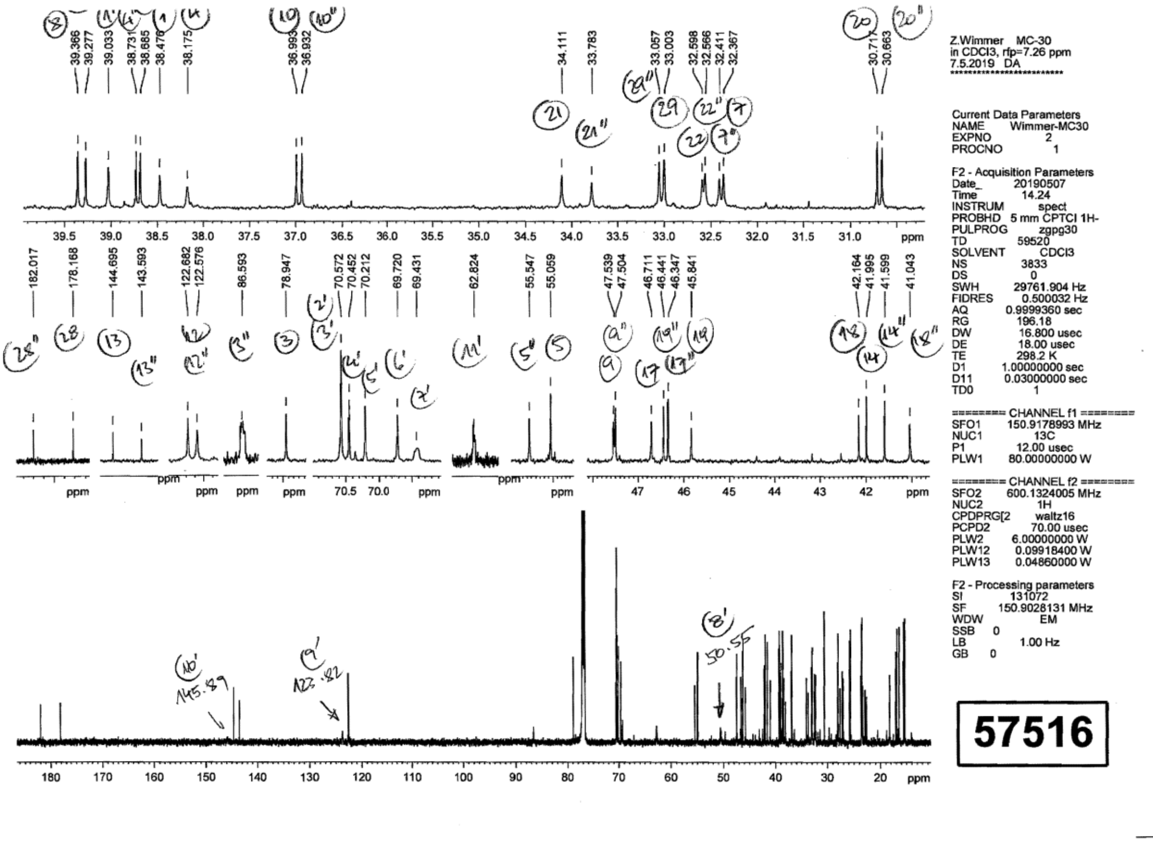
Z.Wimmer MC-30
in CDCl3, rfp=7.26 ppm
7.5.2019 DA
Current Data Parameters
NAME Wimmer-MC30
EXPNO 2
PROCNO 1
F2 - Acquisition Parameters
Date_ 20190507
Time 14.24
INSTRUM spect
PROBHD 5 mm CPTCI 1H-
PULPROG zgpg30
TD 59520
SOLVENT CDCl3
NS 3833
DS 0
SWH 29761.904 Hz
FIDRES 0.500032 Hz
AQ 0.9999360 sec
RG 196.18
DW 16.800 usec
DE 18.00 usec
TE 298.2 K
D1 1.00000000 sec
D11 0.03000000 sec
TD0 1
======== CHANNEL f1 ========
SFO1 150.9178993 MHz
NUC1 13C
P1 12.00 usec
PLW1 80.00000000 W
======== CHANNEL f2 ========
SFO2 600.1324005 MHz
NUC2 1H
CPDPRG[2 waltz16
PCPD2 70.00 usec
PLW2 6.00000000 W
PLW12 0.09918400 W
PLW13 0.04860000 W
F2 - Processing parameters
SI 131072
SF 150.9028131 MHz
WDW EM
SSB 0
LB 1.00 Hz
GB 0

57516

1.8. (3β)-28-[(2-{2-[2-(2-{4-[({(3β)-28-[(2-{2-[2-(2-Azidoethoxy)ethoxy]ethoxy}ethyl)-amino]-28-oxoolean-12-en-3-yl}oxy)methyl]-1*H*-1,2,3-triazol-1-yl}ethoxy)ethoxy]ethoxy}-ethyl)amino]-28-oxoolean-12-en-3-yl acetate (**9a**)

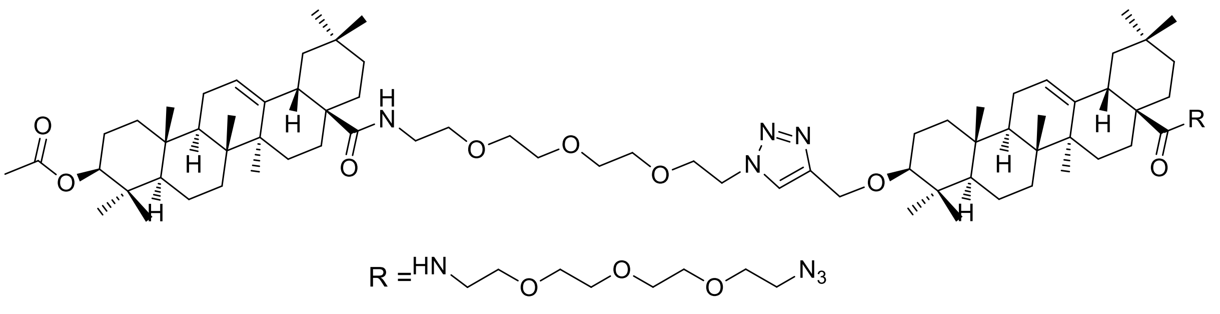

Procedure E was applied, using **8a** as the starting compound, yielding **9a** in a 90 % yield.

^{1}H NMR: δ 0.72 (1H, dd, J_1=1.9 Hz, J_2=11.6 Hz, H5''), 0.76 (3H, s, H26), 0.76 (3H, s, H26''), 0.78 (3H, s, H25), 0.83 (1H, dd, J_1=1.8 Hz, J_2=11.7 Hz, H5), 0.85 (3H, s, H25''), 0.86 (3H, s, H23), 0.89 (3H, s, H23''), 0.89 (3H, s, H30), 0.89 (3H, s, H30''), 0.90 (3H, s, H24''), 0.90 (3H, s, H29), 0.91 (3H, s, H29''), 0.92 (3H, d, J=0.5 Hz, H24), 1.12 (3H, d, J=0.6 Hz, H27), 1.14 (3H, d, J=0.6 Hz, H27''), 1.68 (2H, ddt, J_1=2.8 Hz, J_2=2.8 Hz, J_3=4.1 Hz, J_4=14.3 Hz, H22), 1.76 (2H, t, J=13.3 Hz, H19), 1.97 (2H, tt, J_1=3.8 Hz, J_2=3.8 Hz, J_3=13.8 Hz, J_4=13.8 Hz, H11), 2.53 (1H, bdd, J_1=34.0 Hz, J_2=13.3 Hz, H18), 2.53 (1H, bdd, J_1=4.0 Hz, J_2=13.3 Hz, H18''), 2.99 (1H, dd, J_1=4.3 Hz, J_2=11.7 Hz, H3''), 3.25-3.32 (2H, m, H1*), 3.26-3.32 (2H, m, H1'), 3.37-3.40 (2H, m, H8*), 3.48-3.54 (2H, m, H1'), 3.48-3.54 (2H, m, H1*), 3.57-3.69 (2H, m, H3*), 3.57-3.69 (2H, m, H6'), 3.57-3.69 (2H, m, H4'), 3.57-3.69 (2H, m, H4*), 3.57-3.69 (2H, m, H3'), 3.57-3.69 (2H, m, H2*), 3.57-3.69 (2H, m, H2'), 3.57-3.69 (2H, m, H5'), 3.57-3.69 (2H, m, H6*), 3.57-3.69 (2H, m, H5*), 3.85-3.89 (2H, m, H7'), 3.85-3.89 (2H, m, H7*), 4.49 (1H, dd, J_1=5.1 Hz, J_2=10.6 Hz, H3), 4.52-4.55 (2H, m, H8'), 4.56 (2H, dd, J_1=0.6 Hz, J_2=12.5 Hz, H11'), 4.77 (2H, dd, J_1=0.6 Hz, J_2=12.5 Hz, H11'), 5.35 (1H, t, J=3.6 Hz, H12), 5.36 (1H, t, J=3.7 Hz, H12''), 6.29 (1H, t, J=0.0 Hz, H1*-NH), 6.32 (1H, t, J=5.5 Hz, H1'-NH), 7.68 (1H, s, H9').

^{13}C NMR: δ 15.41 (q, C24''), 15.45 (q, C24), 16.47 (q, C25''), 16.66 (q, C25), 16.89 (q, C26), 16.89 (q, C26''), 18.15 (t, C6), 18.22 (t, C6''), 22.61 (t, C16''), 22.61 (t, C16), 23.48 (t, C11''), 23.54 (t, C11), 23.57 (t, C2''), 23.60 (q, C30), 23.69 (t, C2), 25.69 (q, C27''), 25.73 (q, C27), 27.28 (t, C15''), 27.30 (t, C15), 28.00 (q, C23''), 28.09 (q, C23), 30.72 (s, C20''), 30.72 (s, C20),

32.31 (t, C7), 32.31 (t, C7''), 32.59 (t, C22''), 32.59 (t, C22), 32.61 (q, C30''), 32.99 (q, C29''), 33.00 (q, C29), 34.11 (t, C21''), 34.13 (t, C21), 36.83 (s, C10''), 36.89 (s, C10), 37.66 (s, C4), 38.15 (t, C1''), 38.28 (t, C1), 38.67 (s, C4''), 39.03 (t, C1*), 39.03 (t, C1'), 39.38 (s, C8''), 39.41 (s, C8), 41.98 (s, C14), 41.98 (s, C14''), 42.15 (d, C18), 42.15 (d, C18''), 46.34 (t, C19''), 46.34 (t, C19), 46.66 (s, C17''), 46.69 (s, C17), 47.44 (d, C9''), 47.48 (d, C9), 50.66 (t, C8*), 50.66 (t, C8'), 55.16 (d, C5), 55.46 (d, C5''), 62.88 (t, C11'), 69.48 (t, C7'), 69.69 (t, C7*), 69.73 (t, C6'), 70.22 (t, C5'), 70.29 (t, C6*), 70.46 (t, C4'), 70.58 (t, C3'), 70.58 (t, C2'), 70.63 (t, C5*), 70.66 (t, C4*), 70.69 (t, C3*), 70.73 (t, C2*), 80.77 (d, C3), 86.40 (d, C3''), 122.59 (d, C12''), 122.74 (d, C12), 123.65 (d, C9'), 125.96 (s, C10'), 144.62 (s, C13''), 144.71 (s, C13), 178.10 (s, C28''), 178.13 (s, C28).

MS (ES): *m/z* = 1394.3 $[M+H]^+$; 1392.2 $[M-H]^-$

1.9. Benzyl (3β)-3-{[1-(2-{2-[2-(2-{[(3β)-3-{[1-(2-{2-[2-(2-{[(3β)-3-(acetyloxy)-28-oxoolean-12-en-28-yl]amino}ethoxy)ethoxy]ethoxy}ethyl)-1*H*-1,2,3-triazol-4-yl]methoxy}-28-oxoolean-12-en-28-yl]amino}ethoxy)ethoxy]ethoxy}ethyl)-1*H*-1,2,3-triazol-4-yl]methoxy}olean-12-en-28-oate (**10a**)

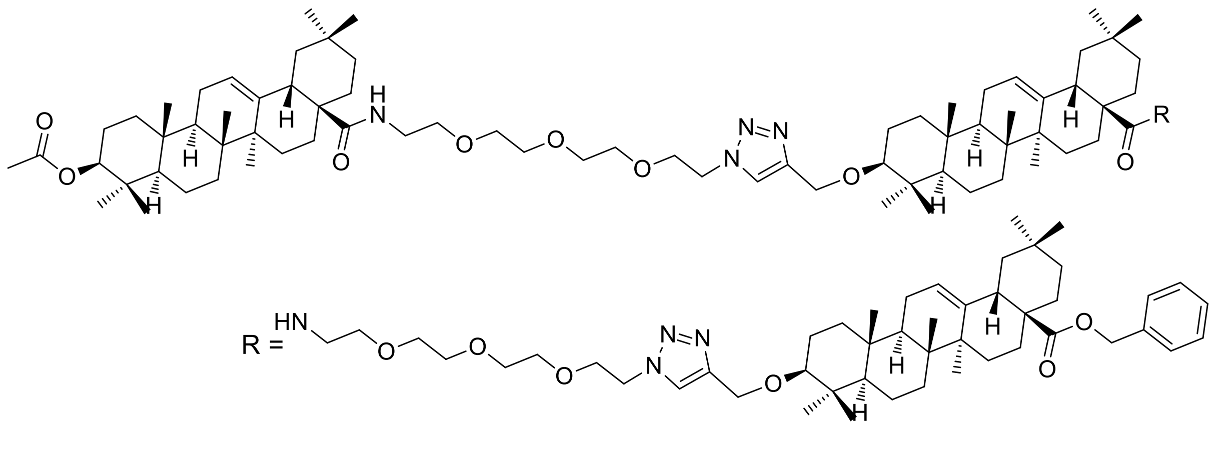

Procedure F was applied, using **9a** and **3a** as the starting compounds, yielding **10a** in a 97 % yield.

^{1}H NMR: δ 0.59 (s, H24), 0.70 (dd, J_1=1.7 Hz, J_2=10.7 Hz, H5), 0.72 (dd, J_1=1.8 Hz, J_2=11.4 Hz, H5*), 0.75 (s, H26), 0.76 (s, H26''), 0.77 (s, H25*), 0.77 (s, H25''), 0.77 (s, H26*), 0.83 (dd, J_1=1.8 Hz, J_2=11.6 Hz, H5''), 0.84 (s, H29), 0.84 (s, H23), 0.86 (s, H25), 0.87 (s, H30), 0.88 (s, H29''), 0.90 (s, H24''), 0.90 (s, H24*), 0.90 (s, H30''), 0.90 (s, H29*), 0.91 (s, H30*), 0.91 (s,

H23''), 0.92 (s, H23*), 1.11 (s, H27), 1.14 (s, H27''), 1.15 (s, H27*), 2.04 (s, OAc), 2.53 (bdd, J_1=4.2 Hz, J_2=14.0 Hz, H18''), 2.53 (bdd, J_1=4.2 Hz, J_2=14.0 Hz, H18*), 2.70 (dt, J_1=3.3 Hz, J_2=3.3 Hz, J_3=13.6 Hz, H15*), 2.89 (bdd, J_1=5.0 Hz, J_2=14.1 Hz, H18), 2.97 (dd, J_1=4.4 Hz, J_2=10.7 Hz, H3''), 2.99 (dd, J_1=4.2 Hz, J_2=10.7 Hz, H3*), 4.50 (dd, J_1=4.5 Hz, J_2=10.5 Hz, H3), 5.04 (d, *J*=12.5 Hz, HCH2-HPh), 5.08 (d, *J*=12.5 Hz, HCH2-HPh), 5.28 (t, *J*=3.8 Hz, H12*), 5.35 (t, *J*=3.5 Hz, H12), 5.35 (t, *J*=3.7 Hz, H12''), 7.26-7.40 (m, HCH2-HPh), 7.72 (s, H9'), 7.73 (s, H9''').

^{13}C NMR: δ 15.30 (C24*), 15.40 (C25), 15.50 (C24''), 16.50 (C25''), 16.50 (C25*), 16.70 (C24), 16.90 (C26), 16.90 (C26*), 16.90 (C26''), 18.20 (C6), 18.20 (C6''), 18.20 (C6*), 20.60 (OAc), 22.60 (C16*), 22.70 (C16''), 23.00 (C11*), 23.40 (C11''), 23.40 (C2*), 23.50 (C16), 23.50 (C2''), 23.60 (C2), 23.60 (C30*), 23.60 (C11), 23.60 (C30''), 23.70 (C30), 25.70 (C27), 25.70 (C27''), 25.90 (C27*), 28.00 (C15*), 28.00 (C23''), 28.00 (C15''), 28.10 (C15), 28.10 (C23), 28.40 (C23*), 30.70 (C20''), 30.70 (C20), 30.70 (C20*), 32.30 (C22''), 32.40 (C22*), 32.60 (C7''), 32.60 (C7), 32.70 (C7*), 33.00 (C22), 33.10 (C29), 33.10 (C29''), 33.20 (C29*), 33.80 (C21*), 33.90 (C21''), 34.10 (C21), 36.80 (C10), 36.80 (C14), 36.90 (C4), 36.90 (C10*), 36.90 (C10''), 38.10 (C1*), 38.20 (C1''), 38.30 (C1), 38.70 (C4''), 38.70 (C4*), 39.20 (C8''), 39.30 (C8*), 39.40 (C12), 39.40 (C8), 39.40 (C1'''), 39.80 (C1'), 41.40 (C18*), 41.70 (C14''), 41.70 (C18''), 42.00 (C18), 42.00 (C14*), 45.80 (C19*), 46.30 (C19), 46.30 (C17*), 46.30 (C17''), 46.30 (C19''), 46.70 (C17), 47.40 (C13), 47.40 (C9), 47.40 (C9''), 47.50 (C9*), 52.00 (C8'''), 52.40 (C8'), 55.20 (C5*), 55.40 (C5), 55.50 (C5''), 62.70 (C11'), 62.70 (C11'''), 65.90 (CCH2-Ph), 69.20 (C7'''), 69.20 (C7'), 69.70 (C6'), 69.70 (C6'''), 70.20 (C5'), 70.20 (C5'''), 70.40 (C4'), 70.40 (C4'''), 70.50 (C3'''), 70.50 (C3'), 70.60 (C2'''), 70.60 (C2'), 80.80 (C3), 86.80 (C3''), 86.90 (C3*), 122.60 (C12*), 122.70 (C12''), 124.60 (C9'), 124.80 (C9'''), 127.90 (CCH2-Ph), 127.90 (CCH2-Ph), 128.40 (CCH2-Ph), 136.40 (CCH2-Ph), 143.70 (C13*), 144.70 (C13''), 144.70 (C10'''), 144.70 (C10'), 171.00 (OAc), 177.50 (C28), 178.20 (C28*), 178.20 (C28'').

MS (ES): *m/z* = 1985.4 $[M-H]^-$

1.10. (3β)-3-{[1-(2-{2-[2-(2-{[(3β)-3-{[1-(2-{2-[2-(2-{[(3β)-3-(Acetyloxy)-28-oxoolean-12-en-28-yl]amino}ethoxy)ethoxy]ethoxy}ethyl)-1*H*-1,2,3-triazol-4-yl]methoxy}-28-oxoolean-12-en-28-yl]amino}ethoxy)ethoxy]ethoxy}ethyl)-1*H*-1,2,3-triazol-4-yl]methoxy}olean-12-en-28-oic acid (**11a**)

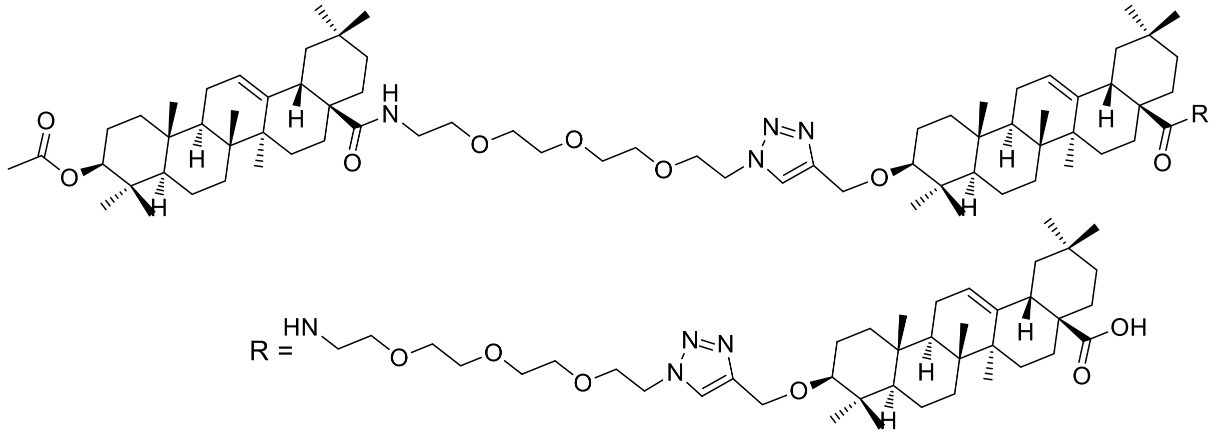

Procedure G was applied, using **10a** as the starting compound, yielding **11a** in a 99 % yield.

^{1}H NMR: δ 0.72 (dd, J_1=1.8 Hz, J_2=11.4 Hz, H5*), 0.75 (s, H26"), 0.75 (s, H26), 0.76 (s, H26*), 0.76 (dd, J_1=1.7 Hz, J_2=10.7 Hz, H5), 0.77 (s, H24), 0.78 (s, H25*), 0.78 (s, H25"), 0.79 (s, H23), 0.83 (dd, J_1=1.8 Hz, J_2=11.6 Hz, H5"), 0.85 (s, H29), 0.86 (s, H25), 0.87 (s, H30), 0.89 (s, H29"), 0.90 (s, H29*), 0.90 (s, H24"), 0.90 (s, H30"), 0.91 (s, H23"), 0.91 (s, H30*), 0.91 (s, H24*), 0.92 (s, H23*), 1.10 (s, H27), 1.15 (s, H27*), 1.15 (s, H27"), 2.04 (s, OAc), 2.50 (bdd, J_1=4.2 Hz, J_2=14.0 Hz, H18"), 2.53 (bdd, J_1=4.2 Hz, J_2=14.0 Hz, J_3=13.6 Hz, H18*), 2.84 (bdd, J_1=5.0 Hz, J_2=14.1 Hz, H18), 2.97 (dd, J_1=4.4 Hz, J_2=10.7 Hz, H3"), 2.99 (dd, J_1=4.2 Hz, J_2=10.7 Hz, H3*), 4.48 (dd, J_1=4.5 Hz, J_2=10.5 Hz, H3), 5.28 (t, J=3.8 Hz, H12*), 5.34 (t, J=3.7 Hz, H12"), 5.35 (t, J=3.5 Hz, H12), 7.76 (s, H9'), 7.78 (s, H9''').

^{13}C NMR: δ 15.40 (C25), 15.40 (C24*), 15.50 (C24"), 16.50 (C25*), 16.50 (C25"), 16.70 (C24), 16.90 (C26), 16.90 (C26"), 17.10 (C26*), 18.20 (C6*), 18.20 (C6"), 18.30 (C6), 20.60 (OAc), 22.50 (C16*), 22.70 (C16"), 23.00 (C11*), 23.40 (C2*), 23.40 (C11"), 23.50 (C16), 23.50 (C2"), 23.60 (C30"), 23.60 (C11), 23.60 (C2), 23.60 (C30*), 23.70 (C30), 25.70 (C27), 25.90 (C27*), 26.30 (C27"), 28.00 (C15), 28.00 (C15*), 28.00 (C15"), 28.10 (C23"), 28.20 (C23*), 28.20 (C23), 30.70 (C20), 30.70 (C20*), 30.90 (C20"), 32.30 (C22"), 32.40 (C22*), 32.60 (C7"), 32.60 (C7), 32.60 (C7*), 33.00 (C22), 33.00 (C29), 33.10 (C29*), 33.20 (C29"), 33.90 (C21*), 33.90 (C21"), 34.10 (C21), 36.80 (C10), 36.80 (C10"), 36.90 (C4), 37.00 (C10*), 38.10 (C1*), 38.20 (C1), 38.20 (C1"), 38.70 (C4*), 38.80 (C4"), 39.10 (C8"), 39.30 (C8*), 39.40 (C8), 39.40 (C1'''), 39.40 (C1'), 41.10 (C18*), 41.70 (C14"), 41.70 (C14*), 41.70 (C18"), 42.00 (C18), 42.00 (C14), 46.00 (C19*), 46.30 (C19), 46.30 (C17"), 46.30 (C19"), 46.40 (C17*), 46.60 (C17), 47.40 (C9), 47.40 (C9"), 47.50 (C9*), 51.50 (C8'''), 52.40 (C8'), 54.80 (C5), 55.50 (C5*), 55.50 (C5"), 61.90 (C11'''), 62.10 (C11'), 69.00 (C7'''), 69.10 (C7'), 70.20 (C6'), 70.30

(C6'''), 70.30 (C5'), 70.40 (C4'''), 70.40 (C4'), 70.40 (C5'''), 70.60 (C3'), 70.60 (C3'''), 70.70 (C2'), 70.70 (C2'''), 80.80 (C3), 86.90 (C3''), 87.20 (C3*), 122.30 (C12), 122.60 (C12*), 122.70 (C12''), 124.70 (C9'), 124.80 (C9'''), 143.80 (C13*), 144.70 (C13), 144.70 (C13''), 144.90 (C10'''), 144.90 (C10'), 171.00 (OAc), 178.20 (C28), 180.70 (C28*), 181.70 (C28'').

MS (ES): *m/z* = 1889.3 $[M+H]^+$

1.11. (3β)-3-{[1-(2-{2-[2-(2-{[(3β)-3-{[1-(2-{2-[2-(2-{[(3β)-3-Hydroxy-28-oxoolean-12-en-28-yl]amino}ethoxy)ethoxy]ethoxy}ethyl)-1*H*-1,2,3-triazol-4-yl]methoxy}-28-oxoolean-12-en-28-yl]amino}ethoxy)ethoxy]ethoxy}ethyl)-1*H*-1,2,3-triazol-4-yl]methoxy}olean-12-en-28-oic acid (**12a**)

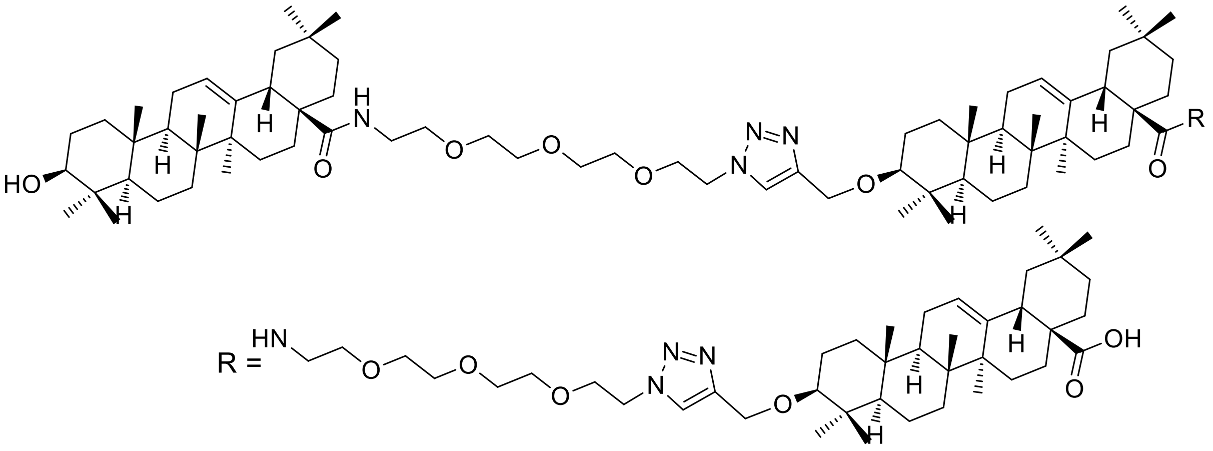

Procedure H was applied, using **11a** as the starting compound, yielding **12a** in an 80 % yield.

^{1}H NMR: δ 0.72 (dd, J_1=2.0 Hz, J_2=11.8 Hz, H5), 0.73 (dd, J_1=2.0 Hz, J_2=11.8 Hz, H5*), 0.75 (s, H26''), 0.75 (s, H26*), 0.77 (s, H25), 0.77 (s, H26), 0.78 (s, H25''), 0.78 (dd, J_1=1.7 Hz, J_2=11.6 Hz, H5''), 0.78 (s, H25*), 0.87 (s, H29*), 0.87 (s, H29''), 0.89 (s, H30), 0.89 (s, H30''), 0.89 (s, H23*), 0.89 (s, H30*), 0.90 (s, H24''), 0.90 (s, H29), 0.91 (s, H23''), 0.92 (s, H24*), 0.92 (s, H24), 0.98 (s, H23), 1.13 (s, H27*), 1.15 (s, H27), 1.15 (s, H27''), 1.96 (dt, J_1=4.0 Hz, J_2=13.6 Hz, J_3=13.6 Hz, H11), 1.96 (dt, J_1=4.0 Hz, J_2=13.6 Hz, J_3=13.6 Hz, H11''), 1.99 (dt, J_1=3.8 Hz, J_2=13.8 Hz, J_3=13.8 Hz, H11*), 2.50 (bdd, J_1=4.4 Hz, J_2=13.5 Hz, J_3=13.5 Hz, H18''), 2.53 (bdd, J_1=4.4 Hz, J_2=13.5 Hz, J_3=13.5 Hz, H18), 2.84 (bdd, J_1=4.8 Hz, J_2=14.1 Hz, H18*), 2.98 (dd, J_1=4.2 Hz, J_2=11.6 Hz, H3''), 2.99 (dd, J_1=4.3 Hz, J_2=11.6 Hz, H3*), 3.21 (dd, J_1=4.6 Hz, J_2=11.3 Hz, H3), 3.47-3.53 (m, H1'''), 3.47-3.53 (m, H1'), 3.57-3.63 (m, H1'''), 3.57-3.63 (m, H1'), 4.56 (d, J=12.7 Hz, H11'), 4.56 (d, J=12.7 Hz, H11'''), 4.83 (d, J=12.7 Hz, H11'),

4.83 (d, *J*=12.7 Hz, H11'''), 5.29 (t, *J*=3.8 Hz, H12*), 5.35 (t, *J*=3.8 Hz, H12), 5.35 (t, *J*=3.8 Hz, H12''), 7.74 (s, H9'''), 7.76 (s, H9').

^{13}C NMR: δ 15.40 (C24''), 15.40 (C24*), 15.40 (C25), 16.50 (C25''), 16.50 (C24), 16.50 (C25*), 16.70 (C26''), 16.90 (C26), 17.10 (C26*), 18.20 (C6''), 18.20 (C6*), 18.30 (C6), 20.60 (C2), 22.50 (C16*), 22.60 (C16''), 23.40 (C11*), 23.40 (C2*), 23.50 (C2''), 23.50 (C11''), 23.50 (C30*), 23.50 (C16), 23.60 (C11), 23.60 (C30''), 23.70 (C30), 25.70 (C27), 25.70 (C27*), 25.90 (C27''), 27.90 (C15''), 28.00 (C15*), 28.10 (C23''), 28.10 (C15), 28.20 (C23*), 28.30 (C23), 30.70 (C20*), 30.70 (C20''), 30.70 (C20), 32.30 (C22''), 32.40 (C22*), 32.40 (C7*), 32.50 (C7''), 33.00 (C29*), 33.00 (C22), 33.10 (C7), 33.10 (C29), 33.40 (C29''), 33.90 (C21''), 33.90 (C21*), 34.10 (C21), 36.80 (C10''), 36.90 (C10*), 37.00 (C10), 38.10 (C1*), 38.20 (C1''), 38.70 (C4*), 38.70 (C4''), 38.80 (C1), 38.90 (C8), 39.10 (C8''), 39.30 (C8*), 39.40 (C1'''), 39.40 (C1'), 41.10 (C18*), 41.70 (C14*), 42.00 (C14), 42.00 (C18), 42.00 (C18''), 42.00 (C14''), 42.60 (C4), 45.90 (C19*), 46.30 (C19), 46.30 (C19''), 46.40 (C17''), 46.40 (C17*), 46.70 (C17), 47.50 (C9*), 47.50 (C9''), 51.90 (C9), 52.30 (C8'), 52.30 (C8'''), 55.00 (C5*), 55.10 (C5''), 55.40 (C5), 61.60 (C11'), 61.70 (C11'''), 69.70 (C7'''), 69.70 (C7'), 70.20 (C6'), 70.20 (C6'''), 70.40 (C5'''), 70.60 (C5'), 70.60 (C3'), 70.60 (C3'''), 70.60 (C4'''), 70.60 (C4'), 70.70 (C2'''), 70.70 (C2'), 78.90 (C3), 87.20 (C3''), 87.50 (C3*), 122.27 (C12*), 122.30 (C12), 122.37 (C12''), 125.20 (C9'''), 125.30 (C9'), 143.80 (C13*), 144.35 (C10'''), 144.40 (C10'), 144.70 (C13''), 144.70 (C13), 178.20 (C28), 180.70 (C28*), 181.70 (C28'').

MS (ES): *m/z* = 1847.4 $[M+H]^+$

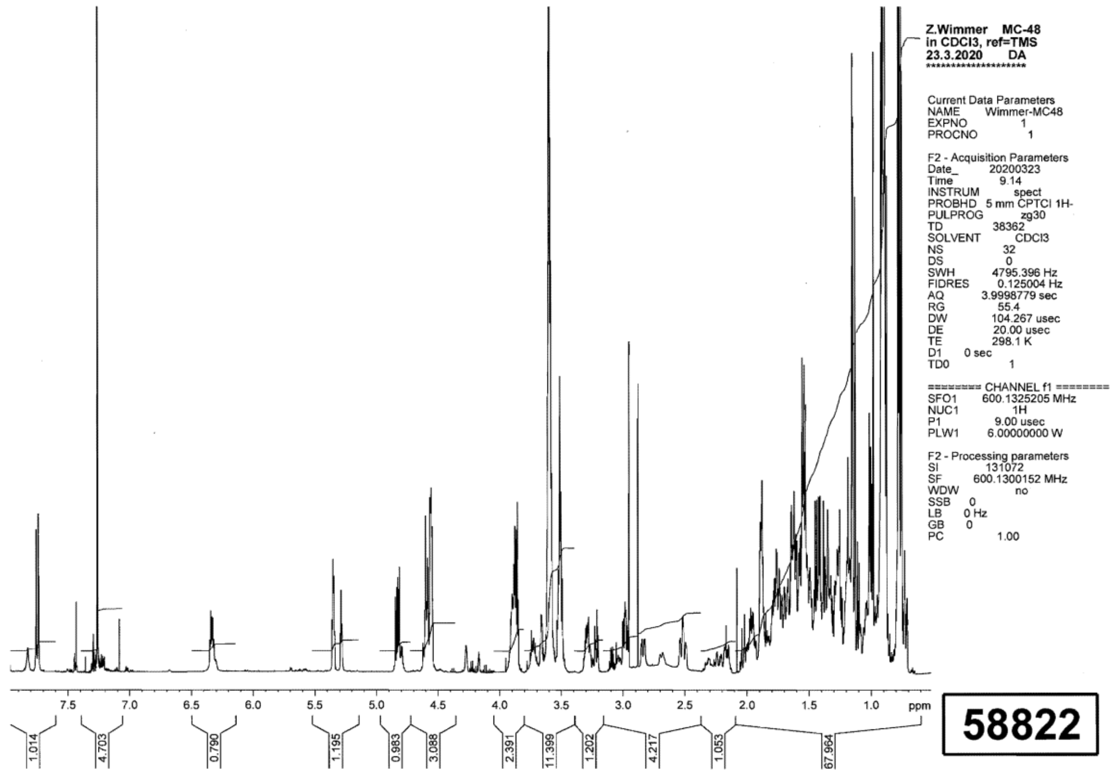
Z.Wimmer MC-48
in CDCl3, ref=TMS
23.3.2020 DA
Current Data Parameters
NAME Wimmer-MC48
EXPNO 1
PROCNO 1
F2 - Acquisition Parameters
Date_ 20200323
Time 9.14
INSTRUM spect
PROBHD 5 mm CPTCI 1H-
PULPROG zg30
TD 38362
SOLVENT CDCl3
NS 32
DS 0
SWH 4795.396 Hz
FIDRES 0.125004 Hz
AQ 3.9998779 sec
RG 55.4
DW 104.267 usec
DE 20.00 usec
TE 298.1 K
D1 0 sec
TD0 1
======== CHANNEL f1 ========
SFO1 600.1325205 MHz
NUC1 1H
P1 9.00 usec
PLW1 6.00000000 W
F2 - Processing parameters
SI 131072
SF 600.1300152 MHz
WDW no
SSB 0
LB 0 Hz
GB 0
PC 1.00
7.5 7.0 6.5 6.0 5.5 5.0 4.5 4.0 3.5 3.0 2.5 2.0 1.5 1.0 ppm
1.014 4.703 0.790 1.195 0.983 3.088 2.391 11.399 1.202 4.217 1.053 67.964
58822

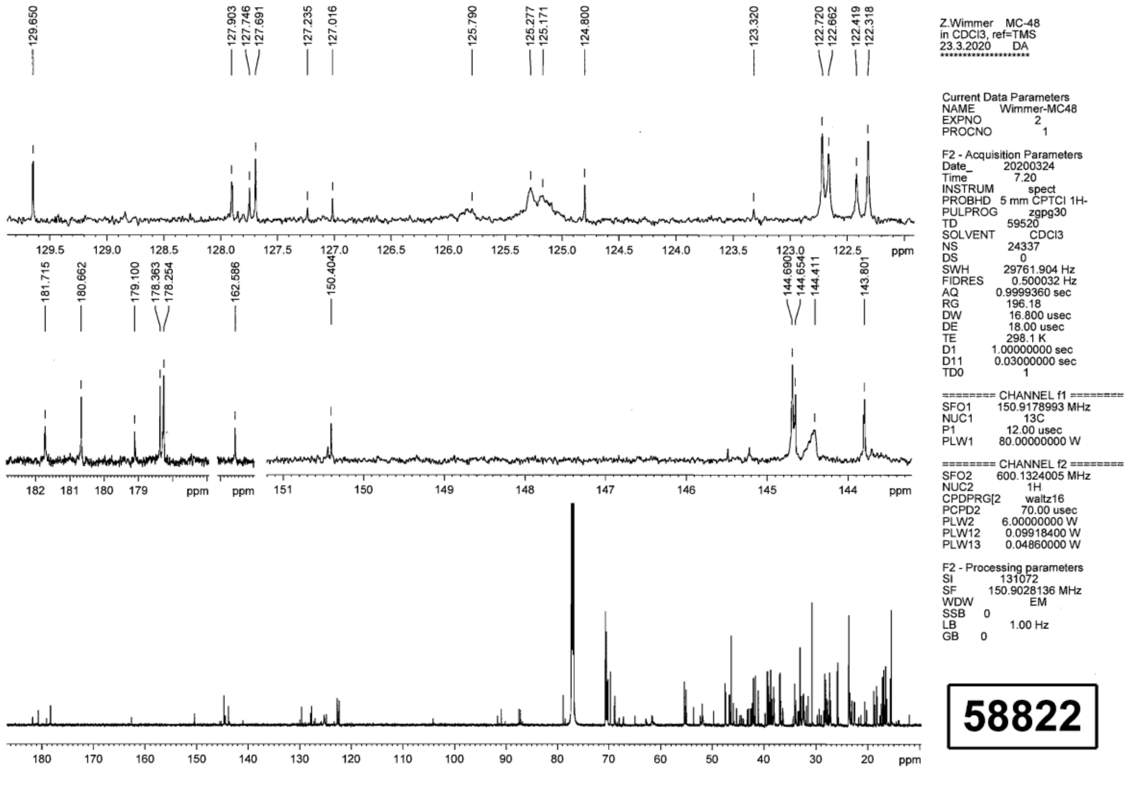
129.650 127.903 127.746 127.691 127.235 127.016 125.790 125.277 125.171 124.800 123.320 122.720 122.662 122.419 122.318
129.5 129.0 128.5 128.0 127.5 127.0 126.5 126.0 125.5 125.0 124.5 124.0 123.5 123.0 122.5 ppm
181.715 180.662 179.100 178.363 178.254 162.586 150.404 144.690 144.654 144.411 143.801
182 181 180 179 ppm ppm 151 150 149 148 147 146 145 144 ppm
180 170 160 150 140 130 120 110 100 90 80 70 60 50 40 30 20 ppm
Z.Wimmer MC-48
in CDCl3, ref=TMS
23.3.2020 DA
Current Data Parameters
NAME Wimmer-MC48
EXPNO 2
PROCNO 1
F2 - Acquisition Parameters
Date_ 20200324
Time 7.20
INSTRUM spect
PROBHD 5 mm CPTCI 1H-
PULPROG zgpg30
TD 59520
SOLVENT CDCl3
NS 24337
DS 0
SWH 29761.904 Hz
FIDRES 0.500032 Hz
AQ 0.9999360 sec
RG 196.18
DW 16.800 usec
DE 18.00 usec
TE 298.1 K
D1 1.00000000 sec
D11 0.03000000 sec
TD0 1
======== CHANNEL f1 ========
SFO1 150.9178993 MHz
NUC1 13C
P1 12.00 usec
PLW1 80.00000000 W
======== CHANNEL f2 ========
SFO2 600.1324005 MHz
NUC2 1H
CPDPRG[2 waltz16
PCPD2 70.00 usec
PLW2 6.00000000 W
PLW12 0.09918400 W
PLW13 0.04860000 W
F2 - Processing parameters
SI 131072
SF 150.9028136 MHz
WDW EM
SSB 0
LB 1.00 Hz
GB 0
58822

1.12. Benzyl (3β)-3-hydroxyolean-18-en-28-oate (**2b**)

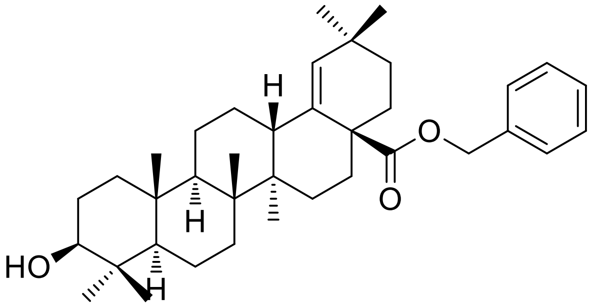

Procedure A was applied, using **1b** as the starting compound, yielding **2b** in a 96 % yield.

^{1}H NMR: δ 0.66 (1H, dd, J_1=2.2 Hz, J_2=11.5 Hz, H5), 0.74 (3H, d, J=0.5 Hz, H27), 0.75 (3H, s, H26), 0.76 (3H, d, J=0.6 Hz, H25), 0.82 (3H, d, J=0.8 Hz, H24), 0.95 (3H, s, H23), 0.95 (3H, s, H30), 0.97 (3H, s, H29), 1.72 (2H, dt, J_1=3.6 Hz, J_2=3.6 Hz, J_3=13.0 Hz, H1), 1.92 (2H, ddd, J_1=3.3 Hz, J_2=6.1 Hz, J_3=13.8 Hz, H7), 2.04 (1H, ddd, J_1=1.8 Hz, J_2=3.8 Hz, J_3=12.0 Hz, H13), 2.17 (3H, ddd, J_1=3.1 Hz, J_2=3.8 Hz, J_3=13.3 Hz, H16), 3.19 (1H, dd, J_1=4.8 Hz, J_2=11.5 Hz, H3), 5.09 (2H, d, J=12.4 Hz, H1'), 5.11 (1H, dd, J_1=0.9 Hz, J_2=1.8 Hz, H19), 5.17 (2H, d, J=12.4 Hz, H1'), 7.20-7.28 (1H, m, H4'), 7.20-7.28 (1H, m, H3'), 7.20-7.28 (1H, m, H5').

^{13}C NMR: δ 14.91 (q, C27), 15.37 (q, C26), 15.81 (q, C25), 16.63 (q, C24), 18.18 (t, C6), 20.88 (t, C11), 20.88 (t, C2), 25.97 (t, C12), 27.38 (t, C15), 27.93 (q, C23), 29.29 (q, C30), 30.37 (q, C29), 31.98 (s, C20), 33.35 (t, C21), 33.44 (t, C7), 33.46 (t, C16), 34.52 (t, C22), 37.17 (s, C10), 38.86 (s, C8), 38.91 (t, C1), 40.57 (s, C14), 41.30 (d, C13), 42.47 (s, C4), 48.15 (s, C17), 51.13 (d, C9), 55.46 (d, C5), 66.04 (t, C1'), 78.95 (d, C3), 127.94 (d, C5'), 128.04 (d, C3'), 128.42 (d, C4'), 132.47 (d, C19), 136.49 (s, C2'), 137.14 (s, C18), 176.39 (s, C28).

MS (ES): m/z = 547.3 $[M+H]^+$

1.13. Benzyl (3β)-3-(prop-2-yn-1-yloxy)olean-18-en-28-oate (**3b**)

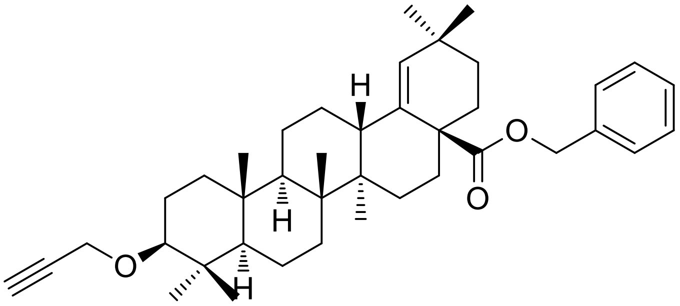

Procedure B was applied, using **2b** as the starting compound, yielding **3b** in a 94 % yield.

^{1}H NMR: δ 0.68 (1H, dd, J_1=2.1 Hz, J_2=11.4 Hz, H5), 0.74 (3H, d, J=0.6 Hz, H27), 0.75 (3H, s, H26), 0.76 (3H, d, J=0.5 Hz, H25), 0.83 (3H, d, J=0.8 Hz, H24), 0.95 (3H, s, H30), 0.96 (3H, s, H23), 0.97 (3H, s, H29), 1.76 (2H, dt, J_1=3.6 Hz, J_2=3.6 Hz, J_3=12.9 Hz, H1), 1.92 (2H, ddd, J_1=3.3 Hz, J_2=6.1 Hz, J_3=13.8 Hz, H7), 2.04 (1H, ddd, J_1=1.7 Hz, J_2=3.6 Hz, J_3=11.7 Hz, H13), 2.11 (1H, dd, J_1=0.9 Hz, J_2=1.8 Hz, H19), 2.17 (2H, ddd, J_1=3.0 Hz, J_2=3.9 Hz, J_3=13.4 Hz, H16), 2.36 (1H, t, J=2.3 Hz, H8'), 3.00 (1H, dd, J_1=4.4 Hz, J_2=11.8 Hz, H3), 4.14 (2H, dd, J_1=2.3 Hz, J_2=16.0 Hz, H6'), 4.22 (2H, dd, J_1=2.3 Hz, J_2=16.0 Hz, H6'), 5.09 (2H, d, J=12.4 Hz, H1'), 5.16 (2H, d, J=12.4 Hz, H1'), 7.28-7.36 (1H, m, H4'), 7.28-7.36 (1H, m, H3'), 7.28-7.36 (1H, m, H5').

^{13}C NMR: δ 14.88 (q, C27), 15.82 (q, C26), 16.20 (q, C25), 16.62 (q, C24), 18.12 (t, C6), 20.91 (t, C11), 22.60 (t, C2), 25.99 (t, C12), 27.92 (q, C23), 27.92 (t, C15), 29.15 (q, C30), 29.27 (t, C20), 30.38 (q, C29), 31.98 (t, C16), 31.98 (t, C21), 33.35 (t, C7), 34.53 (t, C22), 37.12 (s, C10), 38.59 (s, C8), 38.78 (t, C1), 40.62 (s, C14), 41.29 (d, C13), 42.46 (s, C4), 48.15 (s, C17), 51.11 (d, C9), 56.03 (d, C5), 56.42 (t, C6'), 66.04 (t, C1'), 73.41 (d, C8'), 80.95 (s, C7'), 85.82 (d, C3), 127.94 (d, C5'), 128.04 (d, C3'), 128.42 (d, C4'), 132.44 (d, C19), 136.48 (s, C2'), 137.17 (s, C18), 176.37 (s, C28).

MS (ES): *m/z* = 585.3 $[M+H]^+$

1.14. (3β)-3-(Acetyloxy)olean-18-en-28-oic acid (**4b**)

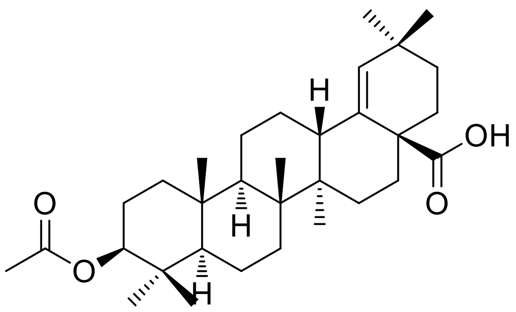

Procedure C was applied, using **1b** as the starting compound, yielding **4b** in a 98 % yield.

^{1}H NMR: δ 0.78 (3H, d, J=0.5 Hz, H27), 0.81 (1H, dd, J_1=2.1 Hz, J_2=11.4 Hz, H5), 0.84 (3H, s, H25), 0.85 (3H, s, H23), 0.90 (3H, d, J=0.8 Hz, H24), 0.98 (3H, s, H30), 1.00 (3H, d, J=0.5 Hz, H26), 1.00 (3H, s, H29), 1.03 (2H, bdt, J_1=4.8 Hz, J_2=13.0 Hz, J_3=13.0 Hz, H1), 1.21 (2H, ddd, J_1=3.0 Hz, J_2=3.9 Hz, J_3=13.4 Hz, H15), 1.75 (2H, dt, J_1=3.6 Hz, J_2=3.6 Hz, J_3=13.2 Hz, H1), 2.00 (2H, ddd, J_1=3.4 Hz, J_2=602.0 Hz, J_3=13.6 Hz, H7), 2.04 (3H, s, OAc), 2.17 (2H, ddd, J_1=3.0 Hz, J_2=4.0 Hz, J_3=13.5 Hz, H16), 2.23 (1H, ddd, J_1=1.9 Hz, J_2=3.4 Hz, J_3=11.4 Hz, H13), 4.49 (1H, dd, J_1=5.3 Hz, J_2=11.3 Hz, H3), 5.18 (1H, dd, J_1=0.9 Hz, J_2=1.9 Hz, H19).

^{13}C NMR: δ 14.85 (q, C27), 16.05 (q, C26), 16.51 (q, C25), 16.69 (q, C24), 18.19 (t, C6), 21.00 (t, C11), 21.23 (q, OAc), 23.75 (t, C2), 26.02 (t, C12), 27.96 (q, C23), 29.12 (q, C30), 29.41 (t, C15), 30.32 (q, C29), 32.08 (s, C20), 33.42 (t, C16), 33.55 (t, C7), 33.56 (t, C21), 34.58 (t, C22), 37.24 (s, C10), 37.89 (s, C8), 38.71 (t, C1), 40.79 (s, C14), 41.42 (d, C13), 42.67 (s, C4), 48.00 (s, C17), 51.17 (d, C9), 55.70 (d, C5), 81.00 (d, C3), 133.36 (d, C19), 136.77 (s, C18), 170.94 (s, OAc), 180.54 (s, C28).

MS (ES): m/z = 516.2 $[M+NH_4]^+$; 496.9 $[M-H]^-$

1.15. 28-[(2-{2-[2-(2-Azidoethoxy)ethoxy]ethoxy}ethyl)amino]-28-oxoolean-18-en-3-yl acetate (**5b**)

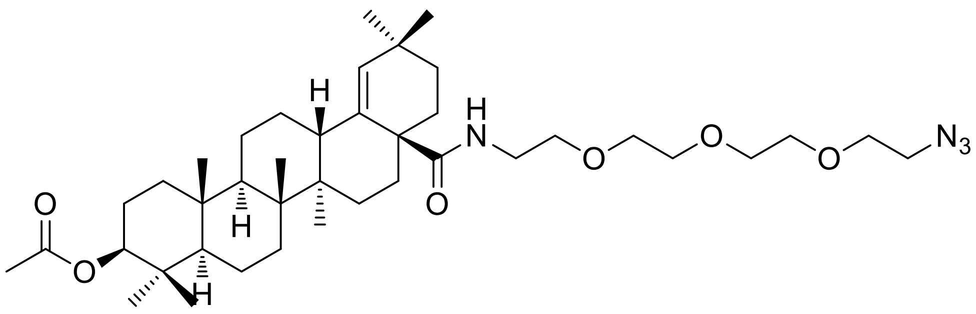

Procedure E was applied, using **4b** as the starting compound, yielding **5b** in a 99 % yield.

^{1}H NMR: δ 0.75 (3H, s, H27), 0.79 (1H, dd, J_1=2.0 Hz, J_2=11.4 Hz, H5), 0.83 (3H, s, H23), 0.84 (3H, s, H25), 0.88 (3H, d, J=0.7 Hz, H24), 0.95 (3H, s, H26), 0.98 (3H, s, H30), 1.00 (3H, s, H29), 1.74 (2H, dt, J_1=3.5 Hz, J_2=3.5 Hz, J_3=13.0 Hz, H1), 1.83 (2H, ddd, J_1=3.5 Hz, J_2=4.9 Hz, J_3=13.9 Hz, H7), 2.04 (3H, s, H10'), 2.12 (1H, ddd, J_1=1.6 Hz, J_2=3.3 Hz, J_3=11.6 Hz, H13), 2.42 (2H, dt, J_1=3.5 Hz, J_2=3.5 Hz, J_3=13.4 Hz, H16), 3.37-3.68 (2H, m, H6'), 3.37-3.40 (2H, m, H8'), 3.37-3.68 (2H, m, H5'), 3.37-3.68 (2H, m, H7'), 3.37-3.68 (2H, m, H3'), 3.37-3.68 (2H, m, H4'), 3.37-3.68 (2H, m, H2'), 3.41-3.50 (2H, m, H1'), 4.74 (1H, dd, J_1=5.2 Hz, J_2=11.3 Hz, H3), 5.31 (1H, dd, J_1=0.7 Hz, J_2=1.8 Hz, H19), 6.25 (0H, 5.9, H1'-NH).

^{13}C NMR: δ 14.88 (q, C27), 16.01 (q, C26), 16.47 (q, C25), 16.76 (q, C24), 18.06 (t, C6), 21.01 (t, C11), 21.31 (q, C10'), 23.64 (t, C2), 26.21 (t, C12), 27.88 (q, C23), 28.92 (q, C30), 29.33 (t, C15), 30.89 (q, C29), 32.28 (s, C20), 32.70 (t, C21), 33.19 (t, C7), 33.94 (t, C16), 34.39 (t, C22), 37.10 (s, C10), 37.78 (s, C8), 38.60 (t, C1), 39.34 (t, C1'), 40.69 (s, C14), 41.53 (d, C13), 42.60 (s, C4), 48.21 (s, C17), 50.65 (t, C8'), 51.05 (d, C9), 55.56 (d, C5), 70.08 (t, C7'), 70.35 (t, C6'), 70.37 (t, C5'), 70.57 (t, C4'), 70.63 (t, C3'), 70.69 (t, C2'), 80.86 (d, C3), 135.62 (d, C19), 138.40 (s, C18), 171.03 (s, C9'), 175.55 (s, C28).

MS (ES): *m/z* = 699.2 $[M+H]^+$; 697.2 $[M-H]^-$

1.16. Benzyl (3β)-3-{[1-({2-[2-(2-{[3-(acetyloxy)-28-oxoolean-18-en-28-yl]amino}ethoxy)-ethoxy]ethoxy}methyl)-1*H*-1,2,3-triazol-4-yl]methoxy}olean-18-en-28-oate (**6b**)

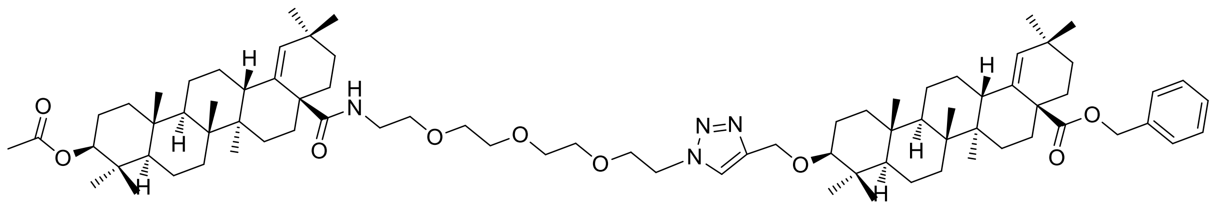

Procedure F was applied, using **5b** and **3b** as the starting compounds, yielding **6b** in a 99 % yield.

^{1}H NMR: δ 0.66 (1H, dd, J_1=2.0 Hz, J_2=11.4 Hz, H5''), 0.73 (3H, s, H27''), 0.74 (3H, s, H25''), 0.75 (3H, s, H27), 0.75 (3H, s, H26''), 0.79 (1H, dd, J_1=2.0 Hz, J_2=11.3 Hz, J_3=13.6 Hz, H5), 0.82 (3H, d, J=0.6 Hz, H24''), 0.83 (3H, s, H25), 0.84 (3H, s, H23), 0.87 (3H, s, H23''), 0.87 (3H, d, J=0.6 Hz, H24), 0.94 (3H, s, H26), 0.96 (3H, s, H30''), 0.97 (3H, s, H29''), 0.98 (3H, s, H30), 0.99 (3H, s, H29), 1.73 (2H, dt, J_1=3.6 Hz, J_2=3.6 Hz, J_3=13.5 Hz, H1), 1.75 (2H, dt, J_1=3.6 Hz, J_2=3.6 Hz, J_3=13.5 Hz, H1''), 1.82 (2H, ddd, J_1=3.5 Hz, J_2=4.9 Hz, J_3=13.8 Hz, H7), 1.91 (2H, ddd, J_1=3.3 Hz, J_2=6.0 Hz, J_3=13.7 Hz, H7''), 2.04 (3H, s, OAc), 2.10 (1H, m, J=13.1 Hz, H13), 2.10-2.13 (1H, m, H13''), 2.16 (2H, ddd, J_1=3.2 Hz, J_2=3.6 Hz, J_3=13.4 Hz, H16''), 2.42 (2H, dt, J_1=3.5 Hz, J_2=3.5 Hz, J_3=13.4 Hz, H16), 2.96 (2H, dd, J_1=4.4 Hz, J_2=11.8 Hz, H3''), 3.37-3.42 (2H, m, H1'), 3.46-3.52 (2H, m, H1'), 3.50-3.62 (2H, m, H3'), 3.50-3.62 (2H, m, H2'), 3.50-3.62 (2H, m, H4'), 3.50-3.62 (2H, m, H6'), 3.50-3.62 (2H, m, H5'), 3.87 (2H, t, J=5.3 Hz, H7'), 4.47 (1H, dd, J_1=5.2 Hz, J_2=11.4 Hz, H3), 4.55 (2H, t, J=5.3 Hz, H8'), 4.59 (2H, d, J=12.7 Hz, H11'), 4.80 (2H, d, J=12.7 Hz, H11'), 5.09 (2H, d, J=12.4 Hz, H1'''), 5.11 (1H, dd, J_1=0.6 Hz, J_2=1.8 Hz, H19''), 5.11 (2H, d, J=12.4 Hz, H1'''), 5.30 (1H, dd, J_1=0.6 Hz, J_2=11.8 Hz, H19), 7.28-7.37 (1H, m, H5'''), 7.28-7.35 (1H, m, H3'''), 7.28-7.36 (1H, m, H4'''), 7.72 (1H, s, H9').

^{13}C NMR: δ 14.92 (q, C27''), 14.92 (q, C27), 15.84 (q, C26''), 16.05 (q, C26), 16.32 (q, C25''), 16.53 (q, C25), 16.68 (q, C24''), 16.80 (q, C24), 18.06 (t, C6), 18.15 (t, C6''), 20.92 (t, C11''), 21.04 (t, C11), 21.34 (q, OAc), 22.85 (t, C2''), 23.67 (t, C2), 25.98 (t, C12''), 26.27 (t, C12), 27.91 (q, C23), 28.02 (q, C23''), 28.96 (q, C30), 29.17 (q, C30''), 29.30 (t, C15''), 29.35 (t, C15), 30.39 (q, C29''), 30.95 (q, C29), 32.00 (s, C20''), 32.31 (t, C21''), 32.31 (s, C20), 32.71 (t, C21), 33.22 (t, C7), 33.37 (t, C7''), 33.47 (t, C16''), 33.47 (t, C16), 34.41 (t, C22), 34.53 (t, C22''), 37.13 (s, C10''), 37.15 (s, C10), 37.81 (s, C8), 37.81 (s, C8''), 38.64 (t, C1), 38.82 (t, C1''), 39.35 (t, C1'), 40.62 (s, C14''), 40.72 (s, C14), 41.32 (d, C13''), 41.58 (d, C13), 42.48 (s, C4''), 42.64 (s, C4), 48.16 (s, C17''), 48.24 (s, C17), 50.94 (t, C8'), 51.06 (d, C9), 51.09 (d, C9''), 55.58 (d, C5), 55.83 (d, C5''), 62.46 (t, C11'), 66.05 (t, C1'''), 69.22 (t, C7'), 70.37 (t, C6'), 70.37 (t, C5'),

70.49 (t, C4'), 70.55 (t, C3'), 70.59 (t, C2'), 80.85 (d, C3), 86.49 (d, C3''), 124.15 (d, C9'), 128.05 (d, C4'''), 128.44 (d, C3'''), 128.96 (d, C5'''), 132.52 (d, C19''), 135.62 (d, C19), 136.51 (s, C2'''), 137.12 (s, C18''), 138.48 (s, C18), 145.51 (s, C10'), 171.05 (s, OAc), 175.59 (s, C28), 176.42 (s, C28'').

MS (ES): *m/z* = 1284.0 $[M+H]^+$; 1281.9 $[M-H]^-$

1.17. (3β)-3-{[1-({2-[2-(2-{[3-(Acetyloxy)-28-oxoolean-18-en-28-yl]amino}ethoxy)ethoxy]-ethoxy}ethyl)-1*H*-1,2,3-triazol-4-yl]methoxy}olean-18-en-28-oic acid (**7b**)

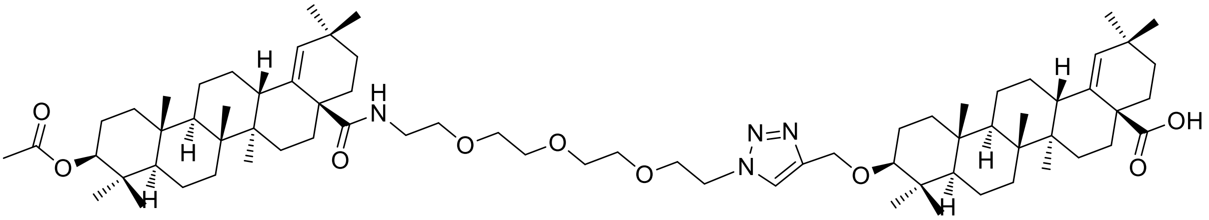

Procedure G was applied, using **6b** as the starting compound, yielding **7b** in a 98 % yield.

^{1}H NMR: δ 0.70 (1H, dd, J_1=2.0 Hz, J_2=11.2 Hz, H5''), 0.77 (3H, s, H27''), 0.78 (3H, s, H25''), 0.79 (3H, s, H27), 0.81 (1H, dd, J_1=1.8 Hz, J_2=11.3 Hz, H5), 0.85 (3H, s, H25), 0.86 (3H, s, H23), 0.88 (3H, s, H24''), 0.90 (3H, s, H24), 0.93 (3H, s, H23''), 0.97 (3H, s, H26''), 0.99 (3H, s, H30''), 1.00 (3H, s, H30), 1.00 (3H, s, H26), 1.02 (3H, s, H29), 1.02 (3H, s, H29''), 1.75 (2H, dt, J_1=3.7 Hz, J_2=3.7 Hz, J_3=13.3 Hz, H1), 1.80 (2H, dt, J_1=3.6 Hz, J_2=3.6 Hz, J_3=13.2 Hz, H1''), 1.84 (2H, ddd, J_1=3.7 Hz, J_2=4.6 Hz, J_3=14.1 Hz, H21), 2.00 (2H, ddd, J_1=3.4 Hz, J_2=6.0 Hz, J_3=13.8 Hz, H7''), 2.18 (2H, dt, J_1=3.4 Hz, J_2=3.4 Hz, J_3=13.4 Hz, H16''), 2.43 (2H, dt, J_1=3.4 Hz, J_2=3.4 Hz, J_3=13.2 Hz, H16), 3.03 (1H, dd, J_1=4.4 Hz, J_2=11.8 Hz, H3''), 3.39-3.45 (2H, m, H1'), 3.50-3.54 (2H, m, H1'), 3.51-3.66 (2H, m, H3'), 3.51-3.66 (2H, m, H2'), 3.51-3.66 (2H, m, H5'), 3.51-3.66 (2H, m, H4'), 3.51-3.66 (2H, m, H6'), 3.93-3.96 (2H, m, H7'), 4.49 (1H, dd, J_1=5.0 Hz, J_2=11.5 Hz, H3), 4.67 (2H, t, *J*=4.6 Hz, H8'), 4.77 (1H, bd, *J*=13.3 Hz, H11'), 4.98 (1H, bd, *J*=13.3 Hz, H11'), 5.20 (1H, bd, *J*=1.6 Hz, H19''), 5.33 (1H, bd, *J*=1.6 Hz, H19), 7.99 (1H, s, H9').

^{13}C NMR: δ 14.89 (q, C27), 14.92 (q, C27''), 16.01 (q, C26''), 16.03 (q, C26), 16.28 (t, C12), 16.31 (q, C25''), 16.50 (q, C25), 16.69 (q, C24''), 16.77 (q, C24), 18.06 (t, C6), 18.13 (t, C6''), 20.92 (t, C11''), 21.01 (t, C11), 21.34 (q, OAc), 22.73 (t, C2''), 23.61 (t, C2), 25.94 (t, C12''), 27.87 (q, C23), 28.11 (q, C23''), 28.93 (q, C30), 29.08 (q, C30''), 29.31 (t, C15''), 29.36 (t, C15),

30.33 (q, C29''), 30.93 (q, C29), 32.06 (t, C20''), 32.29 (s, C20), 32.66 (t, C21), 32.66 (t, C21''), 33.18 (t, C7), 33.35 (t, C7''), 33.44 (t, C16''), 33.50 (t, C16), 34.37 (t, C22), 34.45 (t, C22''), 37.09 (s, C10), 37.13 (s, C10''), 37.77 (s, C8), 37.77 (s, C8''), 38.62 (t, C1''), 38.83 (t, C1), 39.38 (t, C1'), 40.67 (s, C14''), 40.69 (s, C14), 41.36 (d, C13''), 41.57 (d, C13), 42.58 (s, C4''), 42.62 (s, C4), 47.88 (s, C17''), 48.22 (s, C17), 51.00 (d, C9''), 51.03 (d, C9), 52.67 (t, C8'), 55.53 (d, C5), 55.68 (d, C5''), 60.84 (t, C11'), 68.50 (t, C7'), 70.26 (t, C6'), 70.33 (t, C5'), 70.41 (t, C4'), 70.55 (t, C3'), 70.62 (t, C2'), 80.93 (d, C3), 87.81 (d, C3''), 126.00 (t, C9'), 133.34 (d, C19''), 135.67 (d, C19), 136.79 (s, C18''), 138.39 (s, C18), 145.48 (t, C10'), 171.23 (s, OAc), 175.75 (s, C28), 179.34 (s, C28'').

MS (ES): *m/z* = 1193.8 $[M+H]^+$; 1191.7 $[M-H]^-$

1.18. (3β)-3-{[1-(2-{2-[2-(2-{[(3β)-3-hydroxy-28-oxoolean-18-en-28-yl]amino}ethoxy)-ethoxy]ethoxy}ethyl)-1*H*-1,2,3-triazol-4-yl]methoxy}olean-18-en-28-oic acid (**8b**)

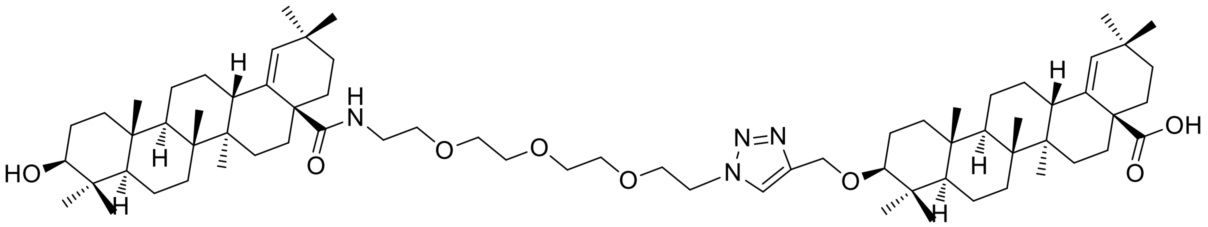

Procedure H was applied, using **7b** as the starting compound, yielding **8b** in a 99 % yield.

^{1}H NMR: δ 0.68 (1H, dd, J_1=2.0 Hz, J_2=11.5 Hz, H5''), 0.75 (3H, s, H25), 0.75 (3H, s, H27''), 0.76 (3H, s, H27), 0.76 (3H, s, H25''), 0.85 (3H, s, H24), 0.86 (3H, s, H24''), 0.88 (3H, s, H23), 0.94 (3H, s, H26''), 0.94 (3H, s, H26), 0.96 (3H, s, H23''), 0.97 (3H, s, H30), 0.97 (3H, s, H30''), 0.99 (3H, s, H29''), 0.99 (3H, s, H29), 1.73 (2H, dt, J_1=3.6 Hz, J_2=3.6 Hz, J_3=13.0 Hz, H1), 1.77 (2H, dt, J_1=3.5 Hz, J_2=3.5 Hz, J_3=13.6 Hz, H1''), 1.83 (2H, ddd, J_1=3.6 Hz, J_2=4.8 Hz, J_3=13.8 Hz, H21), 1.98 (2H, ddd, J_1=3.2 Hz, J_2=6.0 Hz, J_3=13.7 Hz, H7''), 2.12 (1H, bd, J=10.5 Hz, H13''), 2.16 (2H, dt, J_1=3.5 Hz, J_2=3.5 Hz, J_3=13.7 Hz, H16''), 2.21 (1H, bd, J=11.7 Hz, H13), 2.42 (2H, dt, J_1=3.6 Hz, J_2=3.6 Hz, J_3=13.4 Hz, H16), 2.97 (1H, dd, J_1=4.2 Hz, J_2=11.6 Hz, H3''), 3.21 (1H, dd, J_1=4.7 Hz, J_2=11.6 Hz, H3), 3.39-3.44 (2H, m, H1'), 3.48-3.52 (2H, m, H1'), 3.56-3.62 (2H, m, H3'), 3.56-3.62 (2H, m, H6'), 3.56-3.62 (2H, m, H5'), 3.56-3.62 (2H, m, H2'), 3.56-3.62 (2H, m, H4'), 3.86 (2H, t, J=5.1 Hz, H7'), 4.54 (2H, t, J=35.1 Hz, H8'), 4.58 (2H, d, J=12.5 Hz, H11'), 4.78 (2H, d, J=12.5 Hz, H11'), 5.17 (1H, bd, J=1.6 Hz, H19''), 5.30 (1H, bd, J=1.7 Hz, H19), 7.70 (1H, bs, H9').

^{13}C NMR: δ 14.88 (q, C27), 14.91 (q, C27''), 15.40 (q, C26), 16.03 (q, C25), 16.05 (q, C26''), 16.32 (q, C25''), 16.68 (q, C24''), 16.71 (q, C24), 18.15 (t, C6), 18.18 (t, C6''), 20.94 (t, C11''), 20.99 (t, C11), 20.99 (t, C2), 22.87 (t, C2''), 25.98 (t, C12''), 26.27 (t, C12), 27.92 (q, C23), 28.01 (q, C23''), 28.94 (q, C30), 29.09 (q, C30''), 29.32 (t, C15''), 29.37 (t, C15), 30.34 (q, C29''), 30.92 (q, C29), 32.05 (s, C20''), 32.28 (s, C20), 32.69 (t, C21), 33.19 (t, C7), 33.35 (t, C21''), 33.46 (t, C16''), 33.48 (t, C16), 33.94 (t, C7''), 34.45 (t, C22), 34.50 (t, C22''), 37.16 (s, C10), 37.17 (s, C10''), 38.65 (t, C1''), 38.82 (s, C8''), 38.85 (s, C8), 38.94 (t, C1), 39.32 (t, C1'), 40.67 (s, C14''), 40.67 (s, C14), 41.38 (d, C13''), 41.57 (d, C13), 42.57 (s, C4''), 42.62 (s, C4), 47.90 (s, C17''), 48.22 (s, C17), 50.92 (t, C8'), 51.06 (d, C9''), 51.11 (d, C9), 55.45 (d, C5), 55.81 (d, C5''), 62.49 (t, C11'), 69.26 (t, C7'), 70.31 (t, C6'), 70.37 (t, C5'), 70.45 (t, C4'), 70.51 (t, C3'), 70.56 (t, C2'), 78.95 (d, C3), 86.75 (d, C3''), 126.97 (d, C9'), 133.23 (d, C19''), 135.55 (d, C19), 136.86 (s, C18''), 138.51 (s, C18), 145.50 (s, C10'), 175.58 (s, C28), 179.76 (s, C28'').

MS (ES): *m/z* = 1151.7 $[M+H]^+$; 1149.8 $[M-H]^-$

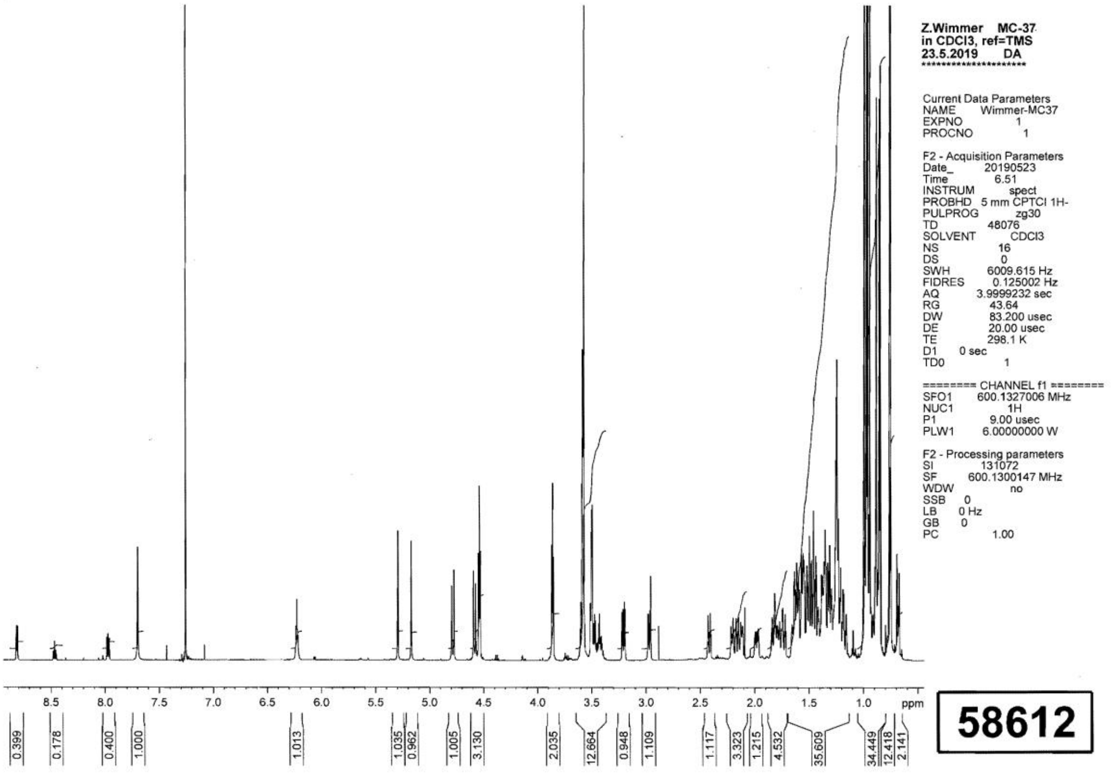

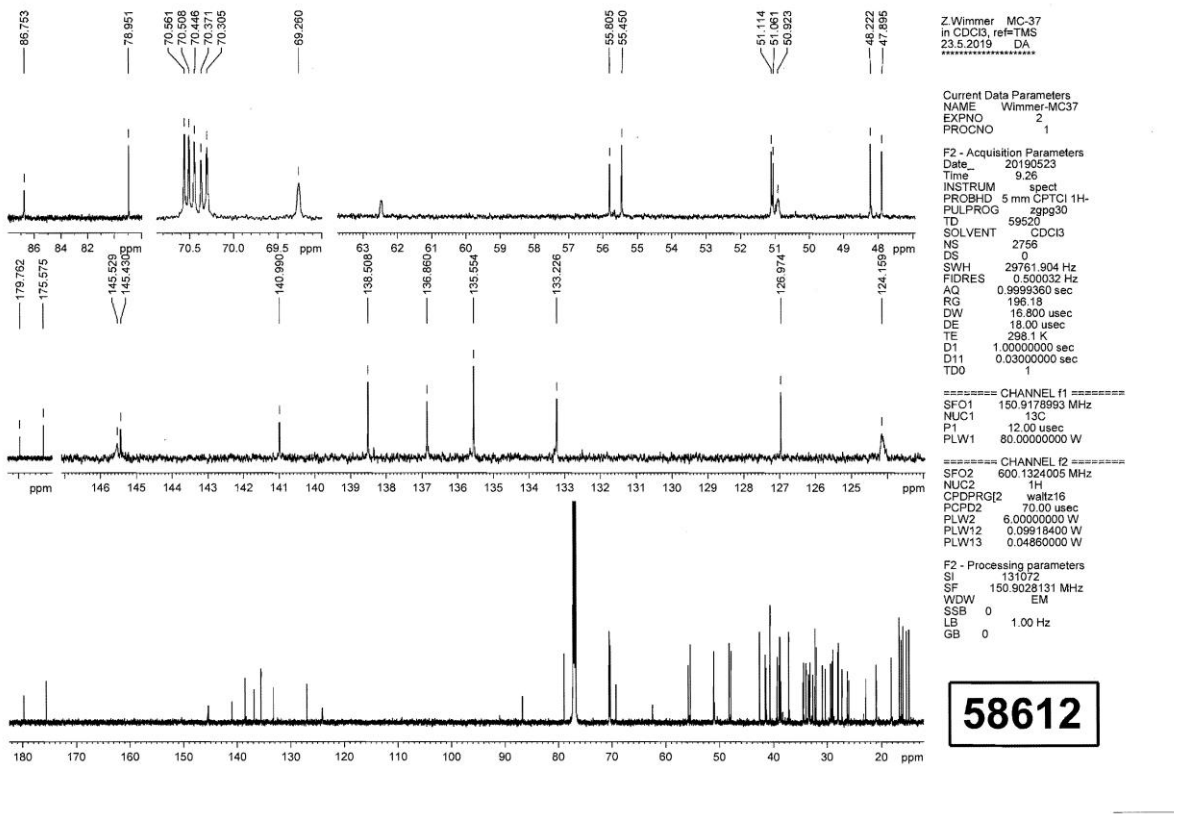

1.19. (3β)-28-[(2-{2-[2-(2-{4-[({(3β)-28-[(2-{2-[2-(2-Azidoethoxy)ethoxy]ethoxy}ethyl)-amino]-28-oxoolean-18-en-3-yl}oxy)methyl]-1*H*-1,2,3-triazol-1-yl}ethoxy)ethoxy]ethoxy}-ethyl)amino]-28-oxoolean-18-en-3-yl acetate (**9b**)

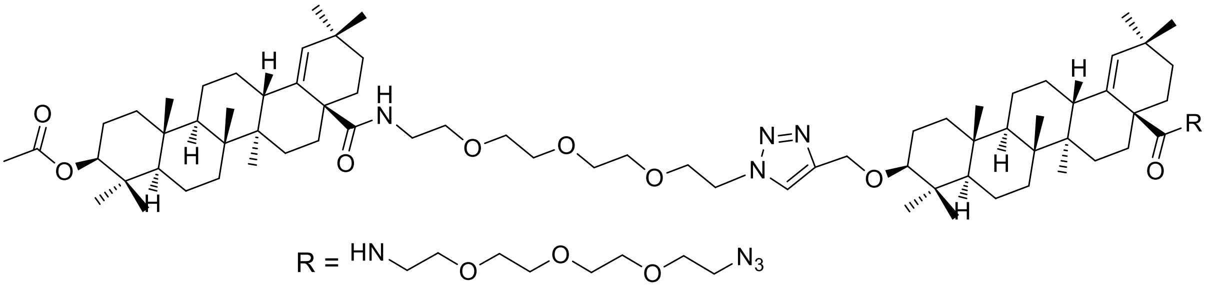

Procedure E was applied, using **7b** as the starting compound, yielding **9b** in a 50 % yield.

^{1}H NMR: δ 0.69 (1H, dd, J_1=2.0 Hz, J_2=11.3 Hz, H5''), 0.75 (3H, d, H27''), 0.75 (3H, s, H27), 0.79 (1H, dd, J_1=1.8 Hz, J_2=11.5 Hz, H5), 0.83 (3H, s, H25), 0.83 (3H, s, H25''), 0.84 (3H, s, H23), 0.86 (3H, s, H24''), 0.88 (3H, s, H24), 0.89 (3H, s, H23''), 0.94 (3H, s, H26''), 0.94 (3H, s, H26), 0.98 (3H, s, H30''), 0.98 (3H, s, H30), 0.99 (3H, s, H29''), 1.01 (3H, s, H29), 1.75 (2H, dt, J_1=3.5 Hz, J_2=3.5 Hz, J_3=13.3 Hz, H1), 1.79 (2H, dt, J_1=3.5 Hz, J_2=3.5 Hz, J_3=13.5 Hz,

H1''), 2.10-2.14 (1H, m, H13''), 2.10-2.14 (1H, m, H13), 2.40 (2H, dt, J_1=3.3 Hz, J_2=3.3 Hz, J_3=13.5 Hz, H16''), 2.41 (2H, dt, J_1=3.3 Hz, J_2=3.3 Hz, J_3=13.5 Hz, H16), 2.99 (1H, dd, J_1=4.4 Hz, J_2=11.8 Hz, H3''), 3.37-3.40 (2H, m, H8*), 3.39-3.45 (2H, m, H1'), 3.40-3.46 (2H, m, H1*), 3.48-3.52 (2H, m, H1'), 3.58-3.63 (2H, m, H4*), 3.58-3.63 (2H, m, H5*), 3.58-3.63 (2H, m, H2*), 3.58-3.63 (2H, m, H3*), 3.58-3.63 (2H, m, H6*), 3.58-3.63 (2H, m, H7*), 3.60-3.68 (2H, m, H6'), 3.60-3.68 (2H, m, H4'), 3.60-3.68 (2H, m, H3'), 3.60-3.68 (2H, m, H5'), 3.60-3.68 (2H, m, H2'), 3.88-3.92 (2H, m, H7'), 4.47 (1H, dd, J_1=5.1 Hz, J_2=11.3 Hz, H3), 4.59-4.62 (2H, m, H8'), 4.68 (1H, d, J=13.1 Hz, H11'), 4.90 (2H, d, J=13.1 Hz, H11'), 5.31 (1H, dd, J_1=0.7 Hz, J_2=1.7 Hz, H19), 5.31 (1H, dd, J_1=0.8 Hz, J_2=1.6 Hz, H19''), 7.87 (2H, s, H9').

^{13}C NMR: δ 14.89 (q, C27), 14.92 (q, C27''), 16.03 (q, C26''), 16.03 (q, C26), 16.29 (q, C25''), 16.50 (q, C25), 16.72 (q, C24''), 16.78 (q, C24), 18.06 (t, C6), 18.20 (t, C6''), 21.01 (t, C11''), 21.01 (t, C11), 22.70 (t, C2''), 23.63 (t, C2), 26.20 (t, C12''), 26.25 (t, C12), 27.88 (q, C23), 28.03 (q, C23''), 28.93 (q, C30), 28.93 (q, C30''), 29.31 (t, C15''), 29.33 (t, C15), 30.88 (q, C29''), 30.93 (q, C29), 32.28 (s, C20''), 32.29 (s, C20), 32.68 (t, C16''), 32.70 (t, C16), 33.20 (t, C21), 33.20 (t, C21''), 33.42 (t, C22''), 33.95 (t, C7), 33.95 (t, C7''), 34.38 (t, C22), 37.10 (s, C10), 37.14 (s, C10''), 37.78 (s, C8), 37.78 (s, C8''), 38.61 (t, C1'), 38.79 (t, C1), 39.33 (t, C1''), 39.35 (t, C1*), 40.69 (s, C14''), 40.70 (s, C14), 41.54 (d, C13''), 41.56 (d, C13), 42.61 (s, C4''), 42.62 (s, C4), 48.21 (s, C17), 48.21 (s, C17''), 50.66 (t, C8*), 51.03 (d, C9), 51.03 (d, C9''), 51.84 (t, C8'), 55.54 (d, C5), 55.76 (d, C5''), 61.49 (t, C11'), 68.84 (t, C7'), 70.06 (t, C3'), 70.08 (t, C7*), 70.33 (t, C6*), 70.35 (t, C5*), 70.38 (t, C4*), 70.45 (t, C3*), 70.45 (t, C6'), 70.55 (t, C4'), 70.55 (t, C5'), 70.57 (t, C2*), 70.64 (t, C2'), 80.82 (d, C3), 87.17 (d, C3''), 125.15 (d, C9'), 135.62 (d, C19), 135.62 (d, C19''), 138.40 (s, C18''), 138.43 (s, C18), 144.47 (s, C10'), 175.56 (s, C28''), 175.63 (s, C28).

MS (ES): *m/z* = 1394.6 $[M+H]^+$; 1392.6 $[M-H]^-$

1.20. Benzyl (3β)-3-{[1-(2-{2-[2-(2-{[(3β)-3-{[1-(2-{2-[2-(2-{[(3β)-3-(acetyloxy)-28-oxoolean-18-en-28-yl]amino}ethoxy)ethoxy]ethoxy}ethyl)-1*H*-1,2,3-triazol-4-yl]methoxy}-28-oxoolean-18-en-28-yl]amino}ethoxy)ethoxy]ethoxy}ethyl)-1*H*-1,2,3-triazol-4-yl]-methoxy}olean-18-en-28-oate (**10b**)

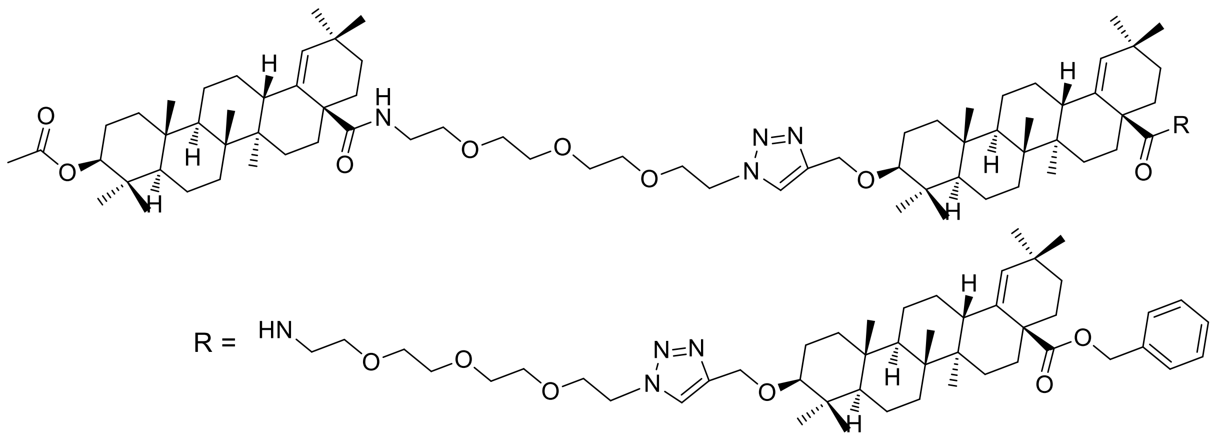

Procedure F was applied, using **9b** and **3b** as the starting compounds, yielding **10b** in a 98 % yield.

^{1}H NMR: δ 0.66 (dd, J_1=2.0 Hz, J_2=11.8 Hz, H5"), 0.69 (dd, J_1=1.8 Hz, J_2=11.6 Hz, H5*), 0.73 (s, H27), 0.75 (s, H25*), 0.75 (s, H27"), 0.76 (s, H27*), 0.79 (dd, J_1=1.8 Hz, J_2=11.7 Hz, H5), 0.82 (s, H25), 0.84 (s, H23), 0.85 (s, H23"), 0.87 (s, H24), 0.88 (s, H24*), 0.88 (s, H24"), 0.94 (s, H23*), 0.94 (s, H26"), 0.94 (s, H26), 0.96 (s, H25"), 0.96 (s, H26*), 0.99 (s, H30), 0.99 (s, H30*), 0.99 (s, H30"), 1.00 (s, H29*), 1.00 (s, H29"), 1.00 (s, H29), 1.82 (ddd, J_1=3.7 Hz, J_2=3.7 Hz, J_3=3.7 Hz, H1*), 2.16 (dt, J_1=3.6 Hz, J_2=3.6 Hz, J_3=13.1 Hz, H16*), 2.41 (dt, J_1=3.6 Hz, J_2=3.6 Hz, J_3=13.3 Hz, H16), 2.41 (dt, J_1=3.6 Hz, J_2=3.6 Hz, J_3=13.6 Hz, H16"), 2.97 (dd, J_1=4.2 Hz, J_2=11.5 Hz, H3*), 2.99 (dd, J_1=4.2 Hz, J_2=11.8 Hz, H3"), 3.37-3.44 (m, H1'), 3.37-3.44 (m, H1'"), 3.46-3.52 (m, H1'), 3.46-3.52 (m, H1'"), 4.47 (dd, J_1=5.2 Hz, J_2=11.4 Hz, H3), 4.67 (d, J=13.1 Hz, H11'), 4.67 (d, J=13.1 Hz, H11'"), 4.88 (d, J=13.1 Hz, H11'), 4.88 (d, J=13.1 Hz, H11'"), 5.09 (d, J=12.4 Hz, H1**), 5.12 (d, J=1.4 Hz, H19*), 5.16 (d, J=12.4 Hz, H1**), 5.31 (bs, H19), 5.31 (bs, H19"), 7.27-7.36 (m, H3**), 7.84 (s, H9'"), 7.85 (s, H9').

^{13}C NMR: δ 14.90 (C27), 14.90 (C27"), 14.90 (C27*), 15.80 (C26), 16.00 (C25), 16.00 (C26*), 16.00 (C26"), 16.30 (C24"), 16.50 (C25*), 16.70 (C24*), 16.70 (C25"), 16.80 (C24), 18.10 (C6), 18.10 (C6"), 18.10 (C6*), 20.90 (C11*), 21.00 (C11"), 21.00 (C11), 22.70 (C2*), 22.80 (C2"), 23.60 (C2), 25.90 (C12*), 25.90 (C12"), 26.20 (C12), 27.90 (C23), 28.00 (C23"), 28.10 (C23*), 28.60 (C8"), 28.90 (C30"), 28.90 (C30), 29.10 (C30*), 29.30 (C15"), 29.30 (C15*), 29.30 (C15), 30.40 (C29*), 30.90 (C29"), 30.90 (C29), 32.00 (C20*), 32.30 (C20), 32.30 (C21"), 32.70 (C21), 32.70 (C16"), 32.70 (C21*), 33.20 (C7), 33.20 (C20"), 33.40 (C7*), 33.40 (C16*), 33.50 (C16), 34.00 (C7"), 34.40 (C22*), 34.40 (C22"), 34.50 (C22), 37.10 (C10"), 37.10 (C10), 37.10 (C10*), 37.80 (C8*), 38.60 (C1*), 38.80 (C8), 38.80 (C1"), 38.80 (C1),

39.30 (C1'''), 39.30 (C1'), 40.60 (C14''), 40.70 (C14*), 40.70 (C14), 41.30 (C13*), 41.60 (C13''), 41.60 (C13), 42.50 (C4''), 42.60 (C4*), 42.60 (C4), 48.10 (C17*), 48.20 (C17''), 48.20 (C17), 51.00 (C9), 51.00 (C9*), 51.00 (C9''), 51.90 (C8'''), 51.90 (C8'), 55.60 (C5), 55.70 (C5''), 55.70 (C5*), 61.40 (C11'''), 61.50 (C11'), 66.00 (C1**), 68.80 (C7'), 68.80 (C7'''), 70.30 (C6'''), 70.30 (C6'), 70.50 (C5'''), 70.50 (C4'), 70.50 (C4'''), 70.50 (C5'), 70.60 (C2'), 70.60 (C3'''), 70.60 (C2'''), 70.60 (C3'), 80.80 (C3), 87.20 (C3''), 87.30 (C3*), 125.20 (C9'''), 125.90 (C9'), 127.90 (C5**), 128.00 (C4**), 128.40 (C3**), 132.50 (C19''), 135.60 (C19*), 135.60 (C19), 136.50 (C2**), 136.50 (C18*), 137.10 (C18''), 138.40 (C18), 144.40 (C10'''), 144.50 (C10'), 175.60 (C28*), 175.60 (C28''), 176.40 (C28).

MS (ES): *m/z* = 1979.4 $[M+H]^+$

1.21. (3β)-3-{[1-(2-{2-[2-(2-{[(3β)-3-{[1-(2-{2-[2-(2-{[(3β)-3-(Acetyloxy)-28-oxoolean-18-en-28-yl]amino}ethoxy)ethoxy]ethoxy}ethyl)-1*H*-1,2,3-triazol-4-yl]methoxy}-28-oxoolean-18-en-28-yl]amino}ethoxy)ethoxy]ethoxy}ethyl)-1*H*-1,2,3-triazol-4-yl]methoxy}olean-18-en-28-oic acid (**11b**)

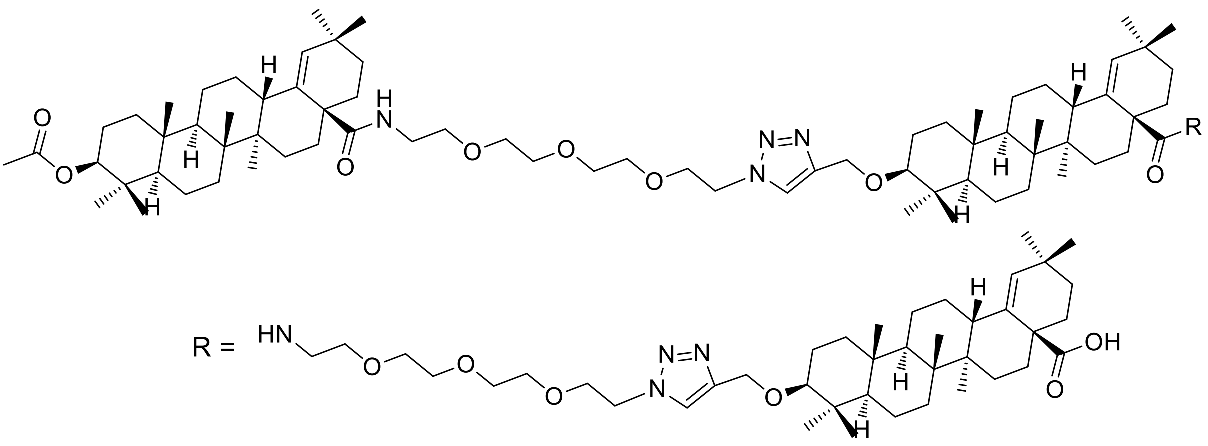

Procedure G was applied, using **10b** as the starting compound, yielding **11b** in an 80 % yield.

¹H NMR: δ 0.68 (dd, J_1=2.0 Hz, J_2=11.5 Hz, H5''), 0.71 (dd, J_1=1.7 Hz, J_2=11.8 Hz, H5*), 0.75 (s, H27''), 0.75 (s, H27), 0.77 (s, H27*), 0.78 (s, H25*), 0.79 (dd, J_1=1.8 Hz, J_2=11.5 Hz, H5), 0.83 (s, H25), 0.84 (s, H23), 0.85 (s, H23''), 0.87 (s, H26''), 0.87 (s, H24), 0.89 (s, H24*), 0.91 (s, H24''), 0.93 (s, H23*), 0.94 (s, H26), 0.94 (s, H25''), 0.97 (s, H26*), 0.98 (s, H30''), 0.98 (s, H30*), 0.98 (s, H30), 0.99 (s, H29), 0.99 (s, H29*), 0.99 (s, H29''), 1.73 (dt, J_1=3.5 Hz, J_2=3.5 Hz, J_3=13.2 Hz, H1), 1.82 (ddd, J_1=3.7 Hz, J_2=4.7 Hz, J_3=14.0 Hz, H1*), 2.17 (dt, J_1=3.5 Hz, J_2=3.5 Hz, J_3=13.4 Hz, H16*), 2.41 (dt, J_1=3.4 Hz, J_2=3.4 Hz, J_3=13.4 Hz, H16), 2.41 (dt,

J_1=3.4 Hz, J_2=3.4 Hz, J_3=13.4 Hz, H16''), 2.97 (dd, J_1=4.2 Hz, J_2=11.6 Hz, H3*), 2.99 (dd, J_1=4.4 Hz, J_2=11.7 Hz, H3''), 3.37-3.44 (m, H1'''), 3.37-3.44 (m, H1'), 3.47-3.52 (m, H1'), 3.47-3.52 (m, H1'''), 3.88 (bt, J=5.3 Hz, H7''), 3.90 (bt, J=5.6 Hz, H7'), 4.47 (dd, J_1=5.2 Hz, J_2=11.3 Hz, H3), 4.58-4.62 (m, H8''), 4.63-4.68 (m, H8'), 4.87 (d, J=12.6 Hz, H11'), 4.87 (d, J=12.6 Hz, H11'''), 4.91 (d, J=12.6 Hz, H11'), 4.91 (d, J=12.6 Hz, H11'''), 5.16 (bd, J=1.4 Hz, H19*), 5.31 (bs, H19''), 5.31 (bs, H19), 7.74 (bs, H9'), 7.84 (bs, H9''').

^{13}C NMR: δ 14.90 (C27), 14.90 (C27*), 14.90 (C27''), 16.00 (C26*), 16.00 (C26''), 16.10 (C26), 16.30 (C25*), 16.50 (C25), 16.50 (C25''), 16.70 (C24*), 16.70 (C24''), 16.80 (C24), 18.10 (C6), 18.10 (C6''), 18.10 (C6*), 20.90 (C11*), 21.00 (C11), 21.00 (C11''), 21.30 (OAc), 22.60 (C2''), 22.70 (C2*), 23.60 (C2), 25.90 (C12*), 26.20 (C12''), 26.30 (C12), 27.90 (C23), 28.10 (C23*), 28.10 (C23''), 28.90 (C30''), 28.90 (C30), 29.10 (C30*), 29.30 (C15''), 29.30 (C15*), 29.40 (C15), 30.40 (C29*), 30.90 (C29), 30.90 (C29''), 32.10 (C20*), 32.30 (C20), 32.30 (C16), 32.30 (C16''), 32.30 (C21''), 32.70 (C21*), 33.20 (C20''), 33.20 (C21), 33.40 (C7*), 33.90 (C7), 33.90 (C16*), 34.40 (C22''), 34.40 (C22), 34.40 (C7''), 34.50 (C22*), 37.10 (C10''), 37.10 (C10), 37.10 (C10*), 37.80 (C8*), 37.80 (C8), 38.50 (C8''), 38.60 (C1*), 38.80 (C1), 38.80 (C1''), 39.30 (C1'''), 39.40 (C1'), 40.70 (C14), 40.70 (C14*), 40.70 (C14''), 41.30 (C13*), 41.60 (C13''), 41.60 (C13), 42.60 (C4), 42.60 (C4''), 42.60 (C4*), 47.90 (C17*), 48.20 (C17), 48.20 (C17''), 51.00 (C9''), 51.00 (C9), 51.00 (C9*), 52.50 (C8'''), 52.60 (C8'), 55.50 (C5''), 55.50 (C5), 55.70 (C5*), 60.80 (C11'''), 61.00 (C11'), 68.50 (C7'''), 68.70 (C7'), 70.30 (C6'''), 70.40 (C4'''), 70.40 (C6'), 70.40 (C5'''), 70.50 (C5'), 70.60 (C3'''), 70.60 (C2'''), 70.60 (C3'), 70.60 (C4'), 70.70 (C2'), 80.80 (C3), 87.50 (C3''), 87.80 (C3*), 125.80 (C9'''), 125.90 (C9'), 133.00 (C19*), 135.60 (C19''), 135.70 (C19), 137.00 (C18*), 138.40 (C18''), 138.40 (C18), 143.60 (C10'''), 143.90 (C10'), 171.10 (OAc), 175.60 (C28''), 175.80 (C28), 178.90 (C28*).

MS (ES): *m/z* = 1894.4 $[M+H]^+$; 1893.0 $[M-H]^-$

1.22. (3β)-3-{[1-(2-{2-[2-(2-{[(3β)-3-{[1-(2-{2-[2-(2-{[(3β)-3-Hydroxy-28-oxoolean-18-en-28-yl]amino}ethoxy)ethoxy]ethoxy}ethyl)-1*H*-1,2,3-triazol-4-yl]methoxy}-28-oxoolean-18-en-28-yl]amino}ethoxy)ethoxy]ethoxy}ethyl)-1*H*-1,2,3-triazol-4-yl]methoxy}olean-18-en-28-oic acid (**12b**)

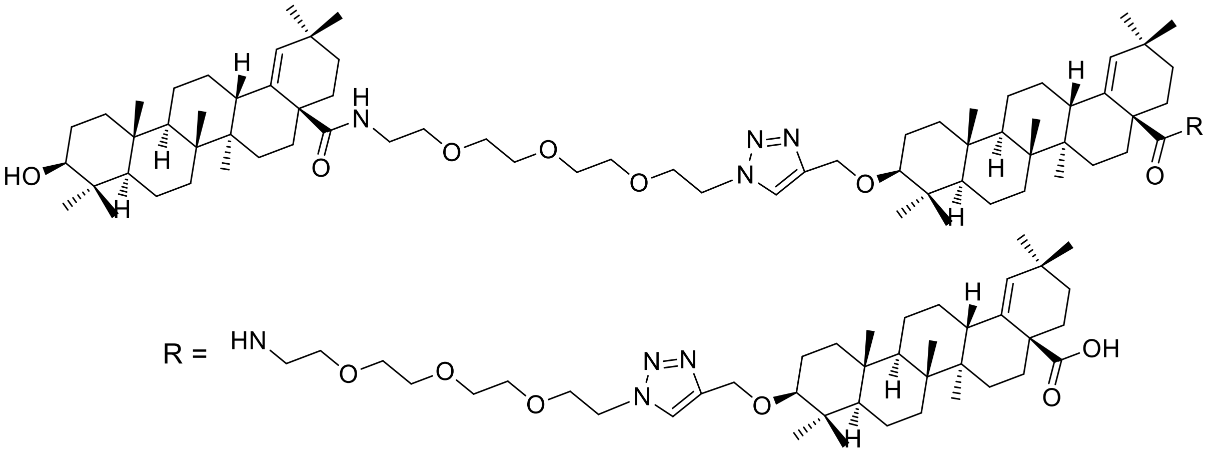

Procedure H was applied, using **11b** as the starting compound, yielding **12b** in a 70 % yield.

^{1}H NMR: δ 0.68 (dd, J_1=1.9 Hz, J_2=11.6 Hz, H5*), 0.68 (dd, J_1=1.9 Hz, J_2=11.6 Hz, H5''), 0.75 (s, H25), 0.75 (s, H27''), 0.75 (s, H27), 0.77 (s, H27*), 0.78 (dd, J_1=2.1 Hz, J_2=11.5 Hz, H5), 0.78 (s, H25*), 0.85 (s, H24), 0.85 (s, H23''), 0.87 (s, H26''), 0.87 (s, H23), 0.90 (s, H24*), 0.91 (s, H24''), 0.94 (s, H23*), 0.94 (s, H25''), 0.94 (s, H26), 0.96 (s, H26*), 0.96 (s, H30*), 0.97 (s, H30''), 0.98 (s, H30), 0.99 (s, H29*), 0.99 (s, H29''), 0.99 (s, H29), 1.73 (dt, J_1=3.3 Hz, J_2=3.3 Hz, J_3=12.8 Hz, H1), 1.80 (dt, J_1=3.5 Hz, J_2=3.5 Hz, J_3=13.5 Hz, H1*), 2.17 (dt, J_1=3.7 Hz, J_2=3.7 Hz, J_3=13.3 Hz, H16*), 2.42 (dt, J_1=3.7 Hz, J_2=3.7 Hz, J_3=13.1 Hz, H16), 2.42 (dt, J_1=3.7 Hz, J_2=3.7 Hz, J_3=13.1 Hz, H16''), 2.97 (dd, J_1=4.4 Hz, J_2=12.1 Hz, H3*), 3.00 (dd, J_1=4.4 Hz, J_2=12.1 Hz, H3''), 3.20 (dd, J_1=4.8 Hz, J_2=11.6 Hz, H3), 3.38-3.44 (m, H1'), 3.38-3.44 (m, H1'''), 3.46-3.53 (m, H1'''), 3.46-3.53 (m, H1'), 3.89 (bt, J=5.4 Hz, H7'), 3.89 (bt, J=5.4 Hz, H7''), 4.58-4.62 (m, H8''), 4.58-4.62 (m, H8'), 4.67 (d, J=13.0 Hz, H11'), 4.67 (d, J=13.0 Hz, H11'''), 4.91 (d, J=13.0 Hz, H11'), 4.91 (d, J=13.0 Hz, H11'''), 5.16 (bs, H19*), 5.30 (bs, H19''), 5.30 (bs, H19), 7.85 (s, H9'''), 7.85 (s, H9').

^{13}C NMR: δ 14.90 (C27), 14.90 (C27''), 14.90 (C27*), 16.00 (C26*), 16.00 (C25), 16.00 (C26''), 16.30 (C26), 16.30 (C25*), 16.40 (C25''), 16.70 (C24''), 16.70 (C24), 16.70 (C24*), 18.10 (C6*), 18.10 (C6''), 18.20 (C6), 20.90 (C11*), 21.00 (C2), 21.00 (C11), 21.00 (C11''), 22.60 (C2''), 22.70 (C2*), 25.90 (C12*), 26.20 (C12''), 26.30 (C12), 27.90 (C23), 28.10 (C23''), 28.10 (C23*), 28.90 (C30), 28.90 (C30''), 29.10 (C30*), 29.30 (C15*), 29.30 (C15''), 29.40 (C15), 30.40 (C29*), 30.90 (C29''), 30.90 (C29), 32.00 (C20), 32.30 (C21''), 32.30 (C20*), 32.30 (C16''), 32.70 (C21), 32.70 (C21*), 33.20 (C7), 33.40 (C20''), 33.40 (C16), 33.50 (C7*), 33.90 (C16*), 34.30 (C22*), 34.40 (C7''), 34.40 (C22''), 34.50 (C22), 37.10 (C10), 37.10 (C10''), 37.10 (C10*), 38.40 (C8*), 38.50 (C8''), 38.80 (C1*), 38.80 (C1''), 38.90 (C1), 38.90

(C8), 39.30 (C1'''), 39.40 (C1'), 40.70 (C14''), 40.70 (C14*), 40.70 (C14), 41.00 (C11'), 41.30 (C13*), 41.60 (C13''), 42.60 (C4*), 42.60 (C4''), 42.60 (C4), 42.60 (C13), 47.90 (C17*), 48.20 (C17''), 48.20 (C17), 51.00 (C9*), 51.00 (C9''), 51.10 (C9), 52.60 (C8'), 52.70 (C8'''), 55.40 (C5), 55.70 (C5*), 55.70 (C5''), 60.80 (C11'''), 68.60 (C7'), 68.60 (C7'''), 70.30 (C6'''), 70.30 (C5'), 70.30 (C6'), 70.30 (C5'''), 70.40 (C4'''), 70.40 (C4'), 70.60 (C2'), 70.60 (C3'''), 70.60 (C3'), 70.70 (C2'''), 78.90 (C3), 87.50 (C3''), 87.80 (C3*), 126.00 (C9'), 126.10 (C9'''), 133.00 (C19*), 135.60 (C19''), 135.70 (C19), 137.00 (C18*), 138.40 (C18''), 138.40 (C18), 143.70 (C10'''), 143.70 (C10'), 175.60 (C28''), 175.80 (C28), 178.80 (C28*).

MS (ES): m/z = 1851.6 [M+H]$^+$; 1949.3 [M-H]$^-$

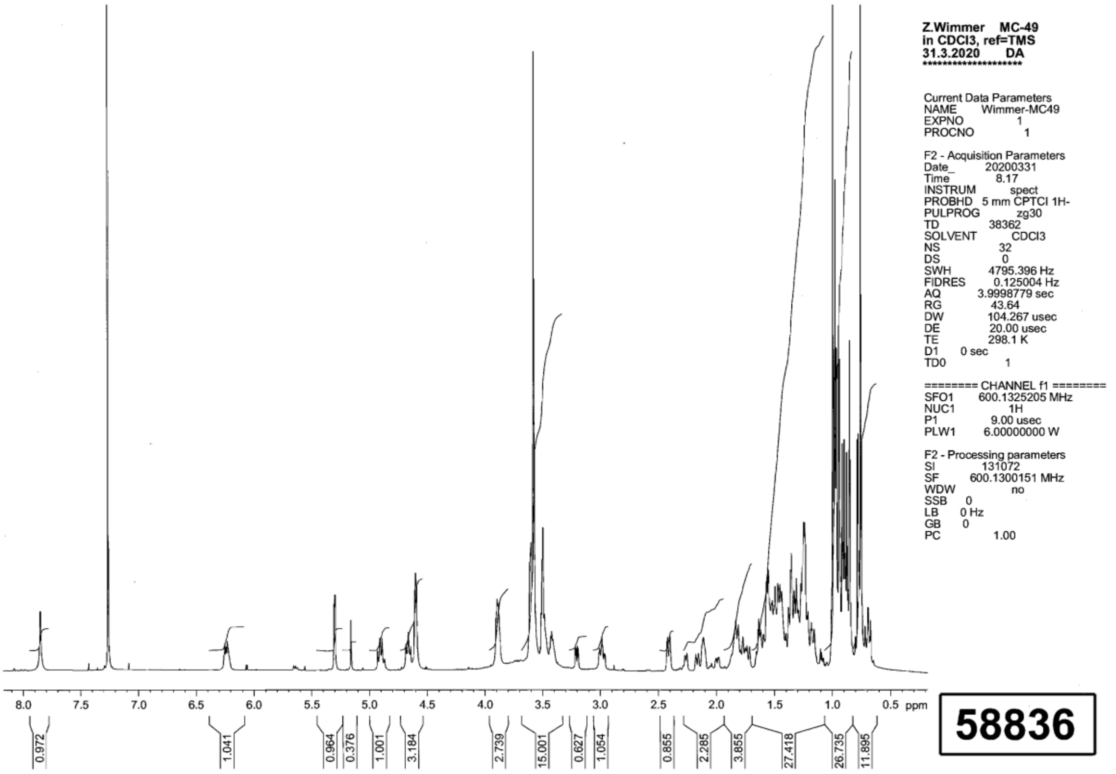

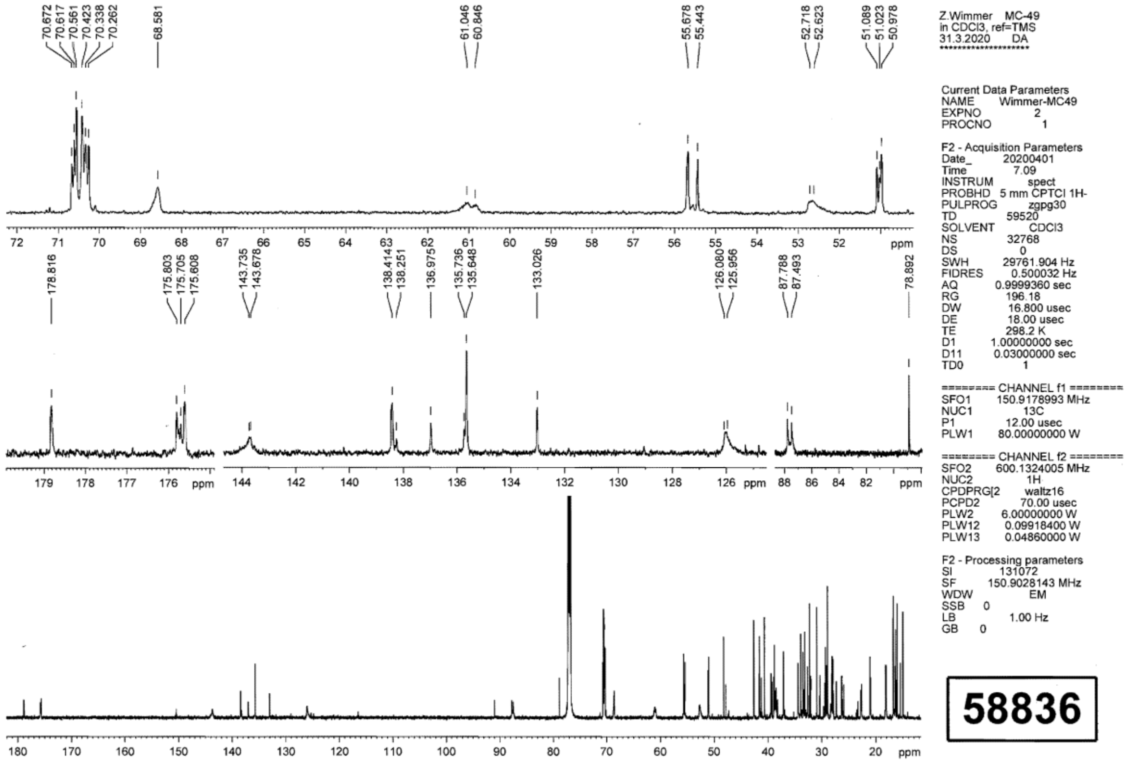

1.23. Prop-2-yn-1-yl-3-oxoolean-18-en-28-oate (**2c**)

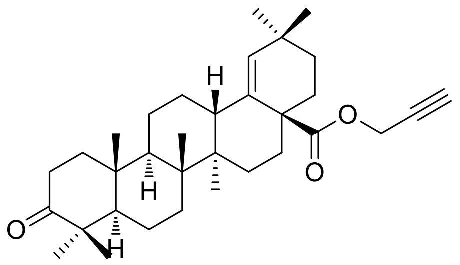

Procedure D was applied, using **1c** as the starting compound, yielding **2c** in an 86 % yield.

^{1}H NMR: δ 0.78 (3H, s, H27), 0.94 (3H, d, J=0.9 Hz, H25), 0.96 (3H, s, H30), 0.99 (3H, s, H29), 1.00 (3H, bs, H26), 1.02 (3H, s, H24), 1.07 (3H, s, H23), 1.22 (2H, dt, J_1=3.3 Hz, J_2=3.3 Hz, J_3=13.3 Hz, H15), 1.25 (2H, dt, J_1=3.8 Hz, J_2=11.5 Hz, J_3=11.5 Hz, H12), 1.64 (2H, dq, J_1=3.0 Hz, J_2=3.0 Hz, J_3=3.0 Hz, J_4=12.2 Hz, H12), 1.94 (2H, ddd, J_1=3.3 Hz, J_2=6.0 Hz, J_3=13.7 Hz, H22), 1.96 (2H, ddd, J_1=4.8 Hz, J_2=7.6 Hz, J_3=13.3 Hz, H1), 2.20 (2H, ddd, J_1=3.0 Hz, J_2=3.8 Hz, J_3=13.7 Hz, H22), 2.20 (1H, ddd, J_1=1.8 Hz, J_2=3.5 Hz, J_3=11.0 Hz, H13), 2.42 (1H, t, J=2.5 Hz, H3'), 2.44 (2H, ddd, J_1=4.8 Hz, J_2=8.0 Hz, J_3=15.7 Hz, H2), 2.49 (2H, ddd,

J_1=7.6 Hz, J_2=9.1 Hz, J_3=15.7 Hz, H2), 4.68 (2H, dd, J_1=2.5 Hz, J_2=15.4 Hz, H1'), 4.70 (2H, dd, J_1=2.5 Hz, J_2=15.4 Hz, H1'), 5.14 (1H, ddd, J_1=0.7 Hz, J_2=1.8 Hz, H19).

^{13}C NMR: δ 14.88 (q, C27), 15.81 (q, C26), 16.51 (q, C25), 19.60 (t, C6), 20.91 (q, C24), 21.49 (t, C11), 25.97 (t, C12), 26.84 (q, C23), 29.09 (q, C30), 29.29 (t, C15), 30.33 (q, C29), 32.01 (t, C21), 33.26 (s, C20), 33.41 (t, C22), 33.41 (t, C16), 33.78 (t, C7), 34.05 (t, C2), 36.89 (s, C10), 39.83 (t, C1), 40.61 (s, C8), 41.37 (d, C13), 42.53 (s, C14), 47.25 (s, C4), 48.10 (s, C17), 50.44 (d, C9), 51.83 (t, C1'), 54.86 (d, C5), 74.28 (d, C3'), 78.08 (s, C2'), 132.95 (d, C19), 136.60 (s, C18), 175.77 (s, C28), 218.25 (s, C3).

MS (ES): *m/z* = 493.2 $[M+H]^+$

1.24. *N*-(2-{2-[2-(2-Azidoethoxy)ethoxy]ethoxy}ethyl)-3-oxoolean-18-en-28-amide (**3c**)

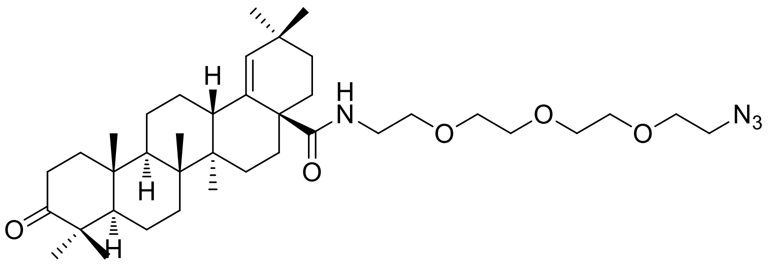

Procedure E was applied, using **1c** as the starting compound, yielding **3c** in a 90 % yield.

^{1}H NMR: δ 0.77 (3H, d, H27), 0.94 (3H, s, J=0.8 Hz, H25), 0.97 (3H, s, H30), 0.97 (3H, bs, H29), 1.00 (3H, s, H26), 1.02 (3H, ddd, H24), 1.07 (3H, ddd, H23), 1.19 (2H, dq, J_1=3.5 Hz, J_2=3.5 Hz, J_3=13.0 Hz, H15), 1.84 (2H, ddd, J_1=3.4 Hz, J_2=5.0 Hz, J_3=13.8 Hz, H22), 1.95 (2H, ddd, J_1=5.0 Hz, J_2=7.5 Hz, J_3=13.0 Hz, H1), 2.15 (1H, dt, J_1=1.7 Hz, J_2=3.4 Hz, J_3=11.9 Hz, H13), 2.43 (2H, ddd, J_1=3.0 Hz, J_2=3.4 Hz, J_3=13.5 Hz, H16), 2.45 (2H, ddd, J_1=5.0 Hz, J_2=8.2 Hz, J_3=15.7 Hz, H2), 2.49 (2H, ddd, J_1=7.5 Hz, J_2=9.0 Hz, J_3=15.7 Hz, H2), 3.36-3.40 (2H, m, H8'), 3.41-3.53 (2H, m, H1'), 3.58-3.69 (2H, m, H5'), 3.58-3.69 (2H, m, H6'), 3.58-3.69 (2H, m, H7'), 3.58-3.69 (2H, m, H3'), 3.58-3.69 (2H, m, H4'), 3.58-3.69 (2H, m, H2'), 5.31 (1H, dt, J_1=0.8 Hz, J_2=1.7 Hz, H19), 6.25 (0H, 5.9, H1'-NH).

^{13}C NMR: δ 14.80 (q, C27), 15.85 (q, C26), 16.56 (q, C25), 19.59 (t, C6), 20.88 (q, C24), 21.56 (t, C11), 24.22 (t, C12), 26.86 (q, C23), 28.96 (q, C30), 29.29 (t, C15), 30.86 (q, C29), 32.28 (t, C21), 32.63 (t, C16), 33.17 (s, C20), 33.66 (t, C22), 33.92 (t, C7), 34.00 (t, C2), 36.87 (s, C10), 39.35 (t, C1'), 39.82 (t, C1), 40.55 (s, C8), 41.63 (d, C13), 42.62 (s, C14), 47.23 (s, C4),

48.19 (s, C17), 50.40 (d, C9), 50.65 (t, C8'), 54.80 (d, C5), 70.06 (t, C7'), 70.33 (t, C6'), 70.36 (t, C5'), 70.55 (t, C4'), 70.62 (t, C3'), 70.69 (t, C2'), 135.69 (d, C19), 138.32 (s, C18), 175.47 (s, C28), 218.17 (s, C3).

MS (ES): *m/z* = 655.3 $[M+H]^+$; 653.2 $[M-H]^-$

1.25. [1-(2-{2-[2-(2-{[3,28-Dioxoolean-18-en-28-yl]amino}ethoxy)ethoxy]ethoxy}ethyl)-1*H*-1,2,3-triazol-4-yl]methyl 3-oxoolean-18-en-28-oate (**4c**)

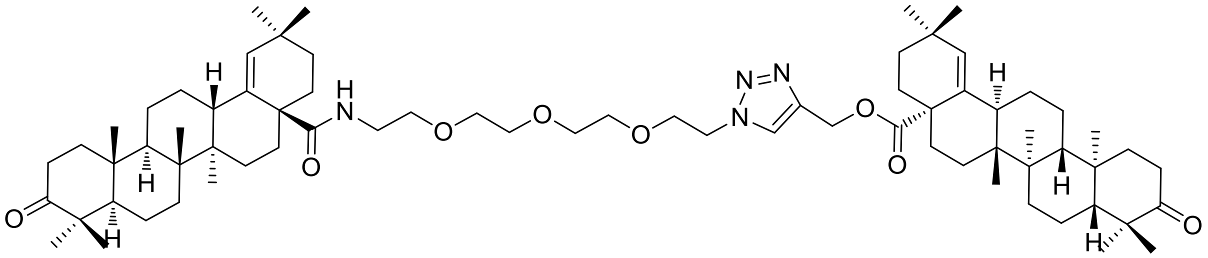

Procedure F was applied, using **2c** and **3c** as the starting compound, yielding **4c** in a 99 % yield.

¹H NMR: δ 0.77 (3H, s, H27''), 0.79 (3H, s, H26), 0.81 (3H, s, H25), 0.94 (3H, s, H25''), 0.96 (3H, s, H29''), 0.96 (3H, s, H24), 0.97 (3H, s, H30''), 1.00 (3H, s, H26''), 1.00 (3H, s, H29), 1.02 (3H, s, H27), 1.04 (3H, s, H23), 1.04 (3H, s, H24''), 1.08 (3H, s, H23''), 1.10 (3H, s, H22), 1.86 (2H, ddd, J_1=3.5 Hz, J_2=4.7 Hz, J_3=13.9 Hz, H22''), 1.89 (2H, dt, J_1=4.6 Hz, J_2=4.6 Hz, J_3=13.8 Hz, H19), 1.97 (2H, ddd, J_1=4.8 Hz, J_2=7.2 Hz, J_3=12.7 Hz, H1''), 1.98 (2H, ddd, J_1=4.8 Hz, J_2=7.2 Hz, J_3=12.7 Hz, H30), 2.43-2.48 (2H, m, H16''), 2.43-2.48 (2H, m, H1), 3.40-3.46 (2H, m, H1'), 3.50-3.63 (2H, m, H3'), 3.50-3.63 (2H, m, H5'), 3.50-3.63 (2H, m, H2'), 3.50-3.63 (2H, m, H6'), 3.50-3.55 (2H, m, H1'), 3.50-3.63 (2H, m, H4'), 3.88 (2H, t, *J*=5.4 Hz, H7'), 4.54 (2H, ddd, J_1=4.6 Hz, J_2=5.4 Hz, H8'), 5.13 (1H, bd, *J*=1.5 Hz, H16), 5.23 (2H, s, H11'), 5.33 (1H, bs, H19''), 6.24 (1H, t, *J*=5.6 Hz, H1'-NH), 7.78 (1H, s, H9').

¹³C NMR: δ 14.83 (q, C27), 14.87 (q, C27''), 15.41 (q, C26''), 15.89 (q, C26), 16.55 (q, C25), 16.61 (q, C25''), 19.60 (t, C6), 19.62 (t, C6''), 20.91 (q, C24), 20.93 (q, C24''), 21.45 (t, C11''), 21.59 (t, C11), 25.91 (t, C12''), 26.28 (t, C12), 26.86 (q, C23), 26.91 (q, C23''), 28.97 (q, C30), 29.06 (q, C30''), 29.28 (t, C15), 29.31 (t, C15''), 30.43 (q, C29''), 30.93 (q, C29), 32.01 (t, C21''), 32.31 (t, C21), 32.64 (t, C16''), 32.64 (t, C16), 33.21 (s, C20), 33.25 (s, C20''), 33.37 (t, C22), 33.43 (t, C22''), 33.68 (t, C7), 33.68 (t, C7''), 34.02 (t, C2), 34.03 (t, C2''), 36.88 (s, C10), 36.90 (s, C10''), 39.37 (t, C1'), 39.81 (t, C1), 39.86 (t, C1''), 40.46 (s, C8''), 40.58 (s, C8), 41.31 (d,

C13''), 41.68 (d, C13), 42.47 (s, C14''), 42.67 (s, C14), 47.07 (s, C17''), 47.24 (s, C4), 47.25 (s, C4''), 48.23 (s, C17), 50.37 (t, C8'), 50.37 (d, C9''), 50.42 (d, C9), 54.81 (d, C5), 54.81 (d, C5''), 57.41 (t, C11'), 69.38 (t, C7'), 70.30 (t, C5'), 70.32 (t, C6'), 70.51 (t, C4'), 70.54 (t, C3'), 70.57 (t, C2'), 125.17 (d, C9'), 132.88 (d, C19''), 135.68 (d, C19), 136.72 (s, C18''), 138.40 (s, C18), 142.85 (s, C10'), 175.50 (s, C28), 176.55 (s, C28''), 218.12 (s, C3), 218.36 (s, C3'').

MS (ES): *m/z* = 1147.7 $[M+H]^+$; 1145.7 $[M-H]^-$

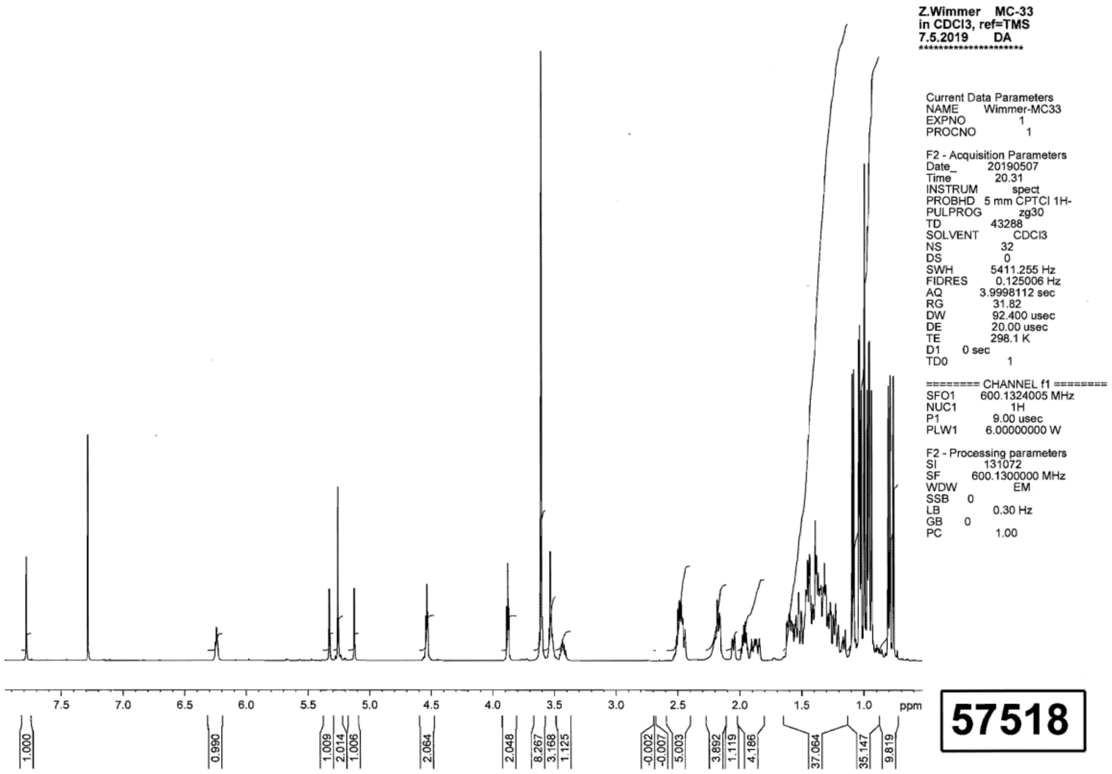

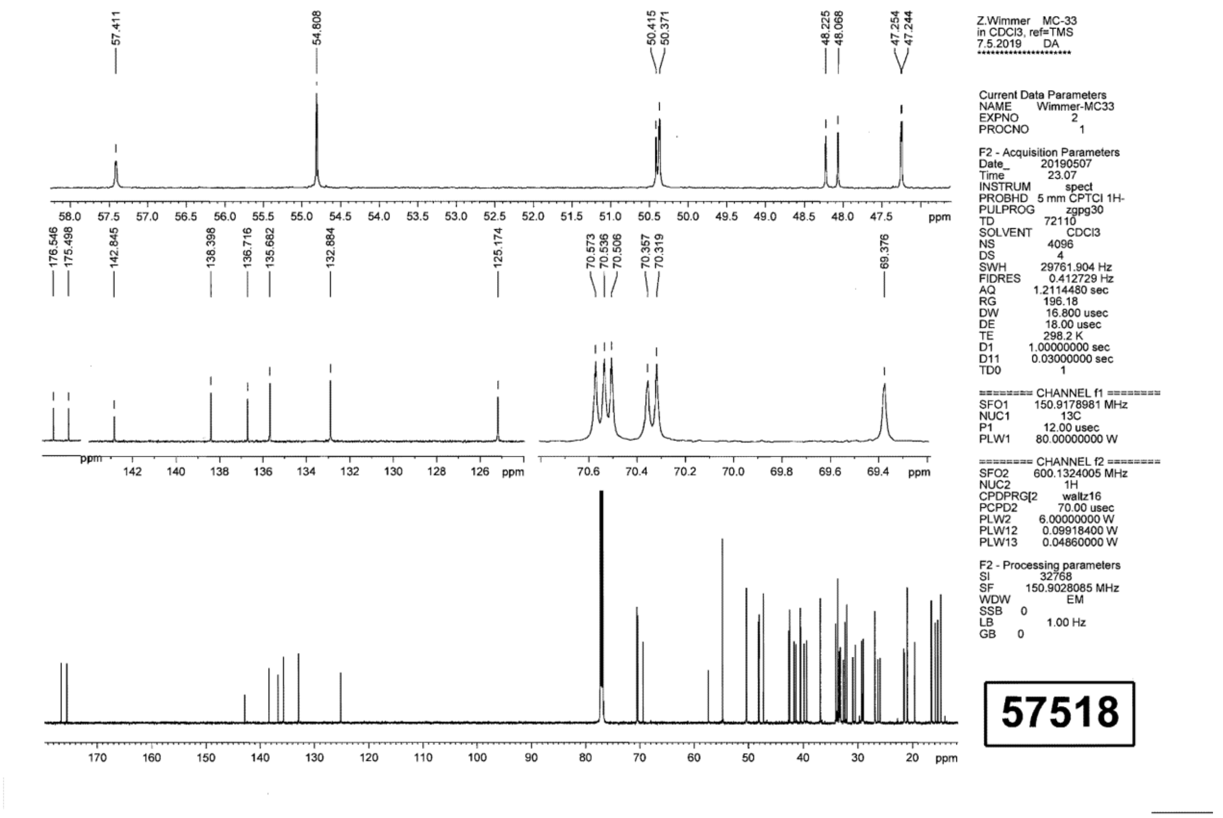

1.26. Benzyl (3β)-3-{[1-(2-{2-[2-(2-{[3,28-dioxoolean-18-en-28-yl]amino}ethoxy)ethoxy]-ethoxy}ethyl)-1*H*-1,2,3-triazol-4-yl]methoxy}olean-12-en-28-oate (**5c**)

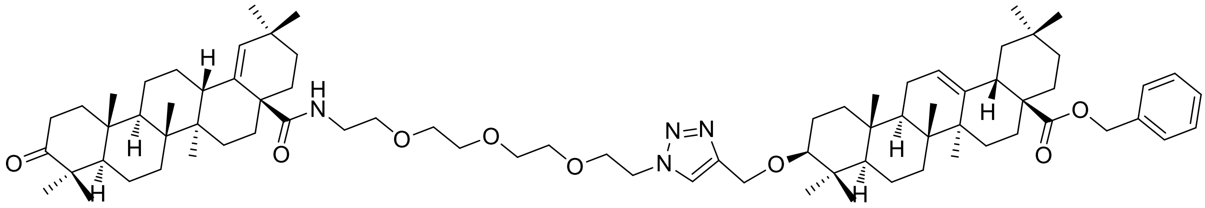

Procedure F was applied, using **3c** and **1a** as the starting compounds, yielding **5c** in a 99 % yield.

^{1}H NMR: δ 0.59 (3H, s, H26"), 0.71 (1H, dd, J_1=1.9 Hz, J_2=11.7 Hz, H5"), 0.77 (3H, s, H27), 0.77 (3H, s, H25"), 0.87 (3H, s, H24"), 0.90 (3H, s, H29"), 0.90 (3H, s, H23"), 0.91 (3H, s, H30"), 0.93 (3H, d, *J*=0.5 Hz, H25), 0.97 (3H, s, H26), 0.97 (3H, s, H30), 1.00 (3H, s, H29), 1.02 (3H, s, H24), 1.07 (3H, s, H23), 1.11 (3H, d, *J*=0.6 Hz, H27"), 1.81-1.86 (2H, m, H2"), 1.83 (2H, ddd, J_1=3.3 Hz, J_2=5.0 Hz, J_3=13.0 Hz, H22), 1.95 (2H, ddd, J_1=5.1 Hz, J_2=7.4 Hz, J_3=12.8 Hz, H1), 2.13-2.13 (2H, m, H13), 2.43-2.52 (3H, m, H2), 2.90 (2H, bdd, J_1=4.2 Hz, J_2=13.9 Hz, H18"), 2.98 (1H, bdd, J_1=4.3 Hz, J_2=11.7 Hz, H3"), 3.37-3.43 (2H, m, H1'), 3.48-

3.62 (2H, m, H5'), 3.48-3.62 (2H, m, H6'), 3.48-3.52 (2H, m, H1'), 3.48-3.62 (2H, m, H4'), 3.48-3.62 (2H, m, H2'), 3.48-3.62 (2H, m, H3'), 3.88 (2H, t, *J*=5.2 Hz, H7'), 4.55-4.58 (2H, m, H8'), 4.62 (2H, d, *J*=12.9 Hz, H11'), 4.83 (2H, d, *J*=12.9 Hz, H11'), 5.04 (2H, d, *J*=12.6 Hz, H1*), 5.09 (2H, d, *J*=12.6 Hz, H1*), 5.28 (2H, t, *J*=3.8 Hz, H12''), 5.30 (2H, dd, J_1=0.7 Hz, J_2=1.8 Hz, H19), 7.28-7.40 (1H, m, H4*), 7.28-7.40 (1H, m, H5*), 7.28-7.40 (1H, m, H3*), 7.77 (1H, s, H9').

^{13}C NMR: δ 14.80 (q, C27), 15.31 (q, C24''), 15.86 (q, C26), 16.47 (q, C25''), 16.58 (q, C25), 16.87 (q, C26''), 18.23 (s, C6''), 19.58 (t, C6), 20.88 (q, C24), 21.55 (t, C11), 22.39 (d, C2''), 22.59 (t, C16''), 23.02 (t, C11''), 23.62 (q, C30''), 25.86 (q, C27''), 26.25 (t, C12), 26.86 (q, C23), 27.59 (t, C15''), 28.16 (q, C23''), 28.93 (q, C30), 29.27 (t, C15), 30.30 (q, C29), 30.68 (s, C20''), 32.28 (t, C21), 32.35 (t, C22''), 32.66 (d, C7''), 33.08 (q, C29''), 33.17 (s, C20), 33.65 (t, C22), 33.65 (t, C7), 33.83 (t, C21''), 33.93 (t, C16), 33.98 (t, C2), 36.86 (s, C10), 36.92 (s, C10''), 38.14 (t, C1''), 38.67 (t, C4''), 39.28 (s, C8''), 39.35 (t, C1'), 39.83 (t, C1), 40.55 (s, C8), 41.35 (d, C18''), 41.65 (s, C14''), 41.65 (d, C13), 42.63 (s, C14), 45.82 (t, C19''), 46.72 (s, C17''), 47.22 (s, C4), 47.51 (d, C9''), 48.20 (s, C17), 50.38 (d, C9), 51.16 (t, C8'), 54.78 (d, C5), 55.50 (t, C5''), 62.22 (t, C11'), 65.89 (t, C1*), 69.16 (t, C7'), 70.13 (t, C5'), 70.27 (t, C6'), 70.44 (t, C4'), 70.50 (t, C3'), 70.56 (t, C2'), 86.99 (s, C3''), 122.45 (d, C12''), 124.36 (d, C9'), 127.87 (d, C5*), 127.93 (d, C4*), 128.38 (d, C3*), 135.67 (d, C19), 136.41 (s, C2*), 138.34 (s, C18), 143.65 (s, C13''), 145.18 (s, C10'), 175.52 (s, C28), 177.45 (s, C28''), 218.14 (s, C3).

MS (ES): *m/z* = 1240.4 $[M+H]^+$; 1238.8 $[M-H]^-$

1.27. Benzyl (3β)-3-{[1-(2-{2-[2-(2-{[3,28-dioxoolean-18-en-28-yl]amino}ethoxy)ethoxy]-ethoxy}ethyl)-1*H*-1,2,3-triazol-4-yl]methoxy}olean-18-en-28-oate (**5d**)

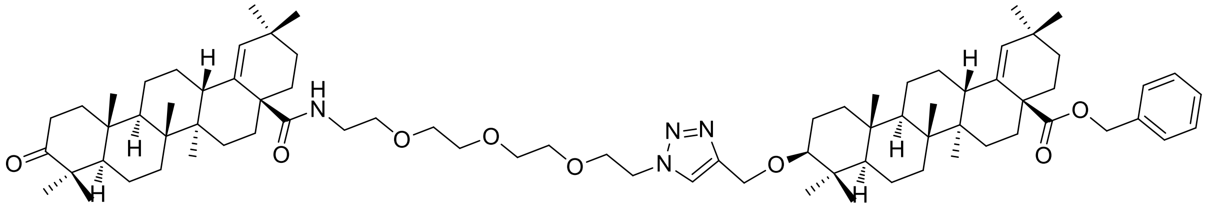

Procedure F was applied, using **3c** and **1b** as the starting compounds, yielding **5d** in a 99 % yield.

^{1}H NMR: δ 0.69 (1H, dd, J_1=1.9 Hz, J_2=11.2 Hz, H5"), 0.76 (3H, s, H27), 0.77 (3H, s, H26"), 0.77 (3H, s, H25"), 0.79 (3H, s, H27"), 0.85 (3H, s, H24"), 0.91 (3H, s, H23"), 0.96 (3H, d, J=0.6 Hz, H25), 0.98 (3H, s, H29"), 0.99 (3H, s, H26), 1.00 (3H, s, H30), 1.00 (3H, s, H30"), 1.02 (3H, s, H29), 1.04 (3H, s, H24), 1.09 (3H, s, H23), 1.78 (2H, dt, J_1=3.7 Hz, J_2=3.7 Hz, J_3=13.2 Hz, H1"), 1.85 (2H, ddd, J_1=3.5 Hz, J_2=5.0 Hz, J_3=13.9 Hz, H22), 1.94 (2H, ddd, J_1=3.3 Hz, J_2=6.1 Hz, J_3=13.8 Hz, H7"), 1.97 (2H, ddd, J_1=5.2 Hz, J_2=7.3 Hz, J_3=13.0 Hz, H1), 2.05 (1H, ddd, J_1=1.7 Hz, J_2=3.5 Hz, J_3=11.9 Hz, H13"), 2.16 (1H, ddd, J_1=1.8 Hz, J_2=3.3 Hz, J_3=11.5 Hz, H13), 2.18 (2H, ddd, J_1=3.2 Hz, J_2=3.7 Hz, J_3=13.5 Hz, H16"), 2.47 (2H, ddd, J_1=5.6 Hz, J_2=8.4 Hz, J_3=15.7 Hz, H2), 2.51 (2H, ddd, J_1=7.3 Hz, J_2=8.4 Hz, J_3=15.7 Hz, H2), 3.01 (1H, dd, J_1=4.4 Hz, J_2=11.8 Hz, H3"), 3.28-3.43 (2H, m, H1'), 3.52-3.65 (2H, m, H6'), 3.52-3.65 (2H, m, H4'), 3.52-3.65 (2H, m, H5'), 3.52-3.65 (2H, m, H3'), 3.52-3.55 (2H, m, H1'), 3.52-3.65 (2H, m, H2'), 3.94 (2H, t, J=5.2 Hz, H7'), 4.64-4.66 (2H, m, H8'), 4.73 (2H, d, J=13.4 Hz, H11'), 4.95 (2H, d, J=13.4 Hz, H11'), 5.11 (2H, d, J=12.4 Hz, H1*), 5.14 (1H, dd, J_1=0.6 Hz, J_2=1.8 Hz, H19"), 5.19 (2H, d, J=12.4 Hz, H1*), 5.33 (1H, dd, J_1=0.6 Hz, J_2=1.8 Hz, H19), 7.29-7.37 (1H, m, H4*), 7.29-7.37 (1H, m, H5*), 7.29-7.37 (1H, m, H3*), 7.93 (1H, s, H9').

^{13}C NMR: δ 14.81 (q, C27), 14.92 (q, C27"), 15.81 (q, C26"), 15.86 (q, C26), 16.28 (qq, C25"), 16.59 (q, C25), 16.65 (q, C24"), 18.10 (t, C6"), 19.58 (t, C6), 20.88 (t, C11"), 20.88 (q, C24), 21.56 (t, C11), 22.72 (t, C2"), 25.91 (t, C12"), 26.26 (t, C12), 26.88 (q, C23), 28.07 (q, C23"), 28.93 (q, C30), 29.14 (q, C30"), 29.27 (t, C15), 29.27 (t, C15"), 30.34 (q, C29"), 30.91 (q, C29), 31.98 (s, C20"), 32.29 (t, C21), 32.61 (t, C21"), 33.17 (s, C20), 33.34 (t, C7"), 33.42 (t, C7), 33.45 (t, C16"), 33.65 (t, C22), 33.94 (t, C16), 33.98 (t, C2), 34.47 (t, C22"), 36.87 (s, C10), 37.10 (s, C10"), 38.51 (t, C1"), 38.80 (s, C8"), 39.38 (t, C1'), 39.83 (t, C1), 40.55 (s, C8), 40.59 (s, C14"), 41.29 (d, C13"), 41.67 (d, C13), 42.47 (s, C4"), 42.64 (s, C14), 47.23 (s, C4), 48.13 (s, C17"), 48.20 (s, C17), 50.38 (d, C9), 51.01 (d, C9"), 52.33 (t, C8'), 54.77 (d, C5), 55.70 (d, C5"), 61.11 (t, C11'), 66.02 (t, C1*), 68.63 (t, C7'), 70.24 (t, C6'), 70.30 (t, C5'), 70.43 (t, C4'), 70.54 (t, C3'), 70.60 (t, C2'), 87.58 (d, C3"), 125.55 (d, C9'), 127.92 (d, C5*), 128.01 (d, C4*), 128.41 (d, C3*), 132.54 (d, C19"), 135.71 (d, C19), 136.48 (s, C2*), 137.01 (s, C18"), 138.32 (s, C18), 144.00 (s, C10'), 175.59 (s, C28), 176.40 (s, C28"), 218.13 (s, C3).

MS (ES): *m/z* = 1240.4 $[M+H]^+$; 1238.7 $[M-H]^-$

1.28. (3β)-3-{[1-(2-{2-[2-(2-{[3,28-Dioxoolean-18-en-28-yl]amino}ethoxy)ethoxy]ethoxy}-ethyl)-1*H*-1,2,3-triazol-4-yl]methoxy}olean-12-en-28-oic acid (**6c**)

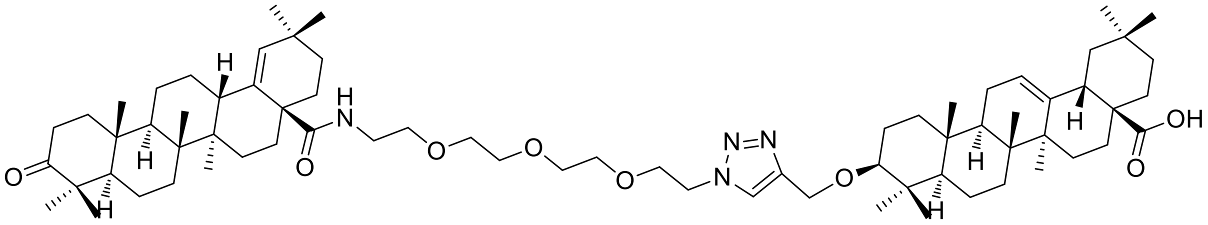

Procedure G was applied, using **5c** as the starting compound, yielding **6c** in a 93 % yield.

^{1}H NMR: δ 0.73 (1H, dd, J_1=1.8 Hz, J_2=11.5 Hz, H5''), 0.74 (3H, s, H26''), 0.76 (3H, s, H27), 0.77 (3H, s, H25''), 0.90 (3H, s, H24''), 0.90 (3H, s, H29''), 0.91 (3H, s, H23''), 0.92 (3H, s, H30''), 0.93 (3H, d, *J*=0.7 Hz, H25), 0.97 (3H, s, H30), 0.97 (3H, s, H26), 0.99 (3H, s, H29), 1.02 (3H, s, H24), 1.07 (3H, s, H23), 1.12 (3H, s, H27''), 1.82-1.90 (2H, m, H2''), 1.83 (2H, ddd, J_1=3.5 Hz, J_2=4.8 Hz, J_3=13.8 Hz, H22), 1.94 (2H, ddd, J_1=5.1 Hz, J_2=7.5 Hz, J_3=12.8 Hz, H1), 2.12-2.17 (1H, m, H13), 2.44-2.53 (2H, m, H2), 2.82 (1H, dd, J_1=4.2 Hz, J_2=14.0 Hz, H18''), 3.00 (1H, dd, J_1=4.3 Hz, J_2=11.7 Hz, H3''), 3.36-3.42 (2H, m, H1'), 3.47-3.52 (2H, m, H1'), 3.49-3.62 (2H, m, H3'), 3.49-3.62 (2H, m, H2'), 3.49-3.62 (2H, m, H5'), 3.49-3.62 (2H, m, H4'), 3.49-3.62 (2H, m, H6'), 3.90 (2H, t, *J*=5.1 Hz, H7'), 4.59-4.62 (2H, m, H8'), 4.67 (2H, d, *J*=13.2 Hz, H11'), 4.88 (2H, d, *J*=13.2 Hz, H11'), 5.03 (1H, t, *J*=3.8 Hz, H12''), 5.30 (1H, dd, J_1=0.7 Hz, J_2=1.8 Hz, H19), 7.85 (1H, s, H9').

^{13}C NMR: δ 14.80 (q, C27), 15.33 (q, C24''), 15.86 (q, C26), 16.47 (q, C25''), 16.61 (q, C25), 17.09 (q, C26''), 18.22 (t, C6''), 19.58 (t, C6), 20.86 (q, C24), 21.56 (t, C11), 22.58 (t, C16''), 22.93 (t, C11''), 23.39 (t, C2''), 23.55 (q, C30''), 25.90 (q, C27''), 26.27 (t, C12), 26.89 (q, C23), 27.65 (st, C15''), 28.19 (q, C23''), 28.93 (q, C30), 29.27 (t, C15), 30.66 (s, C20''), 30.90 (q, C29), 32.29 (t, C21), 32.40 (t, C22), 32.40 (t, C22''), 32.60 (t, C7''), 33.05 (q, C29''), 33.17 (s, C20), 33.65 (t, C7), 33.78 (t, C21''), 33.94 (t, C16), 33.94 (t, C2), 36.86 (s, C10), 36.98 (s, C10''), 38.06 (t, C1''), 38.69 (s, C4''), 39.26 (s, C8''), 39.35 (t, C1'), 39.87 (t, C1), 40.54 (s, C8), 41.03 (d, C18''), 41.60 (s, C14''), 41.66 (d, C13), 42.63 (s, C14), 45.77 (d, C5), 45.84 (t, C19''), 46.43 (s, C17''), 47.24 (s, C4), 47.51 (d, C9''), 48.19 (s, C17), 50.38 (d, C9), 51.59 (t, C8'), 55.48 (d, C5''), 61.79 (t, C11'), 68.96 (t, C7'), 70.28 (t, C6'), 70.31 (t, C5'), 70.43 (t, C4'), 70.51 (t, C3'), 70.57 (t, C2'), 87.19 (d, C3''), 122.52 (d, C12''), 124.76 (d, C9'), 135.69 (d, C19), 138.33 (s, C18), 143.60 (s, C13''), 144.73 (s, C10'), 175.56 (s, C28), 181.96 (s, C28''), 218.48 (s, C3).

MS (ES): *m/z* = 1150.1 $[M+H]^+$; 1148.1 $[M-H]^-$

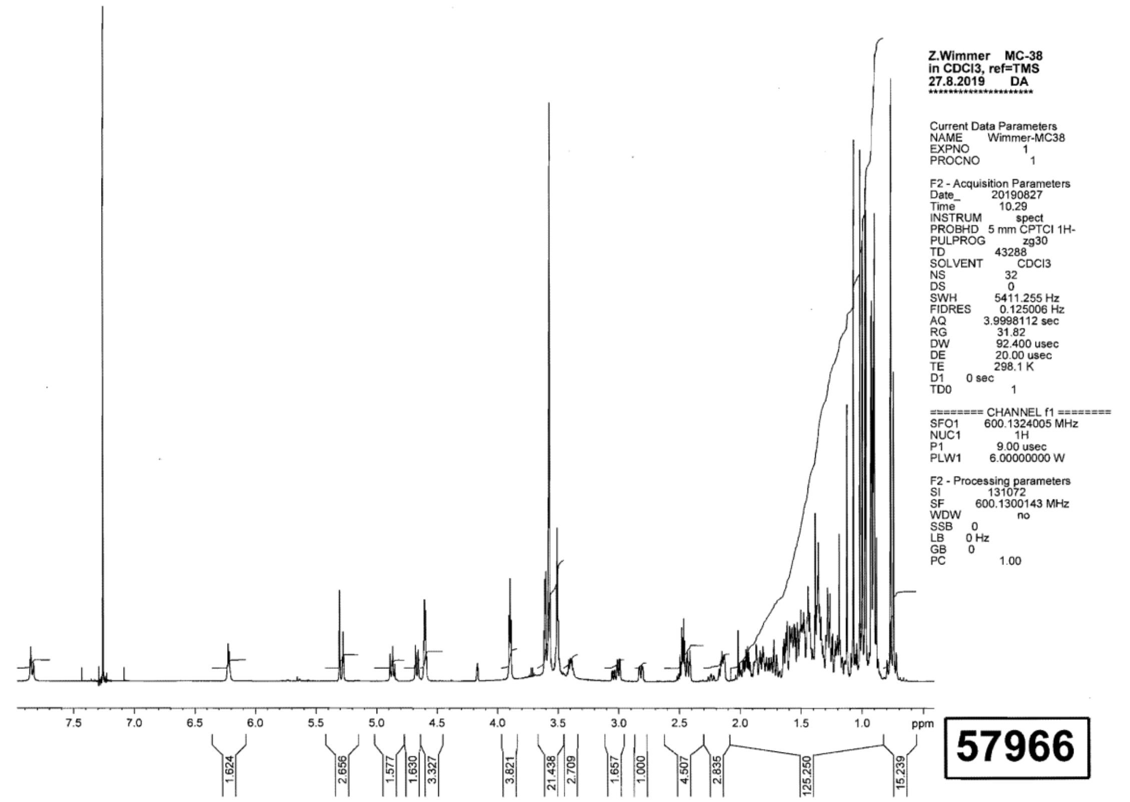
Z.Wimmer MC-38
in CDCl3, ref=TMS
27.8.2019 DA
Current Data Parameters
NAME Wimmer-MC38
EXPNO 1
PROCNO 1
F2 - Acquisition Parameters
Date_ 20190827
Time 10.29
INSTRUM spect
PROBHD 5 mm CPTCI 1H-
PULPROG zg30
TD 43288
SOLVENT CDCl3
NS 32
DS 0
SWH 5411.255 Hz
FIDRES 0.125006 Hz
AQ 3.9998112 sec
RG 31.82
DW 92.400 usec
DE 20.00 usec
TE 298.1 K
D1 0 sec
TD0 1
======== CHANNEL f1 ========
SFO1 600.1324005 MHz
NUC1 1H
P1 9.00 usec
PLW1 6.00000000 W
F2 - Processing parameters
SI 131072
SF 600.1300143 MHz
WDW no
SSB 0
LB 0 Hz
GB 0
PC 1.00
7.5 7.0 6.5 6.0 5.5 5.0 4.5 4.0 3.5 3.0 2.5 2.0 1.5 1.0 ppm
1.624 2.656 1.577 1.630 3.327 3.821 21.438 2.709 1.657 1.000 4.507 2.835 125.250 15.239
57966

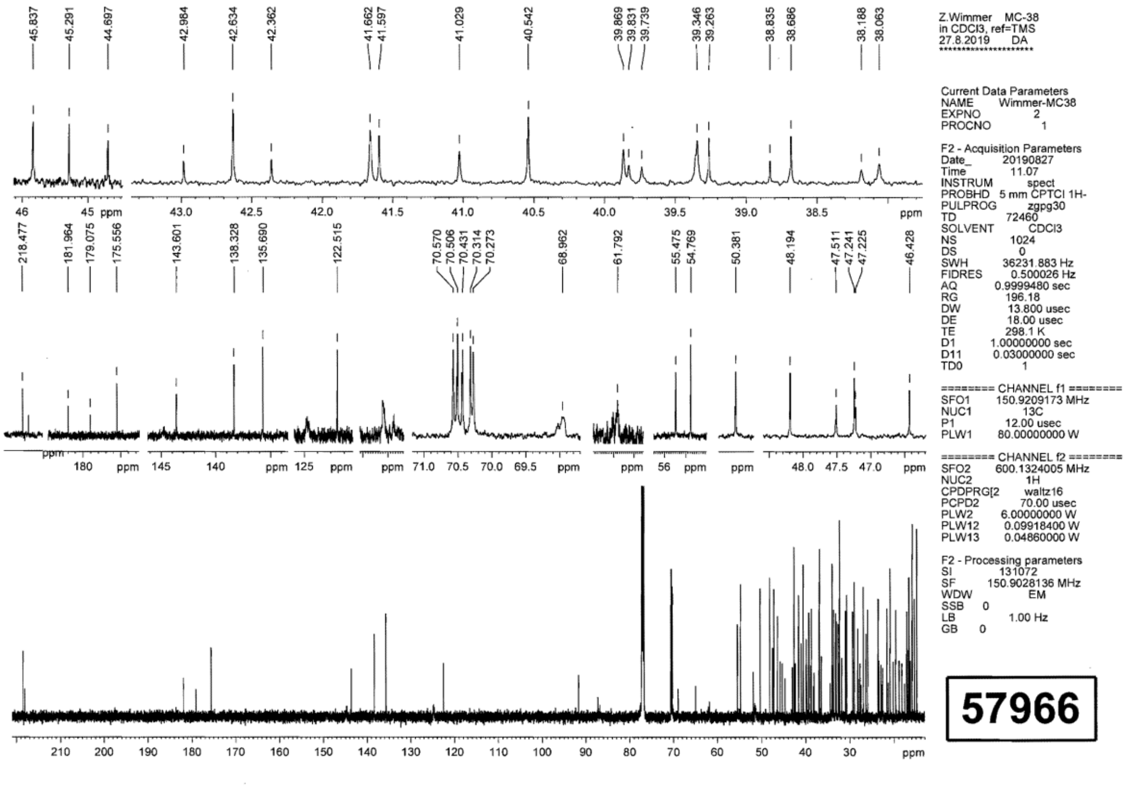
45.837 45.291 44.697 42.984 42.634 42.362 41.662 41.597 41.029 40.542 39.869 39.831 39.739 39.346 39.263 38.835 38.686 38.188 38.063
46 45 ppm 43.0 42.5 42.0 41.5 41.0 40.5 40.0 39.5 39.0 38.5 ppm
218.477 181.964 179.075 175.556 143.601 138.328 135.690 122.515 70.570 70.506 70.431 70.314 70.273 68.962 61.792 55.475 54.769 50.381 48.194 47.511 47.241 47.225 46.428
ppm 180 ppm 145 140 ppm 125 ppm ppm 71.0 70.5 70.0 69.5 ppm ppm 56 ppm ppm 48.0 47.5 47.0 ppm
Z.Wimmer MC-38
in CDCl3, ref=TMS
27.8.2019 DA
Current Data Parameters
NAME Wimmer-MC38
EXPNO 2
PROCNO 1
F2 - Acquisition Parameters
Date_ 20190827
Time 11.07
INSTRUM spect
PROBHD 5 mm CPTCI 1H-
PULPROG zgpg30
TD 72460
SOLVENT CDCl3
NS 1024
DS 0
SWH 36231.883 Hz
FIDRES 0.500026 Hz
AQ 0.9999480 sec
RG 196.18
DW 13.800 usec
DE 18.00 usec
TE 298.1 K
D1 1.00000000 sec
D11 0.03000000 sec
TD0 1
======== CHANNEL f1 ========
SFO1 150.9209173 MHz
NUC1 13C
P1 12.00 usec
PLW1 80.00000000 W
======== CHANNEL f2 ========
SFO2 600.1324005 MHz
NUC2 1H
CPDPRG[2 waltz16
PCPD2 70.00 usec
PLW2 6.00000000 W
PLW12 0.09918400 W
PLW13 0.04860000 W
F2 - Processing parameters
SI 131072
SF 150.9028136 MHz
WDW EM
SSB 0
LB 1.00 Hz
GB 0
210 200 190 180 170 160 150 140 130 120 110 100 90 80 70 60 50 40 30 ppm
57966

1.29. (3β)-3-{[1-(2-{2-[2-(2-{[3,28-Dioxoolean-18-en-28-yl]amino}ethoxy)ethoxy]ethoxy}-ethyl)-1*H*-1,2,3-triazol-4-yl]methoxy}olean-18-en-28-oic acid (**6d**)

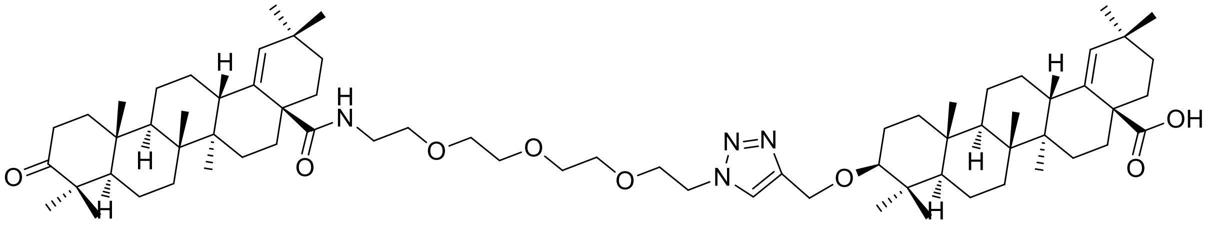

Procedure G was applied, using **5d** as the starting compound, yielding **6d** in a 93 % yield.

^{1}H NMR: δ 0.73 (1H, dd, J_1=1.8 Hz, J_2=11.5 Hz, H5''), 0.74 (3H, s, H26''), 0.76 (3H, s, H27), 0.77 (3H, s, H25''), 0.90 (3H, s, H24''), 0.90 (3H, s, H29''), 0.91 (3H, s, H23''), 0.92 (3H, s, H30''), 0.93 (3H, d, *J*=0.7 Hz, H25), 0.97 (3H, s, H30), 0.97 (3H, s, H26), 0.99 (3H, s, H29), 1.02 (3H, s, H24), 1.07 (3H, s, H23), 1.12 (3H, s, H27''), 1.82-1.90 (2H, m, H2''), 1.83 (2H, ddd, J_1=3.5 Hz, J_2=4.8 Hz, J_3=13.8 Hz, H22), 1.94 (2H, ddd, J_1=5.1 Hz, J_2=7.5 Hz, J_3=12.8 Hz, H1), 2.12-2.17 (1H, m, H13), 2.44-2.53 (2H, m, H2), 2.82 (1H, dd, J_1=4.2 Hz, J_2=14.0 Hz, H18''), 3.00 (1H, dd, J_1=4.3 Hz, J_2=11.7 Hz, H3''), 3.36-3.42 (2H, m, H1'), 3.47-3.52 (2H, m, H1'), 3.49-3.62 (2H, m, H3'), 3.49-3.62 (2H, m, H2'), 3.49-3.62 (2H, m, H5'), 3.49-3.62 (2H, m, H4'), 3.49-3.62 (2H, m, H6'), 3.90 (2H, t, *J*=5.1 Hz, H7'), 4.59-4.62 (2H, m, H8'), 4.67 (2H, d, *J*=13.2 Hz, H11'), 4.88 (2H, d, *J*=13.2 Hz, H11'), 5.03 (1H, t, *J*=3.8 Hz, H12''), 5.30 (1H, dd, J_1=0.7 Hz, J_2=1.8 Hz, H19), 7.85 (1H, s, H9').

^{13}C NMR: δ 14.80 (q, C27), 15.33 (q, C24''), 15.86 (q, C26), 16.47 (q, C25''), 16.61 (q, C25), 17.09 (q, C26''), 18.22 (t, C6''), 19.58 (t, C6), 20.86 (q, C24), 21.56 (t, C11), 22.58 (t, C16''), 22.93 (t, C11''), 23.39 (t, C2''), 23.55 (q, C30''), 25.90 (q, C27''), 26.27 (t, C12), 26.89 (q, C23), 27.65 (st, C15''), 28.19 (q, C23''), 28.93 (q, C30), 29.27 (t, C15), 30.66 (s, C20''), 30.90 (q, C29), 32.29 (t, C21), 32.40 (t, C22), 32.40 (t, C22''), 32.60 (t, C7''), 33.05 (q, C29''), 33.17 (s, C20), 33.65 (t, C7), 33.78 (t, C21''), 33.94 (t, C16), 33.94 (t, C2), 36.86 (s, C10), 36.98 (s, C10''), 38.06 (t, C1''), 38.69 (s, C4''), 39.26 (s, C8''), 39.35 (t, C1'), 39.87 (t, C1), 40.54 (s, C8), 41.03 (d, C18''), 41.60 (s, C14''), 41.66 (d, C13), 42.63 (s, C14), 45.77 (d, C5), 45.84 (t, C19''), 46.43 (s, C17''), 47.24 (s, C4), 47.51 (d, C9''), 48.19 (s, C17), 50.38 (d, C9), 51.59 (t, C8'), 55.48 (d, C5''), 61.79 (t, C11'), 68.96 (t, C7'), 70.28 (t, C6'), 70.31 (t, C5'), 70.43 (t, C4'), 70.51 (t, C3'), 70.57 (t, C2'), 87.19 (d, C3''), 122.52 (d, C12''), 124.76 (d, C9'), 135.69 (d, C19), 138.33 (s, C18), 143.60 (s, C13''), 144.73 (s, C10'), 175.56 (s, C28), 181.96 (s, C28''), 218.48 (s, C3).

MS (ES): *m/z* = 1150.1 $[M+H]^+$; 1148.1 $[M-H]^-$

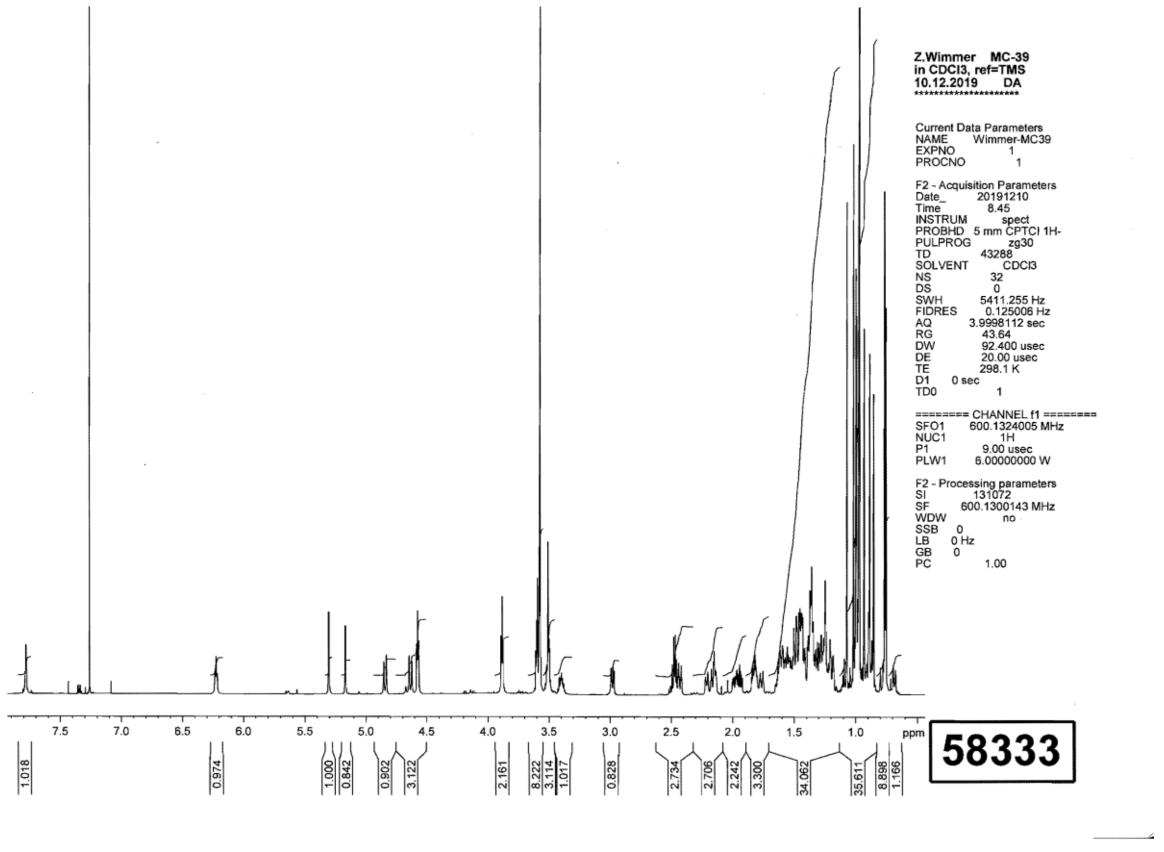

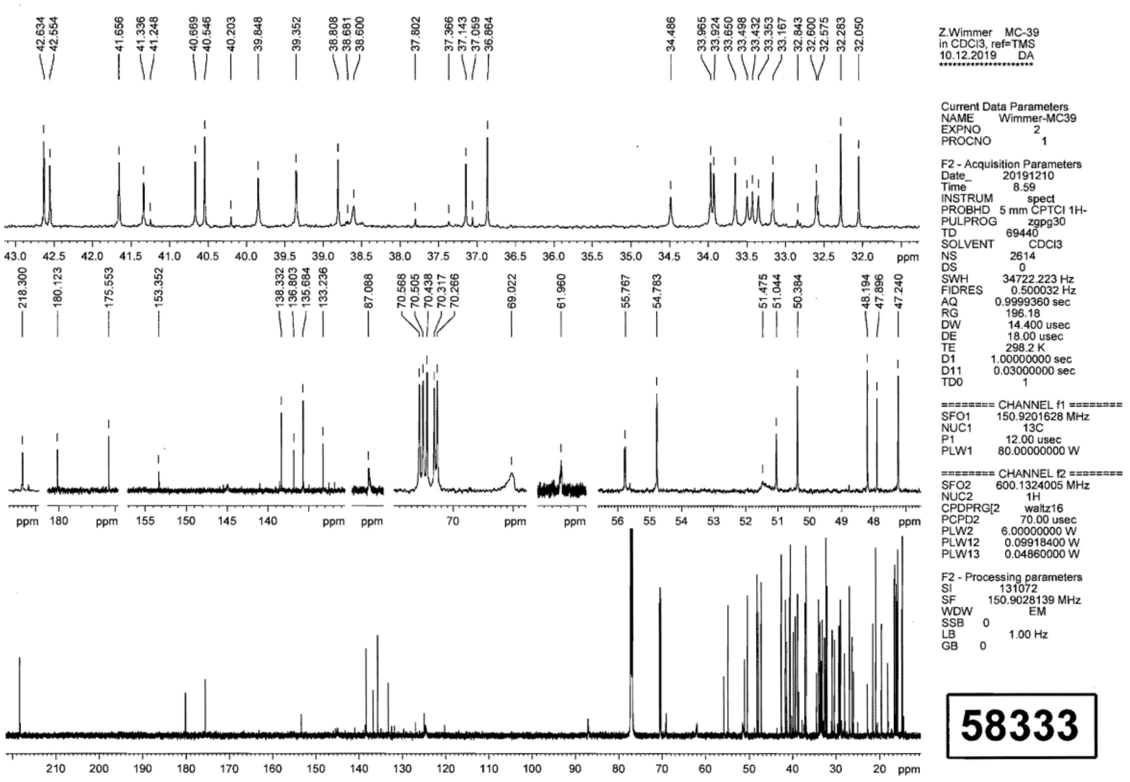

1.30. (3β)-28-[(2-{2-[2-(2-{4-[({(3β)-28-[(2-{2-[2-(2-Azidoethoxy)ethoxy]ethoxy}ethyl)-amino]-28-oxoolean-18-en-3-yl}oxy)methyl]-1*H*-1,2,3-triazol-1-yl}ethoxy)ethoxy]ethoxy}-ethyl)amino]-28-oxoolean-18-en-28-oate (**7d**)

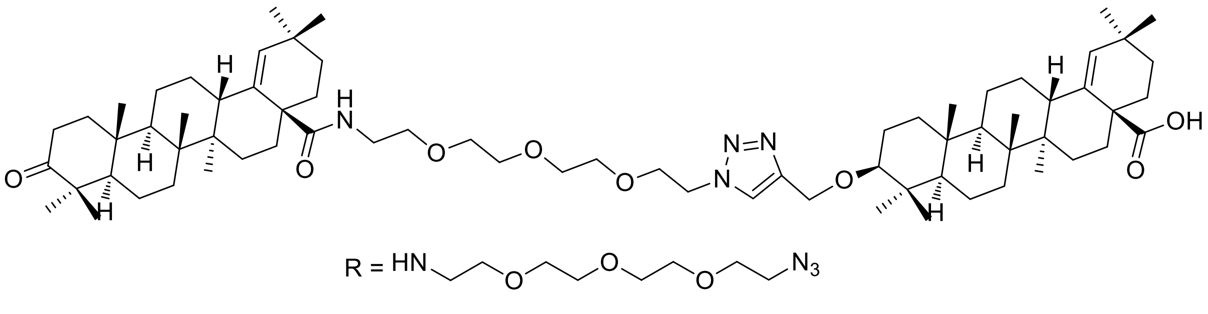

Procedure E was applied, using **6d** as the starting compound, yielding **7d** in a 67 % yield.

^{1}H NMR: δ 0.69 (1H, dd, J_1=2.0 Hz, J_2=11.2 Hz, H5"), 0.75 (3H, s, H27), 0.76 (3H, s, H25"), 0.77 (3H, s, H27"), 0.86 (3H, s, H24"), 0.90 (3H, s, H23"), 0.93 (3H, d, *J*=0.7 Hz, H25), 0.96 (3H, s, H26"), 0.97 (3H, s, H26), 0.98 (3H, s, H30), 0.98 (3H, s, H30"), 1.00 (3H, s, H29), 1.01 (3H, s, H29"), 1.02 (3H, s, H24), 1.07 (3H, s, H23), 1.78 (2H, dt, J_1=3.4 Hz, J_2=3.4 Hz, J_3=13.2 Hz, H1"), 1.83 (2H, ddd, J_1=3.5 Hz, J_2=5.0 Hz, J_3=13.6 Hz, H22), 1.95 (2H, ddd, J_1=5.3 Hz,

J_2=7.3 Hz, J_3=12.8 Hz, H1), 2.11-2.15 (1H, m, H13), 2.11 (1H, m, H13''), 2.42 (2H, dt, J_1=3.5 Hz, J_2=3.5 Hz, J_3=13.6 Hz, H16''), 2.46 (2H, ddd, J_1=5.3 Hz, J_2=8.3 Hz, J_3=15.8 Hz, H2), 2.49 (2H, ddd, J_1=7.3 Hz, J_2=8.9 Hz, J_3=15.8 Hz, H2), 3.01 (2H, dd, J_1=4.5 Hz, J_2=11.8 Hz, H2''), 3.37-3.40 (2H, m, H8*), 3.40-3.45 (2H, m, H1'), 3.40-3.48 (2H, m, H1*), 3.49-3.53 (2H, m, H1'), 3.56-3.68 (2H, m, H2'), 3.56-3.68 (2H, m, H2*), 3.56-3.68 (2H, m, H5*), 3.56-3.68 (2H, m, H4*), 3.56-3.68 (2H, m, H3*), 3.56-3.68 (2H, m, H3'), 3.56-3.68 (2H, m, H7*), 3.56-3.68 (2H, m, H6'), 3.56-3.68 (2H, m, H4'), 3.56-3.68 (2H, m, H5'), 3.56-3.68 (2H, m, H6*), 3.92-3.95 (2H, m, H7'), 4.63-4.67 (2H, m, H8'), 4.78 (2H, d, *J*=13.2 Hz, H11'), 5.00 (2H, d, *J*=13.2 Hz, H11'), 5.31 (1H, dd, J_1=0.8 Hz, J_2=1.8 Hz, H19), 5.31 (1H, dd, J_1=0.7 Hz, J_2=1.8 Hz, H19''), 8.02 (1H, s, H9').

^{13}C NMR: δ 14.80 (q, C27), 14.91 (q, C27''), 15.86 (q, C26''), 16.03 (q, C26), 16.59 (q, C25), 16.72 (q, C24''), 17.29 (q, C25''), 18.12 (t, C6''), 19.59 (t, C6), 20.88 (q, C24), 21.02 (t, C11''), 21.56 (t, C11), 22.71 (t, C2''), 24.64 (s, C14), 26.02 (t, C12''), 26.26 (t, C12), 26.88 (q, C23), 28.03 (q, C23''), 28.93 (q, C30''), 28.93 (q, C30), 29.27 (t, C15), 29.33 (t, C15''), 30.88 (q, C29''), 30.90 (q, C29), 32.28 (s, C20''), 32.29 (s, C20), 32.61 (t, C16''), 32.70 (t, C16), 33.18 (t, C21), 33.18 (t, C21''), 33.65 (t, C7), 33.94 (t, C22), 33.94 (t, C22''), 33.98 (t, C2), 34.42 (t, C7''), 36.87 (s, C10), 37.14 (s, C10''), 38.59 (s, C8''), 38.79 (t, C1*), 39.33 (t, C1''), 39.36 (t, C1'), 39.83 (t, C1), 40.55 (s, C8), 40.70 (s, C14''), 41.53 (d, C13''), 42.61 (s, C4''), 42.66 (d, C13), 47.22 (s, C4), 48.20 (s, C17), 48.21 (s, C17''), 50.38 (d, C9), 50.65 (t, C8*), 51.04 (d, C9''), 51.72 (t, C8'), 54.77 (d, C5), 55.76 (d, C5''), 61.65 (t, C11'), 68.93 (t, C7'), 70.07 (t, C7*), 70.26 (t, C6*), 70.31 (t, C5*), 70.35 (t, C4*), 70.37 (t, C3*), 70.45 (t, C2*), 70.53 (t, C6'), 70.57 (t, C5'), 70.59 (t, C4'), 70.63 (t, C3'), 70.68 (t, C2'), 87.14 (d, C3''), 125.00 (d, C9'), 135.61 (d, C19), 135.62 (d, C19''), 138.35 (s, C18''), 138.40 (s, C18), 144.62 (s, C10'), 175.54 (s, C28''), 175.55 (s, C28), 218.12 (s, C3).

MS (ES): *m/z* = 1350.3 $[M+H]^+$

1.31. [1-(2-{2-[2-(2-{[(3β)-3-{[1-(2-{2-[2-(2-{[3,28-Dioxoolean-18-en-28-yl]amino}ethoxy)-ethoxy]ethoxy}ethyl)-1*H*-1,2,3-triazol-4-yl]methoxy}-28-oxoolean-18-en-28-yl]amino}-ethoxy)ethoxy]ethoxy}ethyl)-1*H*-1,2,3-triazol-4-yl]methyl 3-oxoolean-18-en-28-oate (**8d**)

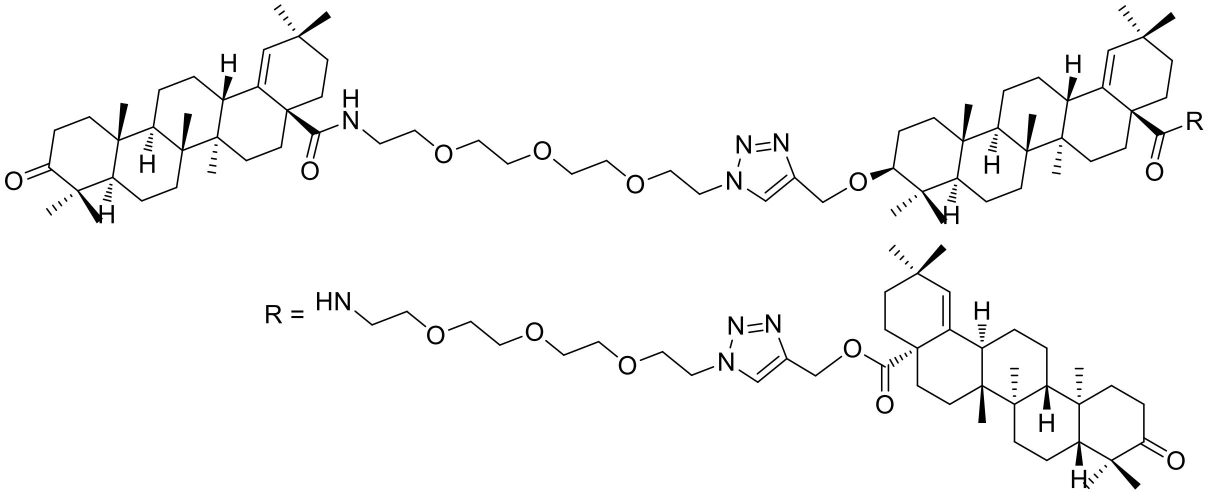

Procedure F was applied, using **7d** and **2c** as the starting compounds, yielding **8d** in a 99 % yield.

^{1}H NMR: δ 0.68 (dd, J_1=2.1 Hz, J_2=11.5 Hz, H5''), 0.74 (s, H27''), 0.75 (s, H27), 0.75 (s, H27*), 0.77 (s, H26), 0.78 (s, H26*), 0.85 (s, H23''), 0.88 (s, H26''), 0.91 (s, H24''), 0.92 (d, J=0.6 Hz, H25*), 0.93 (d, J=0.7 Hz, H25), 0.94 (s, H25''), 0.95 (s, H30*), 0.97 (s, H29*), 0.98 (s, H30''), 0.98 (s, H30), 1.00 (s, H29''), 1.00 (s, H29), 1.01 (s, H24*), 1.02 (s, H24), 1.06 (s, H23*), 1.07 (s, H23), 1.87 (ddd, J_1=4.3 Hz, J_2=5.1 Hz, J_3=13.2 Hz, H1''), 1.93 (ddd, J_1=4.8 Hz, J_2=76.0 Hz, J_3=13.0 Hz, H1*), 1.95 (ddd, J_1=5.2 Hz, J_2=7.2 Hz, J_3=12.8 Hz, H1), 2.99 (dd, J_1=4.4 Hz, J_2=11.7 Hz, H3''), 3.38-3.42 (m, H1'''), 3.38-3.42 (m, H1'), 3.46-3.53 (m, H1'''), 3.46-3.53 (m, H1'), 3.86 (t, J=5.2 Hz, H7'''), 3.89-3.92 (m, H7'), 4.50-4.53 (m, H8'''), 4.59-4.62 (m, H8'), 4.67 (d, J=12.8 Hz, H11'), 4.67 (d, J=12.8 Hz, H11'''), 4.90 (d, J=12.8 Hz, H11'), 4.90 (d, J=12.8 Hz, H11'''), 5.11 (d, J=1.4 Hz, H19*), 5.31 (bd, J=1.6 Hz, H19), 7.73 (s, H9'), 7.89 (bs, H9''').

^{13}C NMR: δ 14.80 (C27), 14.85 (C27*), 14.92 (C27''), 15.39 (C26*), 15.86 (C26''), 16.03 (C26), 16.52 (C25), 16.59 (C25*), 16.73 (C25''), 18.11 (C6''), 19.57 (C6), 19.59 (C6*), 20.88 (C24), 20.91 (C24*), 21.01 (C11''), 21.02 (C24''), 21.43 (C11*), 21.56 (C11), 22.67 (C2''), 25.88 (C12''), 26.23 (C12*), 26.26 (C12), 26.82 (C23), 26.89 (C23*), 28.03 (C23''), 28.94 (C30''), 28.94 (C30), 29.03 (C30*), 29.26 (C15''), 29.27 (C15), 29.32 (C15*), 30.41 (C29*), 30.91 (C29), 30.91 (C29''), 31.98 (C21*), 32.29 (C21), 32.29 (C21''), 32.67 (C16''), 33.18 (C20), 33.19 (C20''), 33.22 (C20*), 33.41 (C22*), 33.41 (C7), 33.66 (C22), 33.66 (C7*), 33.94 (C16*), 33.94 (C16), 33.98 (C2), 34.02 (C2*), 34.03 (C22''), 34.40 (C7''), 36.86 (C10), 36.87 (C10*), 37.13 (C10''), 38.57 (C8''), 38.78 (C1''), 39.32 (C1'), 39.36 (C1'''), 39.78 (C1*), 39.83 (C1), 40.44 (C8*), 40.55 (C8), 40.70 (C4''), 40.70 (C14''), 41.23 (C13*), 41.55 (C13''), 41.67

(C13), 42.44 (C14*), 42.62 (C14), 47.22 (C4*), 47.22 (C4), 48.04 (C17*), 48.20 (C17), 48.20 (C17''), 50.35 (C9*), 50.38 (C9), 51.01 (C9''), 51.84 (C8'''), 51.97 (C8'), 54.76 (C5), 54.79 (C5*), 55.73 (C5''), 57.37 (C11'''), 61.78 (C11'), 68.83 (C7'), 69.33 (C7'''), 70.31 (C5'''), 70.31 (C6'''), 70.31 (C6'), 70.33 (C5'), 70.45 (C4'''), 70.48 (C4'), 70.53 (C3'), 70.53 (C3'''), 70.54 (C2'), 70.59 (C2'''), 87.18 (C3''), 124.98 (C9'), 125.19 (C9'''), 132.86 (C19''), 135.60 (C19*), 135.68 (C19), 136.69 (C18''), 138.33 (C18), 138.43 (C18*), 142.77 (C10'''), 144.78 (C10'), 175.56 (C28), 175.75 (C28''), 176.50 (C28*), 218.12 (C3), 218.19 (C3*).

MS (ES): *m/z* = 1842.3 [M+H]$^+$

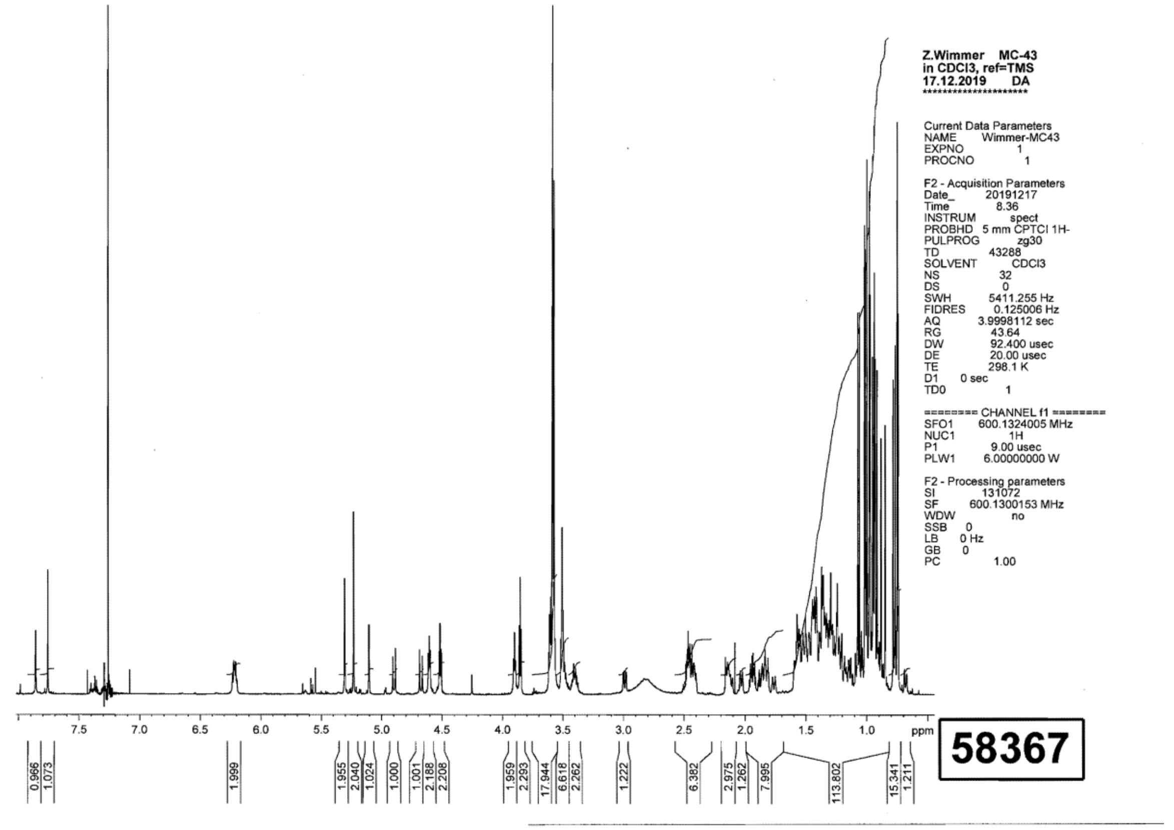

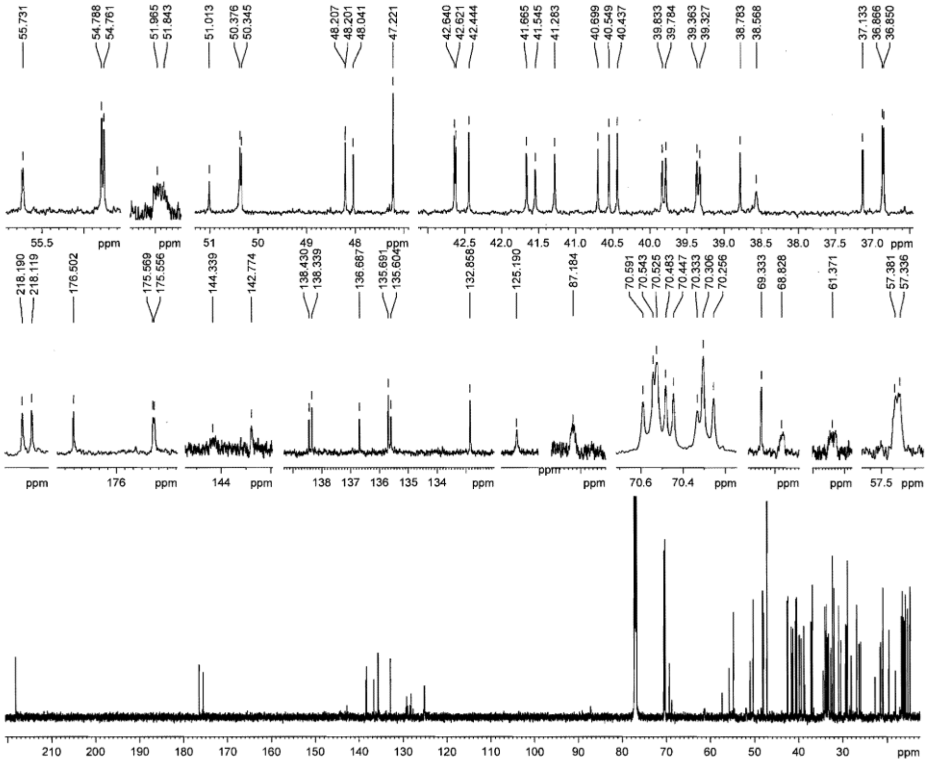

Z.Wimmer MC-43
in CDCl3, ref=TMS
17.12.2019 DA
****

Current Data Parameters
NAME Wimmer-MC43
EXPNO 2
PROCNO 1

F2 - Acquisition Parameters
Date_ 20191217
Time 11.36
INSTRUM spect
PROBHD 5 mm CPTCI 1H-
PULPROG zgpg30
TD 69440
SOLVENT CDCl3
NS 3596
DS 0
SWH 34722.223 Hz
FIDRES 0.500032 Hz
AQ 0.9999360 sec
RG 196.18
DW 14.400 usec
DE 18.00 usec
TE 298.2 K
D1 1.00000000 sec
D11 0.03000000 sec
TD0 1

======== CHANNEL f1 ========
SFO1 150.9201628 MHz
NUC1 13C
P1 12.00 usec
PLW1 80.00000000 W

======== CHANNEL f2 ========
SFO2 600.1324005 MHz
NUC2 1H
CPDPRG[2 waltz16
PCPD2 70.00 usec
PLW2 6.00000000 W
PLW12 0.09918400 W
PLW13 0.04860000 W

F2 - Processing parameters
SI 131072
SF 150.9028135 MHz
WDW EM
SSB 0
LB 1.00 Hz
GB 0

58367

2. Antimicrobial screening tests

Antimicrobial activity of the target molecular ribbons **8a**, **12a**, **8b**, **12b**, **4c**, **6c**, **6d** and **8d**, and that of **1a**–**1c** was studied *in vitro*, using *Bacillus cereus*, *Escherichia coli*, *Pseudomonas aeruginosa*, *Staphylococcus aureus* and *Enterococcus faecalis*. The resazurine test was used to evaluate this activity.

Table S1 shows the complete data on antimicrobial activity of the target triterpenoid molecular ribbons which have been achieved in this investigation.

Table S1. Antimicrobial activity, inhibition of the microorganism [%]

Comp.	*Staphylococcus aureus*			Comp.	*Escherichia coli*		
	c = 100 μg·mL^{-1}	c = 50 μg·mL^{-1}	c = 25 μg·mL^{-1}		c = 100 μg·mL^{-1}	c = 50 μg·mL^{-1}	c = 25 μg·mL^{-1}
**8a**	68 ± 10.2	55 ± 8.3	52 ± 7.8	**8a**	-	24 ± 3.6	51 ± 7.7
**8b**	77 ± 11.6	24 ± 3.6	–	**8b**	31 ± 4.7	25 ± 3.8	17 ± 2.6
**12a**	59 ± 8.9	52 ± 7.8	28 ± 4.2	**12a**	-	-	-
**12b**	59 ± 8.9	33 ± 5.0	40 ± 6.0	**12b**	-	-	-
**4c**	76 ± 11.4	66 ± 9.9	11 ± 1.7	**4c**	31 ± 4.7	34 ± 5.1	25 ± 3.8
**6c**	27 ± 4.0	51 ± 7.7	13 ± 2.0	**6c**	30 ± 4.5	18 ± 2.7	10 ± 1.5
**6d**	-	31 ± 4.7	25 ± 3.8	**6d**	-	13 ± 2.0	9 ± 1.4
**8d**	19 ± 2.9	19 ± 2.9	11 ± 1.7	**8d**	14 ± 2.1	17 ± 2.6	12 ± 1.8
**1a**	33 ± 5.0	8 ± 1.2	–	**1a**	-	-	-
**1b**	47 ± 7.0	17 ± 2.6	21 ± 3.2	**1b**	-	2 ± 0.3	-
**1c**	10 ± 1.5	3 ± 0.5	–	**1c**	-	-	-

Comp.	*Bacillus cereus*			Comp.	*Pseudomonas aeruginosa*		
	c = 100 μg·mL^{-1}	c = 50 μg·mL^{-1}	c = 25 μg·mL^{-1}		c = 100 μg·mL^{-1}	c = 50 μg·mL^{-1}	c = 25 μg·mL^{-1}
**8a**	-	-	-	**8a**	14 ± 2.1	17 ± 2.6	21 ± 3.2
**8b**	-	38 ± 5.7	16 ± 2.4	**8b**	60 ± 9.0	59 ± 8.9	33 ± 5.0
**12a**	-	7 ± 1.0	4 ± 0.6	**12a**	-	-	-
**12b**	5 ± 8.0	13 ± 2.0	12 ± 1.8	**12b**	35 ± 5.3	8 ± 1.2	11 ± 1.7
**4c**	6 ± 9.0	-	2 ± 0.3	**4c**	46 ± 6.8	47 ± 7.0	50 ± 7.5
**6c**	13 ± 2.0	19 ± 2.9	7 ± 1.0	**6c**	60 ± 9.0	69 ± 10.4	12 ± 1.8

**6d**	3 ± 0.5	-	1 ± 0.2	**6d**	-	-	-
**8d**	5 ± 8.0	10 ± 1.5	13 ± 2.0	**8d**	-	9 ± 1.4	16 ± 2.4
**1a**	77 ± 11.6	77 ± 11.6	76 ± 11.4	**1a**	-	-	-
**1b**	75 ± 11.3	76 ± 11.4	78 ± 11.7	**1b**	-	-	-
**1c**	66 ± 9.9	15 ± 2.3	-	**1c**	-	-	-

Comp.	*Enterococcus faecalis*			Comp.	*Enterococcus faecalis*		
	c = 100 µg·mL^{-1}	*c* = 50 µg·mL^{-1}	*c* = 25 µg·mL^{-1}		*c* = 100 µg·mL^{-1}	*c* = 50 µg·mL^{-1}	*c* = 25 µg·mL^{-1}
**8a**	32 ± 4.8	28 ± 4.2	17 ± 2.6	**6d**	28 ± 4.2	14 ± 2.1	2 ± 0.3
**8b**	54 ± 8.1	43 ± 6.5	20 ± 3.0	**8d**	23 ± 3.5	33 ± 5.0	37 ± 5.6
**12a**	2 ± 0.3	-	-	**1a**	89 ± 13.4	90 ± 13.5	87 ± 13.0
**12b**	-	-	-	**1b**	69 ± 10.4	64 ± 9.6	38 ± 5.7
**4c**	43 ± 6.5	50 ± 7.5	62 ± 9.3	**1c**	-	-	-
**6c**	36 ± 5.4	25 ± 3.8	-				

3. Gel formation and supramolecular self-assembly

Among the studied series of compounds, the only **4c**, **6c** and **6d** produced a gel in glycerol, **8a** in ethylene glycol, which is shown in Figure S1 as an example.

Figure S1. Gel of **8a** in ethylene glycol.

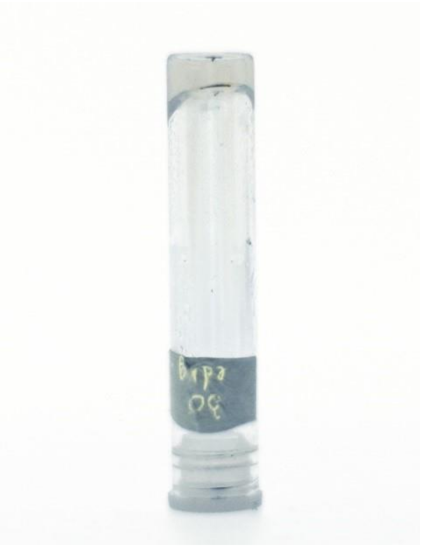

Supramolecular self-assembly was studied on the basis of the UV-VIS spectra of the compounds in several methanol/water mixtures stepwise during the time interval 0-140 h. Here we show compounds **8a**, **12a**, **12b** and **6d** as examples, with which the irregularities in the UV-VIS spectra were either well pronounced (**8a** and **12a**; Figure S2), or no substantial irregularity was detected (**12b** and **6d**).

Figure S2. UV-VIS spectra of **8a** (top) and **12a** (bottom) measured in the interval 0-140 h.

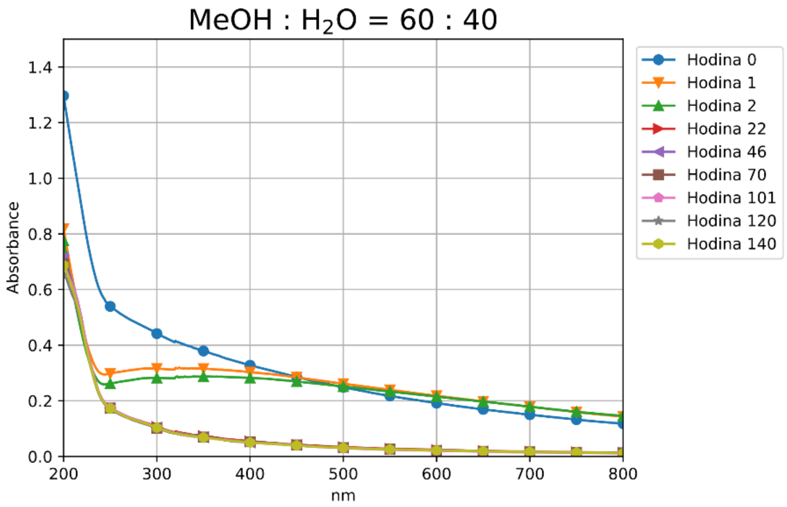

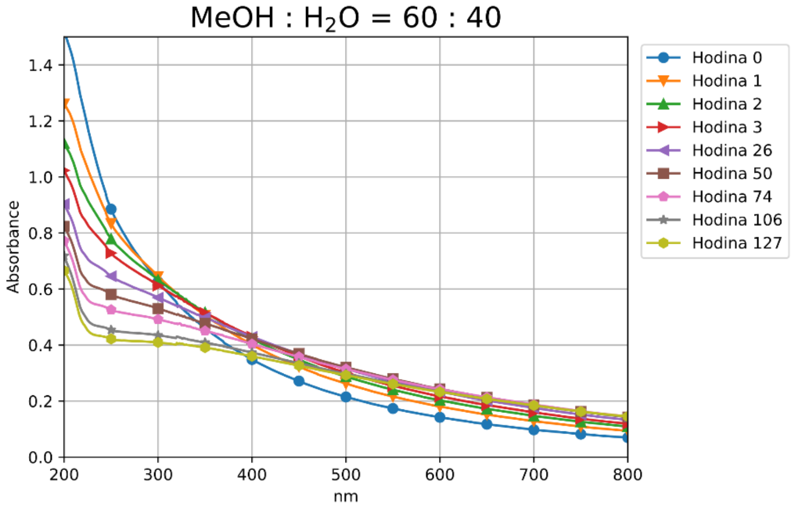

Compound **8a**: Irregularity was detected in hours 1 and 2 at the methanol / water (60 / 40) ratio. During that time interval a new maximum appeared at 300 nm wavelength, and the shape of the curves is different than in any of the other intervals of measurement.

Compound **12a**: A sequence of decreasing absorbance value is regular with increasing time of measurement up to 300 – 350 nm wavelength. Irregularities were detected in the area above 350 nm wavelength.

Compound **12b**: No substantial irregularity was detected.

Compound **6d**: No substantial irregularity was detected.
